# Neurologic complications after allogeneic transplantation: a meta‐analysis

**DOI:** 10.1002/acn3.50909

**Published:** 2019-09-27

**Authors:** Maria Gavriilaki, Maria Mainou, Eleni Gavriilaki, Anna‐Bettina Haidich, Sotirios Papagiannopoulos, Ioanna Sakellari, Achilles Anagnostopoulos, Vasilis Kimiskidis

**Affiliations:** ^1^ Laboratory of Clinical Neurophysiology AHEPA Hospital Aristotle University of Thessaloniki Thessaloniki Greece; ^2^ Clinical Research and Evidence‐Based Medicine Unit Second Medical Department Aristotle University of Thessaloniki Thessaloniki Greece; ^3^ Hematology Department‐BMT Unit G. Papanicolaou Hospital Thessaloniki Greece; ^4^ Department of Hygiene, Social and Preventive Medicine and Medical Statistics Medical School Aristotle University of Thessaloniki Thessaloniki Greece; ^5^ Neurology Department G. Papanicolaou Hospital Thessaloniki Greece

## Abstract

**Objective:**

Neurologic adverse events remain challenging complications with poor morbidity and mortality post adult allogeneic hematopoietic cell transplantation (allo‐HCT) for hematologic diseases. We conducted a systematic review and meta‐analysis to determine their spectrum, incidence, and impact on survival**.**

**Methods:**

We searched MEDLINE, COCHRANE, EMBASE through March 2019 for all types of primary studies. Two independent reviewers screened, extracted data, and assessed risk of bias (RoB).

**Results:**

We identified 552 eligible studies describing 57.972 patients; one randomized controlled trial, two case–control, 17 prospective, 86 retrospective cohort studies, 21 case series, and 425 case reports. RoB ranged from fair to high although case series were low‐risk. The majority of studies traced infectious or drug‐related neurologic manifestations. Infectious complications were present in 2.7% (95% CI 1.9–3.6) and 3.3% (95% CI 0.8–7.1) of patients in retrospective and prospective cohort studies, respectively. In retrospective studies, 3.4% (95% CI 2.1–4.9) of patients suffered from drug‐related neurologic events. In prospective cohorts the equivalent incidence was 13% (95% CI 4.2–24.8). Neurologic complications had a detrimental impact on survival.

**Interpretation:**

Our study highlights the wide spectrum and significant impact of neurologic complications on survival post allo‐HCT. This systematic review summarizes existing data and provides the necessary background information for every physician involved in the management of these patients.

## Introduction

Allogeneic hematopoietic cell transplantation (allo‐HCT) is a compelling therapy with the potential of cure not only for numerous hematologic malignancies but also for a wide spectrum of disorders of the immune system.^1^ The theoretical basis of allo‐HCT is the intravenous infusion of progenitor cells capable to establish long‐term, stable myeloid, hematologic, and immunologic functions. The role of allo‐HCT has been established over the years. Therefore, its worldwide use is experiencing an upturn with alternative sources or donors and a significant improvement in supportive care. Despite advances in medical research and practice and use of less toxic agents allo‐HCT leads to complications with increased morbidity and mortality.^2^

Among them, neurologic adverse events remain a largely understudied type of complication, despite their clinical impact and potential poor morbidity and mortality.^3^ A significant proportion of allo‐HCT complications concerns the nervous system both the central (CNS) or the peripheral (PNS).^4^ Drug toxicity, fungal infections, and immune‐mediated disorders prevail among other etiological factors.^5^ Furthermore, their severity varies from mild to fatal disorders.^4^, ^6^

Those complications need to be recognized first by transplanters–hematologists. However, the problem lies in the vast number of various neurologic complications which either have been underestimated or not yet described systematically. The majority of existing studies focuses on a heterogeneous study population, for example, both adults and children, including allogeneic and autologous HCT.^7^, ^8^, ^9^ There is an unmet need to categorize these complications, describe their incidence and acknowledge their significant impact on survival.

The primary purpose of this systematic review and meta‐analysis is to provide a thorough summary of the incidence of the wide spectrum of neurologic complications after allo‐HCT for hematologic diseases in adults that have been scarcely described in literature. The secondary purpose is to assess the impact of those complications on overall survival.

## Methods

This systematic review is reported in accordance with the Preferred Reporting Items for Systematic reviews and Meta‐Analysis guidelines (Table S1).^10^ A more thorough description of methods can be found in supplement part I (online only).

### Search

We conducted an extensive systematic literature search through 17 March, 2019 (Table S2). Databases included PubMed, Embase, and Cochrane. The search was limited in studies published in English.

### Eligibility criteria

The eligibility criteria were as follows: (a) adult population specified as patients 15 years old or older,^11^ (b) allo‐HCT for hematologic diseases, (c) neurologic disorders’ diagnosis based on patient record. We deemed eligible all types of primary studies. We excluded only editorials and narrative reviews.

### Data collection process and risk of bias

All titles were downloaded into an Endnote library and then uploaded into Covidence. Duplicates were removed automatically. Two reviewers (MG, EG) working independently screened the titles and abstracts for eligibility. Then, full text was screened again in duplicate. A senior reviewer (MM) resolved any disagreement. For each article, at least one reviewer extracted the following: baseline characteristics of study and population, incidence and type of complication, time of the event after allo‐HCT, follow‐up, intervention, and outcome of neurologic event.

Two reviewers (MG, EG) independently assessed the quality of included studies. We divided studies according to their type: RCTs, cohort and case–control studies, case series, and case reports. We excluded case reports from further evaluation. We present them descriptively in the results and thoroughly in Table S3 (online only).

We assessed case series according to a tool proposed by Murad et al;^12^ case–control and cohort studies using the Newcastle–Ottawa scale (NOS);^13^ randomized trials using the Cochrane risk of bias assessment tool.^14^ The quality of evidence of the review was evaluated using the Grading of Recommendations Assessment, Development, and Evaluation (GRADE) tool.^15^

### Summary measures and synthesis of results

The primary outcome was assessed in a form of a proportion of adult patients with the neurologic manifestation (n) from the total group of patients that received allo‐HCT for hematologic diseases (N). We performed meta‐analysis of proportions using the Freeman–Tukey double arcsine transformation,^16^ including only cohort studies. The number of available studies (≥5) and homogeneity of the included populations were the key parts for safe results.^17^ We chose random‐effects meta‐analysis. Heterogeneity was observed visually in the forest plot and quantified by the estimation of *I* ^2^ measure.^18^ Publication bias was assessed using Egger’s test and visually inspecting produced funnel plot, although limitations of all methods in this occasion are considerable.^19^ Analyses were performed using OpenMeta[Analyst] or Stata®(version 11).^20^, ^21^

For the secondary endpoint no synthesis of results was performed, since the data were expectedly diverse. Thus, we provide results from each study type separately, without excluding any type. The definition of case fatality rate was the proportion of patients who died because of neurologic complications from the patients that presented neurologic signs or symptoms after allo‐HCT.

### Sensitivity and subgroup analyses

We have preplanned to perform a sensitivity analysis integrating the risk of bias assessment by excluding studies evaluated as high risk. We also have prespecified subgroup analyses according to the type of neurologic event.

## Results

### Study selection – flow diagram

**Figure 1:**
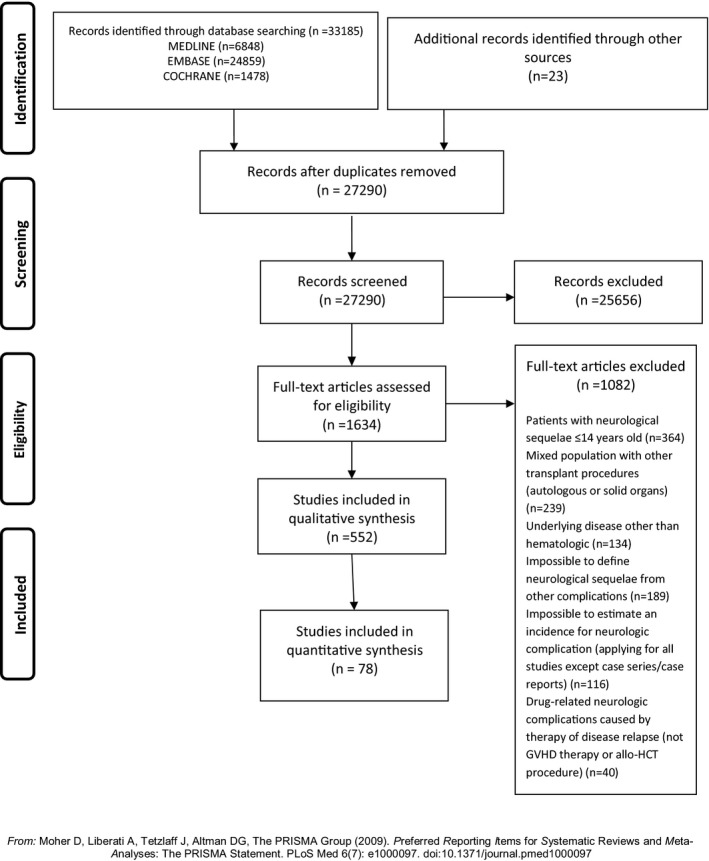
PRISMA Flow Diagram.

We retrieved a total of 33185 references which were imported for screening. The entire systematic literature review and selection of final records is illustrated by a PRISMA flow diagram in Figure 1.

Taking into consideration the inclusion criteria mentioned above, our systematic review included one RCT, two case–control studies, 17 prospective cohort, 86 retrospective cohort studies, 21 case series, and 425 case reports. The total number of patients reported in these 552 references was 57.972. Of note, one cohort study by Colombo et al^22^ was retrospective from 1997 until 2007 and then prospective from 2008 to 2012 but the results of those time periods were presented together in the reports and could not provide separate data. Thus, we included it in retrospective cohorts.

### Descriptive characteristics of each study category

The included study categories were RCTs, case–control, case series, prospective and retrospective cohort studies as described before. Detailed characteristics of these 127 studies are presented in Tables S4, S5 and S6 (online only). There was one RCT by Irle et al^23^ that reported drug‐related neurologic complications of methotrexate and cyclosporine as part of transplant‐related complications. Also, there were two case–control studies; one related to protozoan infectious CNS involvement^24^ and the other to TA‐TMA‐related neurologic events.^25^

Among 17 prospective cohort studies identified, seven referred to drug‐related complications. Ishiyama et al^26^, ^27^ analyzed the safety of foscarnet sodium and the possibility of development of HHV‐6 encephalitis in two studies carried out in different periods. De Lima et al^28^ and Christopoulos et al^29^ described treatment‐related neurologic complications among other adverse effects of different conditioning regimens for allo‐HCT; whereas DeFilipp^30^ and Kroger^31^ reported complications of PNS as well as a patient presented with PRES associated with brentuximab vedotin and lenalidomide used as graft‐versus‐host disease (GVHD) treatment and maintenance, respectively. Van der Wagen et al reported two cases (one viral encephalitis and one Guillain–Barre syndrome) related to the use of rituximab followed by nilotinib as treatment strategy for sclerotic GVHD.^32^ Regarding the rest ten prospective studies, immune‐mediated and PNS complications as neuromuscular manifestations of GVHD were examined by Koeppen et al.^33^ Beglinger^34^ and Scherwath^35^ investigated a different type of neurotoxicity of allo‐HCT, that is, cognitive impairment and higher cortical functions. Sostak et al^36^ prospectively evaluated a variety of neurologic complications (infectious, cerebrovascular, metabolic, and PNS) after allo‐HCT. Goto et al^37^ conducted a prospective observational study on patients treated with peripheral blood stem cell transplantation from unrelated donors and reported a patient’s death due to central nervous system disorder. Five additional studies investigated neurologic involvement in infectious diseases caused by protozoa, bacteria, or virus. Finally, in seven of 17 prospective cohorts, neurologic symptoms have been considered to occur early posttransplant. However, this may not be a safe conclusion since not only the follow‐up period is limited in these studies, but also six of them do not report the time that neurologic event occurred. The median follow‐up period of prospective cohort studies was 6 months as estimated by available data from 16 studies.

We classified retrospective cohort studies, respectively, according to the type of neurologic complications. Interestingly, 23 of 86 retrospective studies described more than one type of complication or an encephalopathy of unknown origin making the classification more complex. We found 23 studies exclusively referring to infections and in particular, 13 viral, three fungal, four protozoan, one bacterial and two were about various infections. Sixteen records revealed drug‐related neurologic symptoms. Relapse in CNS was studied in nine records. In addition, only five studies were interested in immune‐mediated and cerebrovascular complications, respectively. TA‐TMA was presented separately only in three studies; whereas PTLD and peripheral complications only in one each. It should be noted that most retrospective cohort studies represent the experience of a center in the adverse effects of allo‐HCT and sometimes specifically neurologic events. Only 38 of 86 studies reported follow‐up period of their cohort and therefore no safe time associated conclusion can be reached. Nevertheless, authors of 47 studies reported that neurologic complications occurred early versus 24 studies that presented late posttransplant events.

Moving on to case series, as described in methods, we extracted data only for those patients eligible for our systematic review. We tracked down 21 studies that fulfilled the previously described criteria. The majority of them, eight studies, traced infectious neurologic manifestations, six immune‐mediated, four drug‐related, two PTLDs, and one metabolic complication. The causative factor was viral in six of eight infectious studies and either bacterial or fungal in each of the ones remaining. Five of six immune‐mediated studies attributed CNS or PNS signs to GVHD. In the sixth one, Stefanou et al^38^ studied 61 adult patients who underwent allo‐HCT and had inflammatory polyneuropathy or CNS disease. Finally, drugs specifically responsible for neurologic complications according to authors were cyclosporine, tacrolimus, neralabine and liposomal cytarabine, in three different studies (one in each). In 11 of 21 reports neurologic complications were late events; two did not report that information.

### Case reports descriptive characteristics

Taking into consideration the possible publication bias and the vast number of case reports in our systematic review, it would be of value to only describe the variety of the complications presented. As a result, we retrieved 425 case reports: 176 concerned infectious neurologic complications, 109 immune‐mediated, 67 drug‐related mostly related to cyclosporine A or tacrolimus neurotoxicity, 22 relapses of previous hematologic disease, 15 PTLDs, 14 other type, 8 metabolic, 6 TA‐TMA, 4 cerebrovascular neurologic disorders, and 1 disturbance of higher cortical functions. Three references reported infections and one more category (PTLD, TA‐TMA, immune‐mediated) as the cause of the neurologic complication. Overall, 488 patients (≥15 years old) with a neurologic manifestation after allo‐HCT were described. Early posttransplant complications concerned 195 of patients (40%), late 266 (54.5%) of them and in 27 (5.5%) that information was not available. The most common type of neurologic complications was infections in 205 patients (41.9%). The pathogenic cause of infections was viral in 123 (25.2% of total number of patients with neurological manifestations presented as case reports), protozoan in 32 (6.5%), fungal in 34 (6.9%), bacterial in 15 (3.1%) patients and a co‐infection of cerebral toxoplasmosis and HHV‐6 encephalitis in one case (0.2%). The most controversial and recently described^39^ entity of neurologic event after allo‐HCT is associated with GVHD and represents 67 of case reports identified in the literature. According to authors, 74 patients had CNS or PNS diseases attributed to acute but mainly chronic GVHD. Immune‐mediated GVHD‐related neurologic diseases included a variety of phenotypes such as myasthenia gravis, polymyositis, Guillain–Barré, encephalitis, vasculitis, and demyelinating complications.

### Risk of bias within studies

The quality of included studies diverged from low to fair. The score of each study is shown in Tables S4, S5 and S6 (online only). The RCT ranked as high risk applying the Cochrane risk of bias tool.^14^

The maximum NOS score for cohort and case–controls studies was nine stars (four for selection, two for comparability and three for outcome).^13^ Six prospective cohort studies were characterized as fair risk and 11 as high risk of bias. Taking into consideration that the majority of prospective cohorts were designed as prospective follow‐up studies and the comparison was made between patients with or without neurologic complications after allo‐HCT, only one scored two stars in comparability part. The only study that matched age and gender of the cohorts from the onset was Beglinger et al^34^ that, however, recruited healthy not transplanted controls. Therefore, no prospective cohort study was of low risk of bias. Τhe parts that decisively affected the quality of a study, compared with the others included, were the adequacy of the follow‐up of cohorts and the demonstration that no neurologic manifestation was present before transplantation. The application of NOS on retrospective cohort studies revealed limitations on the selection of nonexposed cohort, adequacy of follow‐up, and presence of outcome of interest before allo‐HCT, deeming most of the studies high or fair risk of bias.

From the two case control studies retrieved, one was of fair risk according to NOS and one of low risk of bias. Conrad et al^24^ scored eight of nine stars (low risk) by matching for two factors each case to two controls.

For case series, after the scale proposed by Murad et al^12^ was applied, most were deemed as low risk of bias, whereas only six were of fair risk.

### Incidence of neurologic events

In meta‐analysis of proportions performed, we included only cohort studies, retrospective or prospective, with homogenous population to estimate a proportion. We excluded 25 cohort studies with a total population of unclear age. The number of other studies categories (two case–controls and one RCT) and our definition of case series did not allow their usage in the primary outcome of neurologic complication incidence.

Neurologic clinical signs or symptoms were detected in 1415 of 37450 [6.2% (95% CI 4.8–7.7), *Ι* ^2^ = 96.1% (*P* < 0.001)] patients described in 78 included cohort studies (Figure S1‐online only). In our attempt to draw a secure conclusion and because of high heterogeneity, we present the incidence of subtypes of neurologic diseases after allo‐HCT, whenever that was possible, in retrospective and in prospective cohort studies separately. Heterogeneity of results remained high, even after sensitivity analysis excluding studies of high risk of bias (not shown).

Neurologic complications that were most frequently reported were central nervous system infection and drug‐related events.

**Figure 2:**
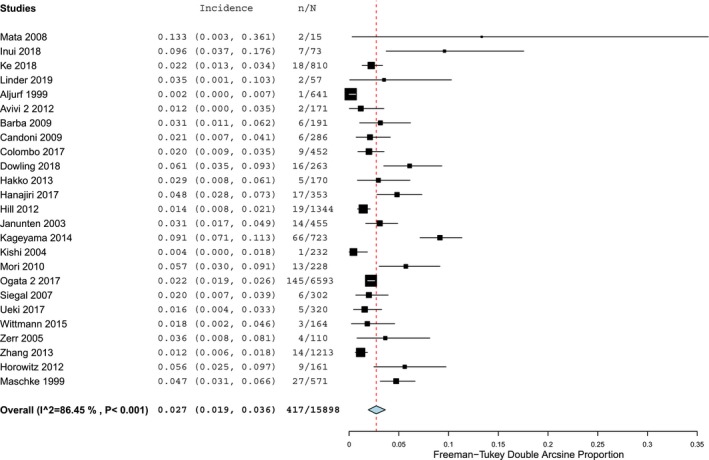
Forest plot of retrospective cohort studies reporting infectious neurological complications.

**Figure 3:**
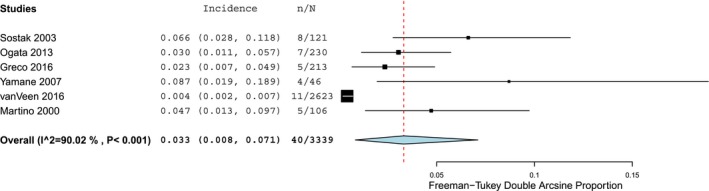
Forest plot of prospective cohort studies reporting infectious neurological complications.

As presented in the forest plots of Figures 2 and 3, infectious involvement of nervous system after allo‐HCT was present in 2.7% (95% CI 1.9–3.6), *Ι* ^2^ = 86.5% (*P* < 0.001) and in 3.3% (95% CI 0.8–7.1), *Ι* ^2^ = 90.0% (*P* < 0.001) of patients, in retrospective and prospective cohort studies, respectively.

**Figure 4:**
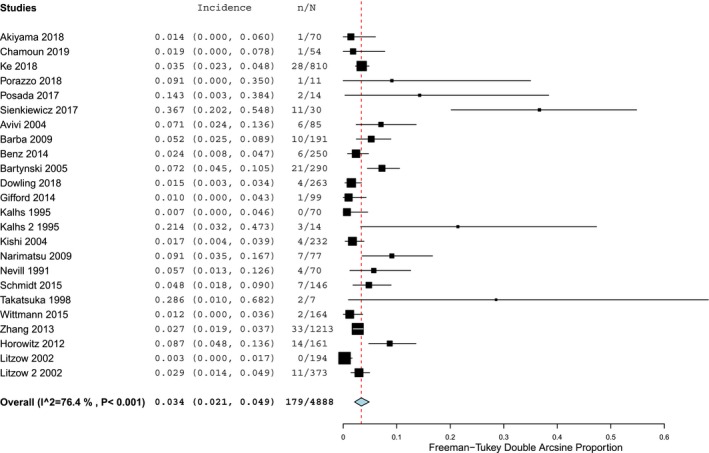
Forest plot of retrospective cohort studies reporting drug‐related neurological complications.

**Figure 5:**
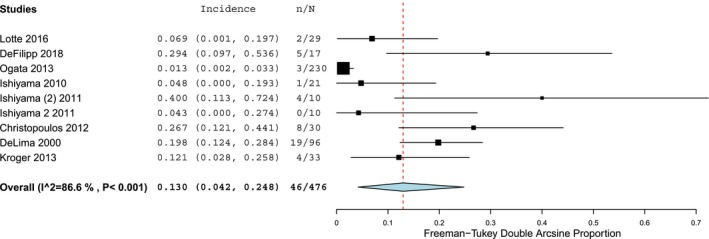
Forest plot of prospective cohort studies reporting infectious neurological complications.

Then, we examined the incidence of the second most frequently reported neurologic type of complication, drug‐related events. Overall, in retrospective studies 3.4% (95% CI 2.1–4.9), *Ι* ^2^ = 76.4% (*P* < 0.001) of the population suffered from neurologic symptoms associated with drugs used during allo‐HCT (Fig. 4). However, in prospective cohorts the equivalent incidence was higher representing 13% (95% CI 4.2–24.8), *Ι* ^2^ = 86.6% (*P* < 0.001) of the total population (Fig. 5).

The pooled rates of cerebrovascular events, TA‐TMA, immune‐mediated, relapse in CNS, and metabolic neurologic events originated from retrospective cohorts of fair risk of bias as shown in Figures S2 to S6 (online only). Immune‐mediated diseases remain a challenging diagnosis after allo‐HCT. We observed a relatively low incidence of 0.6% (95%CI 0.3‐0.9), supported by low heterogeneity *Ι* ^2^ = 36.2% (*P* < 0.001). We observed an incidence of 2.2% (95% CI 1.2–3.5), *Ι* ^2^ = 86.6% (*P* < 0.001) for cerebrovascular diseases. Many studies did not include CNS relapse of the original disease as a neurologic event after allo‐HCT. In retrospective cohorts that presented CNS relapse after allo‐HCT the resulting incidence was 2.3 % (95% CI 0.9–4.3), *Ι* ^2^ = 93.7% (*P* < 0.001). The definition of metabolic encephalopathy or complication was hardly predetermined in studies. The incidence of neurologic complications attributed to metabolic type was 2.4% (95% CI 1.1–4.1), *Ι* ^2^ = 82.5% (*P* < 0.001). Interestingly, ΤΑ‐ΤΜΑ incidence (with neurologic manifestation at diagnosis) was 3.4% (95% CI 1.7–5.6) *Ι* ^2^ = 84.9% (*P* < 0.001) suggesting that is not a rare neurologic event after allo‐HCT.

Higher cortical functions, peripheral not otherwise specified, PTLD, and other types of neurologic complications did not provide sufficient data to be part of the analysis.

### Case fatality rate

The secondary endpoint of our study was the interpretation of the impact of neurologic events in the survival of patients after allo‐HCT. We decided not to calculate any death rate as summary measure, due to high heterogeneity between studies, types of complications, and study populations. Therefore, we describe fatal events for each study type separately.

In case reports, fatal outcome was related to neurologic complications in 124 of 489 (25.4%) cases. Eight case reports did not address the outcome of the case. In 21 case series, the number of deaths attributed to neurologic complications ranged from 0 to 100% with a median of 29%. This information was not available in six studies. Regarding case–controls studies, only Conrad et al^24^ provided the number of fatal neurologic cases leading to a 60% rate (6/10) of patients with neurologic events. The majority of prospective cohorts, 11/17 studies, did not examine the outcome of patients with neurologic complications. Therefore, in the six studies with available information death due to neurologic complications ranged from 0 to 100% with a median of 25%. Finally, we could not extract the outcome of neurologic events for 804/1700 patients with neurologic symptoms in 34 retrospective cohort studies. Mortality of patients with neurologic complication in the remaining retrospective cohorts ranged from 0 to 100% with a median of 25.5%. In the one RCT included in the systematic review, one patient of the methotrexate and one of the cyclosporine group died of cerebral hemorrhage and encephalopathy, respectively, resulting in a case fatality rate of 12.5% among patients with neurologic symptoms.

### Risk of bias across studies and publication bias

Based on study type, high risk of bias in most of the included studies, as well as significant heterogeneity in results that come from observational data, high imprecision, and suspected publication bias (Egger’s test and funnel plot not shown), quality of evidence was very low applying the GRADE tool (Table S7‐online only)^15^.

## Discussion

### Summary of evidence

Our systematic review and meta‐analysis highlights and classifies the wide spectrum of neurologic complications in patients post allo‐HCT for hematologic diseases. Incidence rates varied, according to type of complications and studies, from 0.6% for immune‐mediated disorders in retrospective cohorts to 13% for drug‐related events in prospective cohorts. This can be attributed to the fact that most studies examined a selected population that received a certain treatment in a limited time period. Interestingly, the majority of these complications occurred early as well as late posttransplant rendering long‐term increased awareness of treating physicians of utmost importance. Nevertheless, no valid conclusions can be drawn taking into consideration the lack of information about the follow‐up period in many studies and the diversity of treatment regimens.

Regarding severity of neurologic complications and death rate in patients with outcome of interest, data were too diverse to provide any safe conclusion. This was mainly explained by different neurologic events (e.g., intracranial hemorrhage could not be compared with cognitive impairment in terms of mortality) as well as the different treatment regimens and study designs. Furthermore, considering the multimorbidity of allo‐HCT recipients, including multiple organ failure and disease relapse, death could rarely be attributed to neurologic events alone. Therefore, mortality rates due to neurologic complications might have been underestimated in many studies.

### Results in context of existing evidence

To our knowledge, this is, the first systematic review that addresses the chaotic subject of neurologic complications after allo‐HCT and its impact on survival. Scheurer et al^40^ carried out a systematic review investigating a subtype of neurologic episodes, HHV‐6 encephalitis in umbilical cord blood transplantation (CBT). This study included both pediatric and adult population and selected a specific type of allo‐HCT. The pooled prevalence of HHV‐6 encephalitis among patients who underwent CBT was 8.3% and 0.5% for stem cell source other than cord blood. Due to nonequivalent patient population, no comparison can be made with the incidence for infectious neurologic events.^36^

The involvement of CNS or PNS in GVHD was not proven until recently and still remains a challenging diagnosis.^41^ We present that an important number of studies addressing this issue, mostly case reports, were published during the last decade.^42^ Interestingly, in our meta‐analysis the pooled incidence of immune‐mediated disorders including GVHD was 0.6% (95% CI 0.3–0.9) with low heterogeneity *Ι* ^2^ = 36.2% (*P* < 0.001). On the contrary, TA‐TMA with neurologic dysfunction incidence was found to be high compared to other types [3.4% (95% CI 1.7–5.6) *Ι* ^2^ = 84.9% (*P* < 0.001)] in accordance with previous studies.^43^

Regarding the secondary outcome, neurologic complications are considered to be devastating events after allo‐HCT according to observational data and single‐center experiences. No systematic review has ever been conducted to support that theory. Scarce data can be found in Lin et al^44^ who studied aspergillosis (not only in CNS) and reported a case fatality rate of 86.7% in transplanted patient. Moreover, in the meta‐analysis of van de Peppel^45^ the case fatality rate for patients (who not necessarily underwent allo‐HCT) with proven invasive aspergillosis in CNS was 72%. We report a wide variety of burden of neurologic complications after allo‐HCT according to study type ranging from minor 12.5% as reported in the RCT to devastating 60% of case–control studies. However, in retrospective cohorts the outcome of 896 patients was reported and case fatality rate ranged from 0 to 100% with median 25.5%. This variability should be explained with caution due to the described heterogeneity of neurologic signs and symptoms, definition of those events and diagnostic strategy of each center, the variety of hematologic transplant procedures, study designs and the indefinable follow‐up period. It should be noted that most data in this review emerge from real‐world retrospective studies that lack appropriate methodology to reach safe conclusions. This fact not only reflects the complexity of the field but also the need for a systematic approach in future studies.

### Strengths and limitations of study

Our study incorporated a large amount of information about different types of neurologic complications in allo‐HCT recipients coming from studies of inferior quality. This can be considered strength of the present systematic review as we tried to be as inclusive as possible, following a comprehensive search strategy and accepting all study types despite the limitation of increased heterogeneity. We provided therefore a systematic review of all available information on the subject.

However, our study has several limitations translated into different sources of bias. First, our systematic review incorporated studies that included patients with disease relapse posttransplant in an effort to describe the broad spectrum of neurologic adverse events, in spite of the presence of CNS disease pre and posttransplant. Second, we studied only papers in English introducing possible selection bias. Moreover, our meta‐analysis resulted in noticeable heterogeneity even after subgroup and sensitivity analysis. However, this was expected taking into consideration the lack of specific guidelines for the definition of neurologic complications after HCT and the variability of transplantation procedures. Finally, in our review we decided to assess publication bias with usual methods, acknowledging, however, their limitations in this particular occasion of one arm studies.^21^ This resulted in detection of probable publication bias which may be overestimated in our study.

### Implications

The aim of this systematic review was to provide all existing evidence in the literature in the form of the highest level of the pyramid of evidence‐based medicine, to clinical physicians. For the consultant neurologist with a specific interest in this field, it is important to maintain a high index of suspicion in certain subgroups of patients. Thus, long‐term increased awareness even during the late posttransplant period and collaboration between expert physicians is necessary to improve patient outcomes.

Further research with methodologically well‐organized studies is likely to establish solid recommendations about the incidence, type, and treatment of neurologic complications after HCT.

### Conclusions

In conclusion, various types of neurologic complications can be confronted in adult patients after allo‐HCT even as late events. This systematic review summarizes existing data and provides the necessary background information for every physician involved in the management of these patients. The neurologic incidence varies a lot between treatment regimens and types of neurologic complications and treating hematologists should bear in mind the broadness of the subject for best interest of this special group of patients.

## Author Contribution

MG and EG screened the studies and extracted the data in mutual agreement. MG performed the data collection and wrote the first draft of the manuscript. MM, MG, and EG collaborated for the design of the study. MG in collaboration with MM and A‐BH performed analyses. VK, IS, SP, and AA participated in study conception and design and edited the manuscript.

## Conflict of Interest

Nothing to declare.

## Supporting information


**Data S1**. Methods.
**Figure S1**. Analysis of all studies included in quantitative synthesis.
**Figure S2**. Analysis of studies reporting immune‐mediated neurological complications.
**Figure S3**. Analysis of studies reporting cerebrovascular disorders.
**Figure S4**. Analysis of studies reporting central nervous system relapse.
**Figure S5**. Analysis of studies reporting metabolic disorders manifestated as neurological complications.
**Figure S6**. Analysis of studies reporting transplant‐associated thrombotic microangiopathy.
**Table S1**. PRISMA 2009 Checklist.
**Table S2**. Search strategy.
**Table S3**. Case report studies’ characteristics.
**Table S4**. Prospective cohorts, randomized control trial (RCT) and case–control studies characteristics.
**Table S5**. Retrospective cohort studies characteristics.
**Table S6**. Case series studies characteristics.
**Table S7**. GRADE tool.

## References

[1] Passweg JR , Baldomero H , Peters C , et al. Hematopoietic SCT in Europe: data and trends in 2012 with special consideration of pediatric transplantation. Bone Marrow Transplant 2014;49:744–750. 24637898 10.1038/bmt.2014.55 PMC4051369

[2] Horan JT , Logan BR , Agovi‐Johnson MA , et al. Reducing the risk for transplantation‐related mortality after allogeneic hematopoietic cell transplantation: how much progress has been made? J Clin Oncol 2011;29:805–813. 21220593 10.1200/JCO.2010.32.5001 PMC3068057

[3] Tauro S , Craddock C , Peggs K , et al. Allogeneic stem‐cell transplantation using a reduced‐intensity conditioning regimen has the capacity to produce durable remissions and long‐term disease‐free survival in patients with high‐risk acute myeloid leukemia and myelodysplasia. J Clin Oncol 2005;23:9387–9393. 16314618 10.1200/JCO.2005.02.0057

[4] Siegal D , Keller A , Xu W , et al. Central nervous system complications after allogeneic hematopoietic stem cell transplantation: incidence, manifestations, and clinical significance. Biol Blood Marrow Transplant 2007;13:1369–1379. 17950923 10.1016/j.bbmt.2007.07.013

[5] Maffini E , Festuccia M , Brunello L , et al. Neurologic Complications after Allogeneic Hematopoietic Stem Cell Transplantation. Biol Blood Marrow Transplant 2017;23:388–397. 28039081 10.1016/j.bbmt.2016.12.632

[6] Sakellari I , Gavriilaki E , Papagiannopoulos S , et al. Neurological adverse events post allogeneic hematopoietic cell transplantation: major determinants of morbidity and mortality. J Neurol 2019;266:1960–1972. 31087160 10.1007/s00415-019-09372-3

[7] Pruitt AA , Graus F , Rosenfeld MR . Neurological complications of transplantation: part I: hematopoietic cell transplantation. Neurohospitalist 2013;3:24–38. 23983885 10.1177/1941874412455338 PMC3726122

[8] Zhang XH , Wang QM , Chen H , et al. Clinical characteristics and risk factors of Intracranial hemorrhage in patients following allogeneic hematopoietic stem cell transplantation. Ann Hematol 2016;95:1637–1643. 27485455 10.1007/s00277-016-2767-y

[9] Turhal NS . Cyclosporin A and imipenem associated seizure activity in allogeneic bone marrow transplantation patients. J Chemother 1999;11:410–413. 10632390 10.1179/joc.1999.11.5.410

[10] Liberati A , Altman DG , Tetzlaff J , et al. The PRISMA statement for reporting systematic reviews and meta‐analyses of studies that evaluate healthcare interventions: explanation and elaboration. BMJ 2009;339:b2700. 19622552 10.1136/bmj.b2700 PMC2714672

[11] Coccia PF , Pappo AS , Beaupin L , et al. Adolescent and Young Adult Oncology, Version 2.2018, NCCN Clinical Practice Guidelines in Oncology. J Natl Compr Canc Netw 2018;16:66–97. 29295883 10.6004/jnccn.2018.0001

[12] Murad MH , Sultan S , Haffar S , Bazerbachi F . Methodological quality and synthesis of case series and case reports. BMJ Evid‐Based Med 2018;23:60–3. 10.1136/bmjebm-2017-110853 PMC6234235 29420178

[13] Cook DA , Reed DA . Appraising the quality of medical education research methods: the Medical Education Research Study Quality Instrument and the Newcastle‐Ottawa Scale‐Education. Acad Med 2015;90:1067–1076. 26107881 10.1097/ACM.0000000000000786

[14] Higgins JP , Altman DG . Assessing risk of bias in included studies. Cochrane Handbook for Systematic Reviews of Interventions.2008.

[15] Guyatt GH , Oxman AD , Vist GE , et al. GRADE: an emerging consensus on rating quality of evidence and strength of recommendations. BMJ 2008;336:924–926. 18436948 10.1136/bmj.39489.470347.AD PMC2335261

[16] Freeman MFT , Tukey JW . Transformations related to the angular and the square root. Ann Math Stat 1950;21:607–611.

[17] Michael Borenstein LVH , Higgins JPT , Rothstein HR . Introduction to Meta‐Analysis. Chichester: John Wiley & Sons, 2009.

[18] Higgins JG , Green S . Identifying and measuring heterogeneity. Cochrane Handbook for Systematic. Reviews of Interventions. 2008;277.

[19] Egger M , Davey Smith G , Schneider M , Minder C . Bias in meta‐analysis detected by a simple, graphical test. BMJ 1997;315:629–634. 9310563 10.1136/bmj.315.7109.629 PMC2127453

[20] Egger M , Smith GD , Schneider M , Minder C . Bias in meta‐analysis detected by a simple, graphical test. BMJ 1997;315:629–634. 9310563 10.1136/bmj.315.7109.629 PMC2127453

[21] Hunter JP , Saratzis A , Sutton AJ , et al. In meta‐analyses of proportion studies, funnel plots were found to be an inaccurate method of assessing publication bias. J Clin Epidemiol 2014;67:897–903. 24794697 10.1016/j.jclinepi.2014.03.003

[22] Colombo AA , Marchioni E , Diamanti L , et al. Neurological complications involving the central nervous system after allogeneic hematopoietic stem cell transplantation during a period of evolution in transplant modalities: a cohort analysis. Transplantation 2017;101:616–623. 27222935 10.1097/TP.0000000000001257

[23] Irle C , Deeg HJ , Buckner CD , et al. Marrow transplantation for leukemia following fractionated total body irradiation. A comparative trial of methotrexate and cyclosporine. Leuk Res 1985;9:1255–1261. 3906282 10.1016/0145-2126(85)90153-5

[24] Conrad A , Le Marechal M , Dupont D , et al. A matched case‐control study of toxoplasmosis after allogeneic haematopoietic stem cell transplantation: still a devastating complication. Clin Microbiol Infect 2016;22:636–641. 27172809 10.1016/j.cmi.2016.04.025

[25] Daly AS , Hasegawa WS , Lipton JH , et al. Transplantation‐associated thrombotic microangiopathy is associated with transplantation from unrelated donors, acute graft‐versus‐host disease and venoocclusive disease of the liver. Transfus Apher Sci 2002;27:3–12. 12201469 10.1016/s1473-0502(02)00020-4

[26] Ishiyama K , Katagiri T , Ohata K , et al. Safety of pre‐engraftment prophylactic foscarnet administration after allogeneic stem cell transplantation. Transpl Infect Dis 2012;14:33–39. 21794043 10.1111/j.1399-3062.2011.00662.x

[27] Ishiyama K , Katagiri T , Hoshino T , et al. Preemptive therapy of human herpesvirus‐6 encephalitis with foscarnet sodium for high‐risk patients after hematopoietic SCT. Bone Marrow Transplant 2011;46:863–869. 20838386 10.1038/bmt.2010.201

[28] de Lima M , Couriel D , Thall PF , et al. Once‐daily intravenous busulfan and fludarabine: clinical and pharmacokinetic results of a myeloablative, reduced‐toxicity conditioning regimen for allogeneic stem cell transplantation in AML and MDS. Blood 2004;104:857–864. 15073038 10.1182/blood-2004-02-0414

[29] Christopoulos P , Bertz H , Ihorst G , et al. Radiation‐free allogeneic conditioning with fludarabine, carmustine, and thiotepa for acute lymphoblastic leukemia and other hematologic malignancies necessitating enhanced central nervous system activity. Biol Blood Marrow Transplant 2012;18:1430–1437. 22430085 10.1016/j.bbmt.2012.02.016

[30] DeFilipp Z , Li S , Kempner ME , et al. Phase I trial of brentuximab vedotin for steroid‐refractory chronic graft‐versus‐host disease after allogeneic hematopoietic cell transplantation. Biol Blood Marrow Transplant 2018;24:1836–1840. 29758393 10.1016/j.bbmt.2018.05.012

[31] Kroger N , Zabelina T , Klyuchnikov E , et al. Toxicity‐reduced, myeloablative allograft followed by lenalidomide maintenance as salvage therapy for refractory/relapsed myeloma patients. Bone Marrow Transplant 2013;48:403–407. 22863722 10.1038/bmt.2012.142

[32] van der Wagen LE , Lt Boome , Nijhof I , et al. Effective treatment of severe chronic graft versus host disease with a combination of B‐Cell depletion and tyrosine kinase inhibition. Blood 2016;128:4565.

[33] Koeppen S , Thirugnanasambanthan A , Koldehoff M . Neuromuscular complications after hematopoietic stem cell transplantation. Support Care Cancer 2014;22:2337–2341. 24682581 10.1007/s00520-014-2225-0

[34] Beglinger LJ , Mills JA , Vik SM , et al. The neuropsychological course of acute delirium in adult hematopoietic stem cell transplantation patients. Arch Clin Neuropsychol 2011;26:98–109. 21183605 10.1093/arclin/acq103 PMC3104596

[35] Scherwath A , Schirmer L , Kruse M , et al. Cognitive functioning in allogeneic hematopoietic stem cell transplantation recipients and its medical correlates: a prospective multicenter study. Psycho‐oncology 2013;22:1509–1516. 22945857 10.1002/pon.3159

[36] Sostak P , Padovan CS , Yousry TA , et al. Prospective evaluation of neurological complications after allogeneic bone marrow transplantation. Neurology 2003;60:842–848. 12629244 10.1212/01.wnl.0000046522.38465.79

[37] Goto T , Tanaka T , Sawa M , et al. Prospective observational study on the first 51 cases of peripheral blood stem cell transplantation from unrelated donors in Japan. Int J Hematol 2018;107:211–221. 29027623 10.1007/s12185-017-2341-y

[38] Stefanou MI , Bischof F . Central and peripheral nervous system immune‐mediated demyelinating disease after allogeneic hematopoietic stem cell transplantation. J Neuroimmunol 2017;307:74–81. 28495143 10.1016/j.jneuroim.2017.04.005

[39] Grauer O , Wolff D , Bertz H , et al. Neurological manifestations of chronic graft‐versus‐host disease after allogeneic haematopoietic stem cell transplantation: report from the Consensus Conference on Clinical Practice in chronic graft‐versus‐host disease. Brain 2010;133:2852–2865. 20846944 10.1093/brain/awq245

[40] Scheurer ME , Pritchett JC , Amirian ES , et al. HHV‐6 encephalitis in umbilical cord blood transplantation: a systematic review and meta‐analysis. Bone Marrow Transplant 2013;48:574–580. 23000642 10.1038/bmt.2012.180

[41] Hartrampf S , Dudakov JA , Johnson LK , et al. The central nervous system is a target of acute graft versus host disease in mice. Blood 2013;121:1906–1910. 23299314 10.1182/blood-2012-09-456590 PMC3591808

[42] Polchlopek Blasiak K , Simonetta F , Vargas MI , Chalandon Y . Central nervous system graft‐versus‐host disease (CNS‐GvHD) after allogeneic haematopoietic stem cell transplantation. BMJ Case Rep 2018;2018. 10.1136/bcr-2017-221840 PMC5780588 29330269

[43] Cho BS , Yahng SA , Lee SE , et al. Validation of recently proposed consensus criteria for thrombotic microangiopathy after allogeneic hematopoietic stem‐cell transplantation. Transplantation 2010;90:918–926. 20717073 10.1097/TP.0b013e3181f24e8d

[44] Lin SJ , Schranz J , Teutsch SM . Aspergillosis case‐fatality rate: systematic review of the literature. Clin Infect Dis 2001;32:358–366. 11170942 10.1086/318483

[45] van de Peppel RJ , Visser LG , Dekkers OM , de Boer MGJ . The burden of Invasive Aspergillosis in patients with haematological malignancy: a meta‐analysis and systematic review. J Infect 2018;76:550–562. 29727605 10.1016/j.jinf.2018.02.012

