# Supplementary material for: Neurologic complications after allogeneic transplantation: a meta‐analysis
Source: Ann Clin Transl Neurol. 2019 Sep 27;6(10):2037–47. doi: 10.1002/acn3.50909 (PMC6801165; doi:10.1002/acn3.50909)
Supplement: Supplementary file 1 — Data S1. Methods. Figure S1. Analysis of all studies included in quantitative synthesis. Figure S2. Analysis of studies reporting immune‐mediated neurological complications. Figure S3. Analysis of studies reporting cerebrovascular disorders. Figure S4. Analysis of studies reporting central nervous system relapse. Figure S5. Analysis of studies reporting metabolic disorders manifestated as neurological complications. Figure S6. Analysis of studies reporting transplant‐associated thrombotic microangiopathy. Table S1. PRISMA 2009 Checklist. Table S2. Search strategy. Table S3. Case report studies’ characteristics. Table S4. Prospective cohorts, randomized control trial (RCT) and case–control studies characteristics. Table S5. Retrospective cohort studies characteristics. Table S6. Case series studies characteristics. Table S7. GRADE tool. [file ACN3-6-2037-s001.pdf]

Supplement part I

Methods

Search

We identified additional candidate studies by reviewing references of included studies. Taking into account that we concluded to a number of abstracts from American society of Hematology (ASH) and European Bone Marrow Transplantation (EBMT) meetings to be reckoned with we decided not to further search grey literature through other large conferences.

Eligibility criteria

We deemed eligible all types of primary studies including randomized controlled trials (RCTs), controlled observational studies (both cohort and case control), case series and case reports in adult patients who underwent allo-HCT for hematologic diseases and experienced a neurologic disorder after transplant. We excluded pediatric population studies (patients ≤14 years old) in order to minimize heterogeneity(1). The form of the report was not an exclusion criterion in order to achieve a holistic approach to our primary endpoint. Therefore, we included case reports/case series and non-original articles such as abstracts and posters from meetings or letters.

Data collection process

A second reviewer checked the gathered data from extraction for accuracy and filled the gaps by referring to the full-text of the study. For studies with more than one

publication record, we extracted data from the record with the longest follow-up period and the more detailed description of the outcome of interest.

Risk of bias in individual studies

We followed previous publications in which cohort studies enabled the calculation of a rate for the outcome; whereas case series were mainly descriptive of selected/random cases(13, 14). There was a difficulty in distinguishing retrospective cohort studies that describe mostly the experience of a center and do not report some kind of comparison, from case series(2). This is a well-recognized problem in the literature.

As a case report we considered a study that included ≤4 patients based on previous literature reviews(15). We excluded case reports from further evaluation, such as the assessment of risk of bias, and presented them descriptively in the results and thoroughly in supplementary table 3.

Type of neurologic event

Neurologic complications were grouped according to the time of presentation of neurologic symptoms after transplantation as early post-transplantation, if they were detected until 100 days after the procedure or late post-transplantation when their time of onset was after 101 days. Another classification was according to the type of complication into the following: drug-related (when it was clearly stated so by the reporters), metabolic (concerning mostly Wernicke encephalopathy), infectious (viral, fungal, protozoan, bacterial), cerebrovascular (stroke, cerebral venous thrombosis or hemorrhagic event), immune-mediated [myasthenia gravis,

Guillain-Barré, myositis, demyelinating diseases, chronic graft-versus-host disease (cGHVD), transverse myelitis], relapse of disease in either PNS or CNS, peripheral (if no alternative explanation such as drug-related or cGVHD for PNS signs was given), higher cortical functions/cognitive impairment, post-transplantation lymphoproliferative disorders, transplantation-associated thrombotic microangiopathy (TA-TMA) presented with neurologic phenotype and other when none of the above type was able to depict it (for example posterior reversible encephalopathy syndrome (PRES) not otherwise explained, thrombotic thrombocytopenic purpura)(5). Regarding drug-related complications, we carefully selected only those drugs that were used in allo-HCT procedure or in GVHD therapy.

Summary measures and synthesis of results

Case reports and case series were excluded from meta-analysis since it was not possible to estimate any incidence from the reported data. RCTs and case control studies were very few to provide adequate data for analysis and were not mixed with cohort studies due to great differences in design. In the rare case that a cohort study had two cohorts with different event rate of neurologic complications we included it as two studies. In any case that the proportion (n/N) could not be estimated safely (for example the age of the N was not available), the study was excluded from the analysis. We expected high heterogeneity in our results, due to variety of neurologic complications and diverse population of included studies. When I^2 exceeded 75% the heterogeneity was considered high (8).

Sensitivity and subgroup analyses

The number of studies included in sensitivity or subgroup analysis should be five or more in order to draw a reliable conclusion(12).

1. Smith AW, Seibel NL, Lewis DR, Albritton KH, Blair DF, Blanke CD, et al. Next steps for adolescent and young adult oncology workshop: An update on progress and recommendations for the future. Cancer. 2016 Apr 1;122(7):988-99.
2. Mathes T, Pieper D. Clarifying the distinction between case series and cohort studies in systematic reviews of comparative studies: potential impact on body of evidence and workload. BMC medical research methodology. 2017 Jul 17;17(1):107.
3. Murad MH, Sultan S, Haffar S, Bazerbachi F. Methodological quality and synthesis of case series and case reports. BMJ evidence-based medicine. 2018 Apr;23(2):60-3.
4. Cook DA, Reed DA. Appraising the quality of medical education research methods: the Medical Education Research Study Quality Instrument and the Newcastle-Ottawa Scale-Education. Academic medicine : journal of the Association of American Medical Colleges. 2015 Aug;90(8):1067-76.
5. Maffini E, Festuccia M, Brunello L, Boccadoro M, Giaccone L, Bruno B. Neurologic Complications after Allogeneic Hematopoietic Stem Cell Transplantation. Biology of blood and marrow transplantation : journal of the American Society for Blood and Marrow Transplantation. 2017 Mar;23(3):388-97.
6. Michael Borenstein LVH, J. P. T. Higgins and H. R. Rothstein. Introduction to Meta-Analysis. John Wiley & Sons, 2009.
7. Freeman MFT, J.W. Transformations Related to the Angular and the Square Root. The Annals of Mathematical Statistics 1950;21(4).
8. Higgins JG, S. Identifying and measuring heterogeneity. Cochrane Handbook for Systematic Reviews of Interventions. 2008:277.
9. Egger M, Davey Smith G, Schneider M, Minder C. Bias in meta-analysis detected by a simple, graphical test. Bmj. 1997 Sep 13;315(7109):629-34.
10. Egger M, Smith GD, Schneider M, Minder C. Bias in meta-analysis detected by a simple, graphical test. BMJ. 1997;315(7109):629-34.
11. Hunter JP, Saratzis A, Sutton AJ, Boucher RH, Sayers RD, Bown MJ. In meta-analyses of proportion studies, funnel plots were found to be an inaccurate method of assessing publication bias. Journal of clinical epidemiology. 2014 Aug;67(8):897-903.
12. Guolo A, Varin C. Random-effects meta-analysis: the number of studies matters. Statistical methods in medical research. 2017 Jun;26(3):1500-18.

Supplementary figures

Studies	Incidence	n//N
Lotte 2016	0.069 (0.001, 0.197)	2/29
Goto 2018	0.020 (0.000, 0.082)	1/51
DeFilipp 2018	0.294 (0.097, 0.536)	5/17
Sostak 2003	0.380 (0.295, 0.469)	46/121
Koeppen 2014	0.047 (0.028, 0.069)	20/429
Ogata 2013	0.143 (0.101, 0.192)	33/230
Greco 2016	0.023 (0.007, 0.049)	5/213
Yamane 2007	0.087 (0.019, 0.189)	4/46
Beglinger 2011	0.364 (0.173, 0.578)	8/22
Ishiyama 2010	0.048 (0.000, 0.193)	1/21
Ishiyama (2) 2011	0.400 (0.113, 0.724)	4/10
Ishiyama 2 2011	0.043 (0.000, 0.274)	0/10
vanVeen 2016	0.004 (0.002, 0.007)	11/2623
Christopoulos 2012	0.267 (0.121, 0.441)	8/30
DeLima 2000	0.198 (0.124, 0.284)	19/96
Martino 2000	0.047 (0.013, 0.097)	5/106
Kroger 2013	0.121 (0.028, 0.258)	4/33
Mata 2008	0.133 (0.003, 0.361)	2/15
Seto 2018	0.017 (0.004, 0.039)	4/230
Akiyama 2018	0.014 (0.000, 0.060)	1/70
Bhamidipati 2017	0.016 (0.009, 0.025)	15/940
Chamoun 2019	0.019 (0.000, 0.078)	1/54
dePhilippis 2018	0.037 (0.000, 0.152)	1/27
Gavriilaki 2018	0.034 (0.022, 0.049)	26/758
Hirano 2017	0.065 (0.008, 0.159)	3/46
Inui 2018	0.096 (0.037, 0.176)	7/73
Ke 2018	0.133 (0.111, 0.158)	108/810
Linder 2019	0.035 (0.001, 0.103)	2/57
Porazzo 2018	0.091 (0.000, 0.350)	1/11
Posada 2017	0.143 (0.003, 0.384)	2/14
Scordo 2018	0.084 (0.042, 0.138)	11/131
Sienkiewicz 2017	0.367 (0.202, 0.548)	11/30
Ting−An 2019	0.044 (0.027, 0.064)	20/459
Famoso 2019	0.018 (0.000, 0.118)	0/26
Famoso 2 2019	0.188 (0.068, 0.344)	6/32
Aljurf 1999	0.002 (0.000, 0.007)	1/641
Aoki 2014	0.002 (0.001, 0.003)	10/5068
Avivi 2004	0.071 (0.024, 0.136)	6/85
Avivi 2 2012	0.012 (0.000, 0.035)	2/171
Barba 2009	0.141 (0.095, 0.195)	27/191
Benz 2014	0.024 (0.008, 0.047)	6/250
Bartynski 2005	0.072 (0.045, 0.105)	21/290
Candoni 2009	0.021 (0.007, 0.041)	6/286
Cho 2008	0.047 (0.018, 0.088)	7/148
Colombo 2017	0.066 (0.045, 0.091)	30/452
Davies 2006	0.080 (0.002, 0.226)	2/25
Delios 2012	0.005 (0.002, 0.009)	7/1484
Dowling 2018	0.281 (0.229, 0.337)	74/263
Eiten 2015	0.020 (0.004, 0.045)	4/200
Gifford 2014	0.111 (0.056, 0.181)	11/99
Goker 2015	0.115 (0.065, 0.175)	15/131
Hakko 2013	0.100 (0.059, 0.150)	17/170
Hamdi 2014	0.039 (0.023, 0.059)	18/457
Hanajiri 2017	0.048 (0.028, 0.073)	17/353
Hill 2012	0.014 (0.008, 0.021)	19/1344
Janunten 2003	0.031 (0.017, 0.049)	14/455
Kageyama 2014	0.189 (0.162, 0.219)	137/723
Kalhs 1995	0.007 (0.000, 0.046)	0/70
Kalhs 2 1995	0.214 (0.032, 0.473)	3/14
Kishi 2004	0.078 (0.046, 0.116)	18/232
Labrador 2015	0.024 (0.012, 0.040)	12/491
Mori 2010	0.057 (0.030, 0.091)	13/228
Nakase 2006	0.250 (0.008, 0.615)	2/8
Narimatsu 2009	0.130 (0.063, 0.215)	10/77
Nevill 1991	0.057 (0.013, 0.126)	4/70
Ogata 2 2017	0.022 (0.019, 0.026)	145/6593
Omer 2011	0.192 (0.145, 0.243)	48/250
Oran 2007	0.023 (0.015, 0.032)	28/1219
Oshima 2008	0.024 (0.016, 0.033)	29/1226
Sanz 2014	0.014 (0.003, 0.031)	4/288
Schmidt 2015	0.048 (0.018, 0.090)	7/146
Siegal 2007	0.245 (0.198, 0.295)	74/302
Takatsuka 1998	0.286 (0.010, 0.682)	2/7
Ueki 2017	0.016 (0.004, 0.033)	5/320
Wittmann 2015	0.073 (0.038, 0.119)	12/164
Zerr 2005	0.036 (0.008, 0.081)	4/110
Zhang 2013	0.042 (0.031, 0.054)	51/1213
Horowitz 2012	0.304 (0.235, 0.378)	49/161
Litzow 2002	0.003 (0.000, 0.017)	0/194
Litzow 2 2002	0.029 (0.014, 0.049)	11/373
Oda 2009	0.005 (0.002, 0.008)	9/1967
Maschke 1999	0.047 (0.031, 0.066)	27/571
**Overall (I^2=96.07 % , P< 0.001)**	**0.062 (0.048, 0.077)**	**1415/37450**

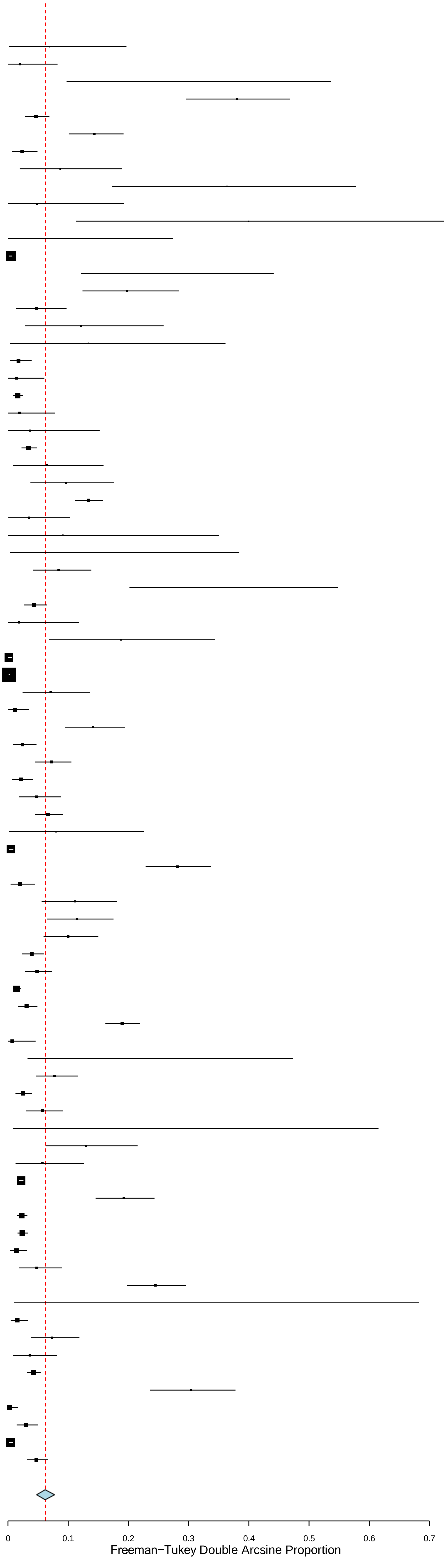

Suppl. Figure 1. Analysis of all studies included in quantitative synthesis

Supplementary figures

Suppl. Figure 2. Analysis of studies reporting immune-mediated neurological complications

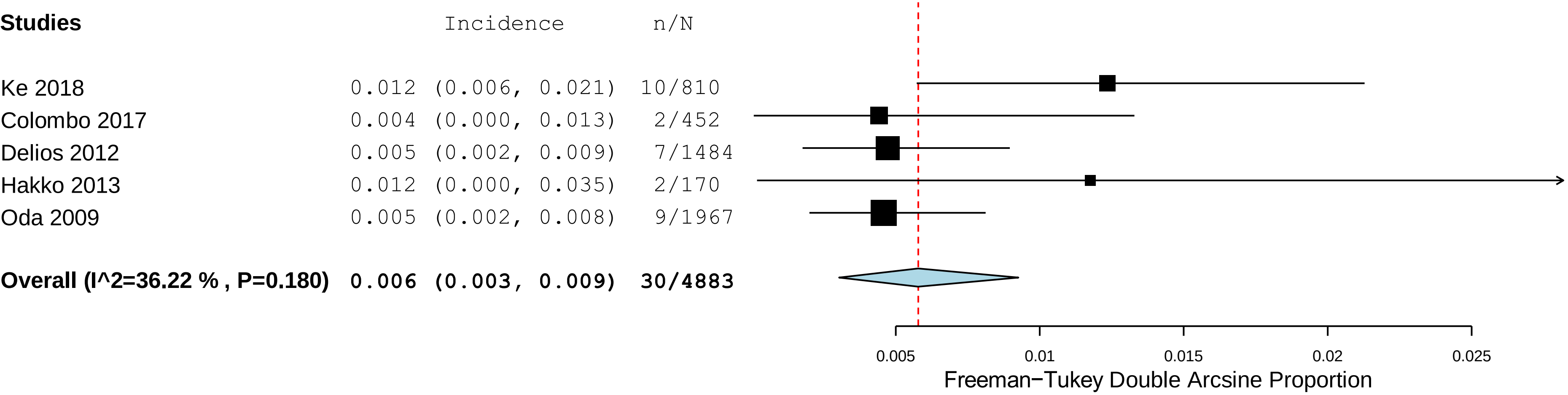

Supplementary figures

Suppl. Figure 3. Analysis of studies reporting cerebrovascular disorders

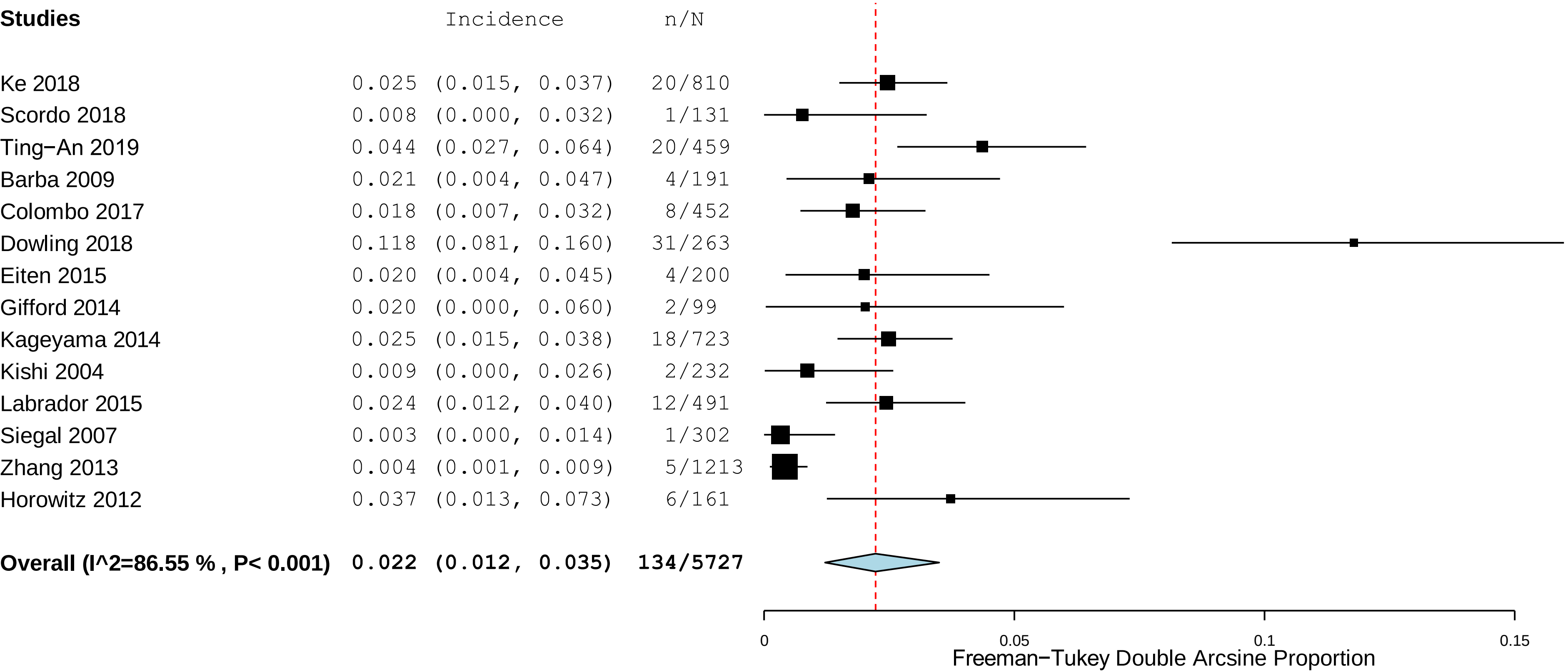

Supplementary figures

Suppl. Figure 4. Analysis of studies reporting central nervous system relapse

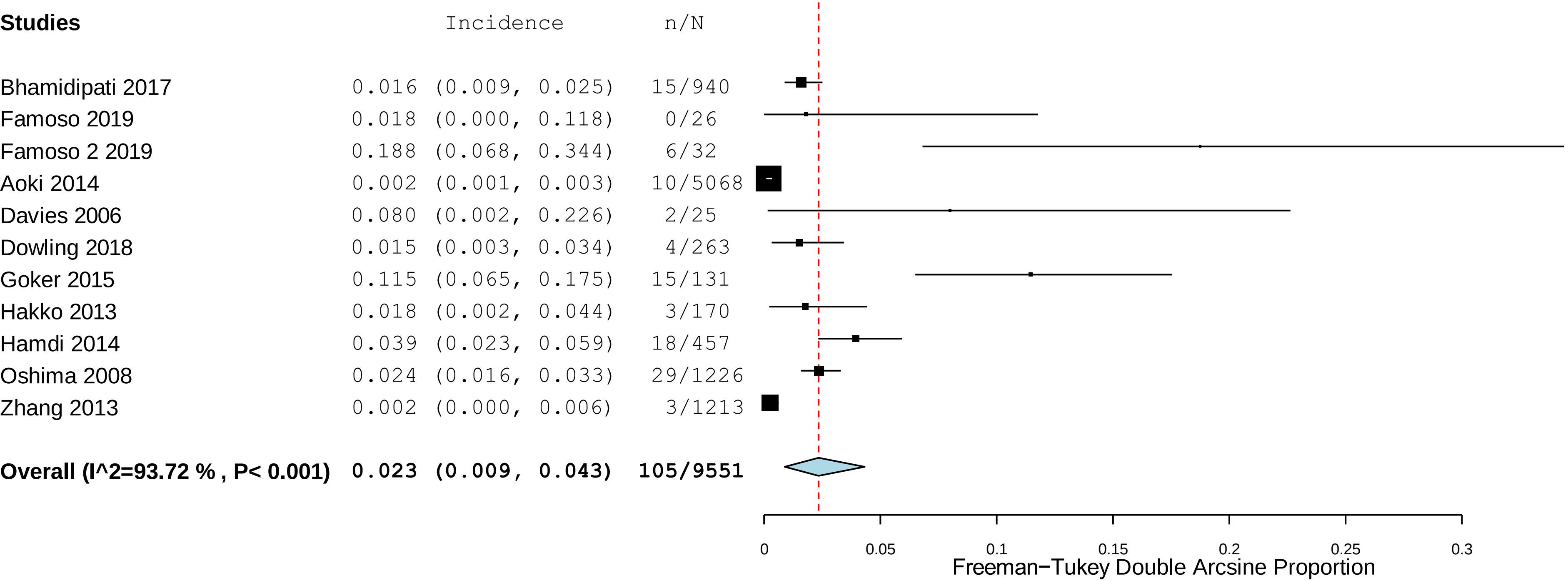

Supplementary figures

Suppl. Figure 5. Analysis of studies reporting metabolic disorders manifestated as neurological complications

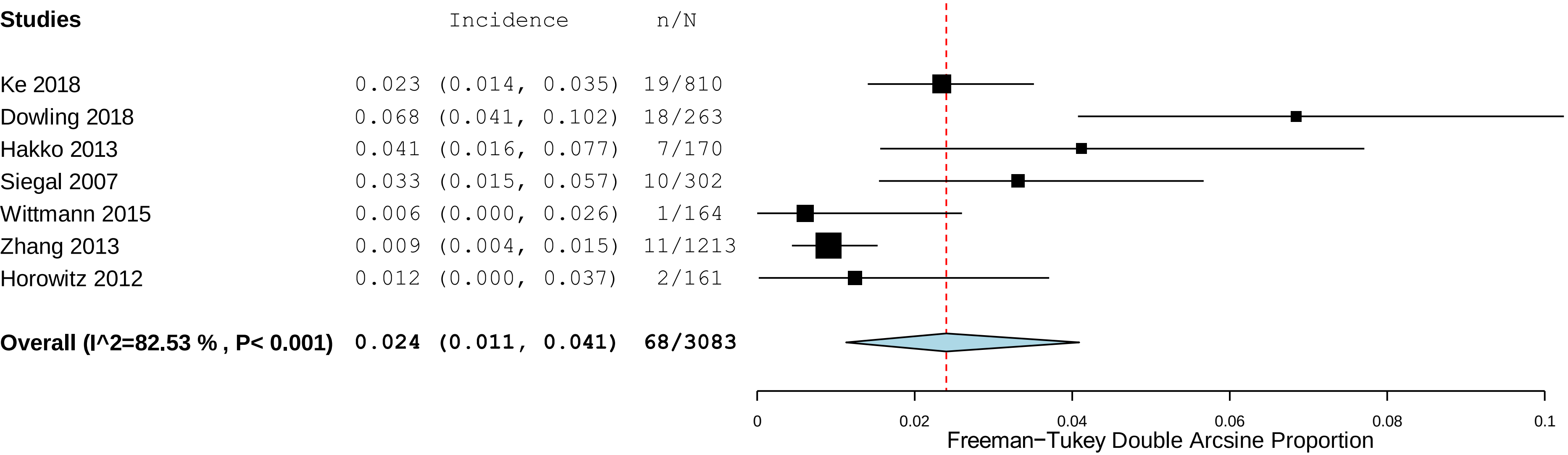

Supplementary figures

Suppl. Figure 6. Analysis of studies reporting transplant-associated thrombotic microangiopathy

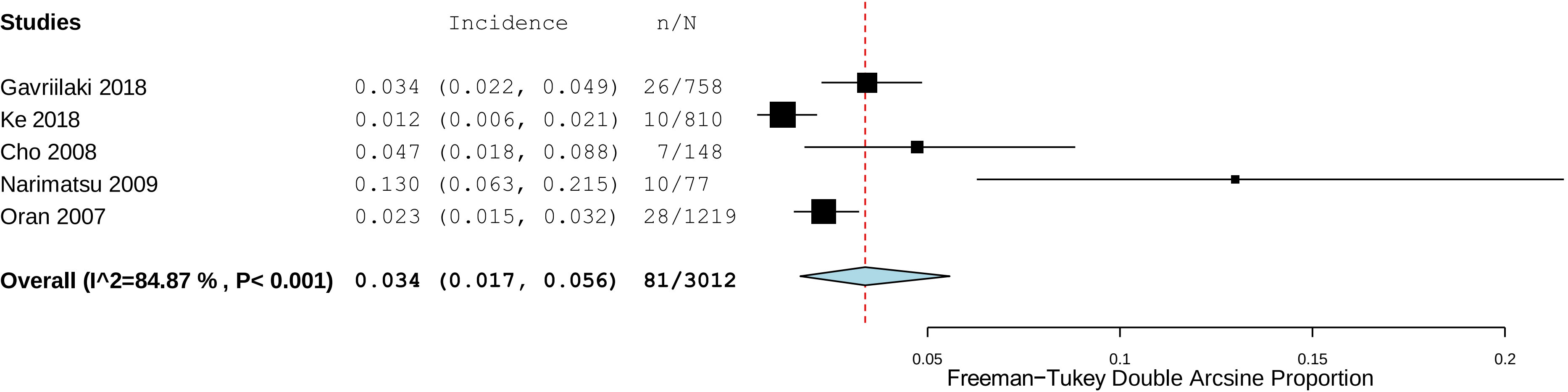

Supplementary table 1. PRISMA 2009 Checklist

Section/topic	#	Checklist item	Reported on page #
**TITLE**			
Title	1	Identify the report as a systematic review, meta-analysis, or both.	1
**ABSTRACT**			
Structured summary	2	Provide a structured summary including, as applicable: background; objectives; data sources; study eligibility criteria, participants, and interventions; study appraisal and synthesis methods; results; limitations; conclusions and implications of key findings; systematic review registration number.	3
**INTRODUCTION**			
Rationale	3	Describe the rationale for the review in the context of what is already known.	4
Objectives	4	Provide an explicit statement of questions being addressed with reference to participants, interventions, comparisons, outcomes, and study design (PICOS).	5
**METHODS**			
Protocol and registration	5	Indicate if a review protocol exists, if and where it can be accessed (e.g., Web address), and, if available, provide registration information including registration number.	-
Eligibility criteria	6	Specify study characteristics (e.g., PICOS, length of follow-up) and report characteristics (e.g., years considered, language, publication status) used as criteria for eligibility, giving rationale.	6
Information sources	7	Describe all information sources (e.g., databases with dates of coverage, contact with study authors to identify additional studies) in the search and date last searched.	5-6
Search	8	Present full electronic search strategy for at least one database, including any limits used, such that it could be repeated.	supplementary table 2
Study selection	9	State the process for selecting studies (i.e., screening, eligibility, included in systematic review, and, if applicable, included in the meta- analysis).	6
Data collection process	10	Describe method of data extraction from reports (e.g., piloted forms, independently, in duplicate) and any processes for obtaining and confirming data from investigators.	6-7
Data items	11	List and define all variables for which data were sought (e.g., PICOS, funding sources) and any assumptions and simplifications made.	7
Risk of bias in individual studies	12	Describe methods used for assessing risk of bias of individual studies (including specification of whether this was done at the study or outcome level), and how this information is to be used in any data synthesis.	7-8
Summary measures	13	State the principal summary measures (e.g., risk ratio, difference in means).	9-11
Synthesis of results	14	Describe the methods of handling data and combining results of studies, if done, including measures of consistency (e.g., I^2) for each meta- analysis.	10

Supplementary table 1. PRISMA 2009 Checklist

Risk of bias across studies	15	Specify any assessment of risk of bias that may affect the cumulative evidence (e.g., publication bias, selective reporting within studies).	10
Additional analyses	16	Describe methods of additional analyses (e.g., sensitivity or subgroup analyses, meta-regression), if done, indicating which were pre-specified.	11
**RESULTS**			
Study selection	17	Give numbers of studies screened, assessed for eligibility, and included in the review, with reasons for exclusions at each stage, ideally with a flow diagram.	11
Study characteristics	18	For each study, present characteristics for which data were extracted (e.g., study size, PICOS, follow-up period) and provide the citations.	12-15
Risk of bias within studies	19	Present data on risk of bias of each study and, if available, any outcome level assessment (see item 12).	15-16
Results of individual studies	20	For all outcomes considered (benefits or harms), present, for each study: (a) simple summary data for each intervention group (b) effect estimates and confidence intervals, ideally with a forest plot.	17-19
Synthesis of results	21	Present results of each meta-analysis done, including confidence intervals and measures of consistency.	17-19
Risk of bias across studies	22	Present results of any assessment of risk of bias across studies (see Item 15).	20
Additional analysis	23	Give results of additional analyses, if done (e.g., sensitivity or subgroup analyses, meta-regression [see Item 16]).	19-20
**DISCUSSION**			
Summary of evidence	24	Summarize the main findings including the strength of evidence for each main outcome; consider their relevance to key groups (e.g., healthcare providers, users, and policy makers).	20-21
Limitations	25	Discuss limitations at study and outcome level (e.g., risk of bias), and at review-level (e.g., incomplete retrieval of identified research, reporting bias).	22-23
Conclusions	26	Provide a general interpretation of the results in the context of other evidence, and implications for future research.	23-24
**FUNDING**			
Funding	27	Describe sources of funding for the systematic review and other support (e.g., supply of data); role of funders for the systematic review.	2

From: Moher D, Liberati A, Tetzlaff J, Altman DG, The PRISMA Group (2009). Preferred Reporting Items for Systematic Reviews and Meta-Analyses: The PRISMA Statement. PLoS Med 6(7): e1000097. doi:10.1371/journal.pmed1000097

Supplementary table 2. Search strategy			
	**MEDLINE**	**COCHRANE LIBRARY**	**EMBASE**
#1	"CNS"	"CNS"	cns'.exp OR 'cns'
#2	"central nervous system"	"central nervous system"	"central nervous system"
#3	neuropath*	neuropath*	neuropath*
#4	encephalopath*	encephalopath*	encephalopath*
#5	neurolog*	neurolog*	neurolog*
#6	OR/1-5	OR/1-5	OR/1-5
#7	allogeneic	allogeneic	allogeneic
#8	"HCT"	"HCT"	"HCT"
#9	"BMT"	"BMT"	"BMT"
#10	"bone marrow transplantation"	"bone marrow transplantation"	"bone marrow transplantation"

#11	"hematopoietic SCT"	"hematopoietic SCT"	"hematopoietic SCT"
#12	"stem cell transplantation"	"stem cell transplantation"	"stem cell transplantation"
#13	"hematopoietic stem cell transplantation"	"hematopoietic stem cell transplantation"	"hematopoietic stem cell transplantation"
#14	"bone marrow transplant*"	"bone marrow transplant*"	"hematopoietic stem cell transplant"
#15	"hematopoietic stem cell transplant*"	"hematopoietic stem cell transplant*"	"bone marrow transplant"
#16	"stem cell transplant*"	"stem cell transplant*"	"stem cell transplant"
#17	OR/7-16	OR/7-16	OR/7-16
#18	6 AND 17	6 AND 17	6 AND 17
#19	animals [mh]		[animals]/lim NOT [humans]/lim
#20	humans [mh]		18 NOT 19
#21	19 NOT 20		
#22	18 NOT 22		

Supplementary table 3. Case report studies' characteristics.

First author	Year	n	Type	Subtype	Onset day	Outcome	#Neuro-related deaths	#Not neuro-related deaths
Abdelkefi [1]	2004	1	Infectious	protozoan	early	resolution		
Abdullayev [2]	2015	1	Drug-related	CsA	early	resolution		
Abe [3]	2002	2	Immune-mediated	Hemophagocytic syndrome	early	death	0	2
Ackerman [4]	1986	1	Infectious	protozoan	na	resolution		
Airas[5]	2010	1	Immune-mediated	CNS IRIS	late	resolution		
Aisa [6]	2009	1	PTLD		late	death	1	
Akpek [7]	2011	1	Infectious	protozoan	late	death	1	
Al-Mujaini [8]	2009	1	Relapse	CNS	late	na		
Al-Shekhlee[9]	2001	1	Immune-mediated	PNS-GVHD	late	resolution		
Albera [10]	1997	1	Drug-related	CsA	na	resolution		
Alimam [11]	2014	2	Immune-mediated	Acute transverse myelitis	late	resolution		
Allen [12]	2009	3	Immune-mediated	Dermatomyositis	late	resolution		
Ambrosioni [13]	2010	1	Infectious	fungal	early	resolution		
Ando [14]	2010	1	Infectious	viral	late	death	1	
Andriuta [15]	2015	1	Infectious	viral	late	na		
Apsner[16]	1997	1	Drug-related	CsA	early	resolution		
Apuri[17]	2014	2	Drug-related	tacrolimus	early	resolution		
Ardissino[18]	2018	1	Relapse	CNS	late	resolution		
Arslan[19]	2010	1	Infectious	viral	early	death	1	
Aruch[20]	2015	1	Drug-related	tacrolimus	na	death	0	1
Atassi [21]	2008	1	Immune-mediated	MG	late	resolution		
Au [22]	2002	1	Immune-mediated	ADEM	late	resolution		
Au [23]	1999	2	Relapse	CNS	late	resolution(1), death(1)	1	
Avivi[24]	2014	2	Infectious	viral	late	resolution(1), death(1)	1	
Awosika [25]	2013	1	Infectious	viral	late	death	1	
Brown[26]	2014	1	Drug-related	CsA	na	resolution		
Bowen[27]	2017	1	Immune-mediated	CNS-GVHD	late	death	1	

Bolaños-Meade[28]	2005	1	Drug-related	Hydroxychloroquine	na	resolution		
Baddley [29]	2000	1	Infectious	fungal	late	death	0	1
Baek [30]	2005	1	Metabolic	Wernicke's encephalopathy	early	resolution		
Baghban[31]	2018	1	Infectious	viral	late	resolution		
Baker [32]	2011	1	Other	TTP	early	na		
Balcom[33]	2018	1	Other	T-cell leukoencephalitis	late	death		1
Baldini [34]	2010	1	Immune-mediated	Guillain-Barré	early	resolution		
Balduzzi [35]	2010	1	Infectious	viral	late	resolution		
Baron[36]	1998	1	Immune-mediated	MG	late	resolution		
Barosso[37]	2017	1	Infectious	viral	early	death		1
Baslar [38]	1998	1	Infectious	fungal	early	resolution		
Batra [39]	2015	1	Other	intracranial hypotension	early	resolution		
Batsis [40]	2015	1	Infectious	fungal	late	resolution		
Baumer [41]	2015	1	Immune-mediated	Neuromyelitis optica	early	resolution		
Beata [42]	2017	1	Drug-related	voriconazole	na	resolution		
Behr [43]	2006	1	Infectious	viral	late	resolution		
Berger [44]	2014	1	Infectious	viral	late	not resolved		
Bergin [45]	1987	1	Infectious	protozoan	early	death	1	
Bertz [46]	1998	3	Cerebrovascular	Sinus venous thrombosis	late	resolution		
Bethge[47]	1999	2	Infectious	viral	early	resolution		
Bhagat [48]	2013	1	Infectious	viral	early	death	1	
Bhunia[49]	2019	1	TA-TMA	PRES	early	resolution		
Bitan[50]	2004	1	Immune-mediated	Guillain-Barré	early	death	1	
Blecha[51]	2015	1	Immune-mediated	CNS-GVHD	late	resolution		
Bloor [52]	2006	1	Infectious	bacterial	early	death	0	1
Bocian [53]	2014	1	PTLD		late	resolution		
Bogdanov [54]	2017	1	Infectious	viral	na	death	1	
Boqué [55]	2003	1	Drug-related	CsA	early	resolution		
Borys[56]	2018	1	Immune-mediated	PNS-GVHD	late	resolution		
Borys2[57]	2018	1	Infectious	protozoan	late	resolution		
Bova[58]	2000	1	Drug-related	tacrolimus	late	resolution		
Boyer [59]	2017	1	Relapse	CNS	na	resolution		
Brenner [60]	2005	1	Infectious	viral	late	death	0	1

Brueckner [61]	2016	1	Infectious	protozoan	late	resolution		
Buckanovich[62]	2002	1	Infectious	viral	early	resolution		
Bujor [63]	2010	1	Relapse	CNS	late	resolution		
Bunyan [64]	2002	1	Immune-mediated	MG	late	resolution		
Campos [65]	2000	1	Infectious	bacterial	late	resolution		
Camus [66]	2015	1	Infectious	viral	early	resolution		
Candoni [67]	2011	2	Infectious	viral	late	not resolved (1), death (1)	1	
Cavattoni[68]	2010	1	Infectious	protozoan	early	resolution		
Capoferri [69]	2015	1	Infectious	viral	late	death	0	1
Carter [70]	2014	1	Infectious	fungal	na	death	1	
Castellano-Sanchez[71]	2003	1	Infectious	protozoan	late	death	1	
Cavrini [72]	2010	1	Infectious	fungal	late	death	1	
Ceccanti[73]	2017	1	Infectious	viral	late	death		1
Chapuis [74]	2016	1	Infectious	viral and protozoan	early	death	0	1
Chan [75]	2018	1	Infectious	protozoan	late	death		1
Chittick [76]	2013	1	Infectious	viral	early	not resolved		
Couriel[77]	2002	2	Immune-mediated	PNS-GVHD	late	resolution		
Cho [78]	2013	1	Relapse	Isolated CNS relapse	late	resolution		
Chordia [79]	2014	1	Infectious	viral	na	resolution		
Chowdhary [80]	2008	1	Infectious	viral	late	not resolved		
Claveau [81]	2017	1	Infectious	viral	late	death	1	
Choi[82]	2010	1	Metabolic	WE	early	resolution		
Carobolante[83]	2014	1	Drug-related	CsA	early	resolution		
Carrion [84]	2006	1	PTLD		early	death	0	1
Carroll[85]	2005	1	Immune-mediated	PNS-GVHD	late	not resolved		
Cocito[86]	2015	1	Immune-mediated	PNS-GVHD	late	resolution		
Collins[87]	2000	1	Immune-mediated	PNS-GVHD	late	resolution		
Colombo[88]	2012	1	Infectious	viral	late	death	1	
Cordonnier [89]	1983	1	Infectious	viral	late	resolution		
Curran [90]	2013	1	Relapse	CNS	late	resolution		
Curran [91]	2012	1	Immune-mediated	transverse myelitis	na	death	0	1
Davis[92]	1988	1	Infectious	viral	late	death	1	
Davoudi [93]	2014	1	Infectious	fungal	na	resolution		

Delaloye[94]	2011	1	Infectious	bacterial	late	resolution		
deBeukelaar[95]	2005	1	PTLD, infectious	viral	early	death	1	
de Souza Franceschi[96]	2014	1	Infectious	viral	early	resolution		
Debaere [97]	1999	1	Drug-related	CsA	na	resolution		
Dehghani [98]	2009	1	Infectious	bacterial	na	death	0	1
Devinsky[99]	1987	1	Drug-related	total-body irradiation and amphotericin B chemoprophylaxis	na	death	0	1
Diamanti[100]	2009	1	Infectious	fungal	late	not resolved		
Diamanti[101]	2015	1	Immune-mediated	AQP-4-IgG ON	late	not resolved		
Doi [102]	2012	1	Immune-mediated	PNS-GVHD	late	resolution		
Donnini [103]	2016	1	TA-TMA		early	resolution		
Dowell [104]	1999	1	Immune-mediated	MG	late	resolution		
Edwards [105]	1996	2	Drug-related	CsA	early	resolution		
El-Cheikh[106]	2012	1	Infectious	viral	early	death	1	
El-Jawahri[107]	2018	1	Infectious	viral	late	death	1	
Enriquez-Marulanda[108]	2017	1	Immune-mediated	CNS-GVHD	late	resolution		
Escuissato[109]	2004	1	Infectious	protozoan	early	death	1	
Feurer [110]	2015	1	Immune-mediated	CNS-GVHD	late	resolution		
Farid [111]	2017	1	Infectious	fungal	late	resolution		
Fonseca[112]	2017	1	Immune-mediated	CNS-GVHD	late	resolution		
Foolad[113]	2017	1	Drug-related	posaconazole	late	resolution		
Forest [114]	2011	1	Infectious	viral	early	death	1	
Fotheringham [115]	2007	3	Infectious	viral	early	death	3	
Fuchs [116]	2012	1	Relapse	CNS	late	resolution		
Fitzgerald [117]	2009	1	Infectious	viral	early	death	0	1
Freitas [118]	2014	1	Drug-related	tacrolimus	early	resolution		
Fujii [119]	2006	1	Drug-related	calcineurin inhibitor	early	death	0	1
Fujisaki[120]	2006	1	Immune-mediated	Guillain-Barré	early	resolution		
Fujiwara [121]	2014	1	Infectious	viral	late	resolution		
Fukatsu[122]	2017	1	Immune-mediated	MG	late	resolution		
Fukuno [123]	2006	1	Infectious	viral	late	resolution		
Golla [124]	2005	1	Drug-related	CsA	early	resolution		
Gabriel[125]	1999	1	Immune-mediated	PNS-GVHD	late	resolution		

Ganapule [126]	2016	1	Other	secondary malignancy	late	death	1	
Gatti-Mays[127]	2016	1	Infectious	protozoan	late	resolution		
Gauthier[128]	2017	1	Immune-mediated	CNS-GVHD	late	resolution		
Ghabrial[129]	2017	1	Infectious	fungal	early	resolution		
Gocmen [130]	2016	1	Drug-related	CsA	early	resolution		
Gong [131]	2011	1	Relapse	CNS	late	resolution		
Gonzalez [132]	2000	1	Infectious	protozoan	early	death	1	
Gonzalez[133]	2019	1	Drug-related	ruxolitinib	early	resolution		
Gopal [134]	1999	1	Drug-related	CsA	early	death	1	
Gorniak [135]	2006	4	Infectious	viral	early	not resolved (2), resolved (1) and death (1)	0	1
Giovannelli[136]	2016	1	Metabolic	WE	early	not resolved		
Graetz[137]	2017	1	Metabolic	hyperammonemia	early	resolution		
Gruhn [138]	1999	1	Infectious	viral	late	resolution		
Greenspan[139]	1990	1	Immune-mediated	PNS-GVHD	late	resolution		
Gupta [140]	2004	1	Infectious	fungal	early	death	1	
Haase [141]	2005	1	Infectious	bacterial	early	resolution		
Hackanson [142]	2005	2	Infectious	viral	early (1), late(1)	death	2	
Hamadani [143]	2007	1	PTLD		late	death	1	
Hamprecht [144]	2003	1	Infectious	viral	late	death	1	
Hanel [145]	2001	1	PTLD		late	resolution		
Häntschel [146]	2013	1	Other	Hippocampal sclerosis	early	death	1	
Haruki[147]	2012	1	Immune-mediated	PNS-GVHD	late	resolution		
Harvey[148]	2000	2	Cerebrovascular		early	resolution		
Harvey[149]	2014	1	Immune-mediated	CNS-GVHD	early	resolution		
Hatsuta [150]	2013	1	Immune-mediated		late	death	1	
Hattori [151]	2016	1	Infectious	viral	early	death	1	
Heidarzadeh [152]	2014	1	Immune-mediated	MG	late	resolution		
Heim [153]	2016	1	Infectious	viral	early	death	1	
Helton [154]	2016	1	Infectious	protozoan	late	death	0	1
Hernández-Boluda[155]	2005	1	Immune-mediated	Guillain-Barré	early	death	1	
Herraez [156]	2015	1	TA-TMA		early	resolution		
Heurkens[157]	1989	1	Infectious	protozoan	early	death	1	

Hiatt [158]	2003	1	Infectious	viral	late	death	1	
Hill[159]	2017	1	Immune-mediated	MG	late	resolution		
Hino [160]	2016	1	Infectious	viral	early	not resolved		
Hino [161]	2016	1	Infectious	bacterial	late	resolution		
Hirst [162]	1983	1	Infectious	viral	early	na		
Hoefnagels [163]	1988	1	Drug-related	CsA	early	resolution		
Hon [164]	2005	1	Infectious	viral	late	resolution		
Hong [165]	2003	2	Infectious	viral	early (1), late(1)	death	2	
Hossain [166]	2015	1	Drug-related	tacrolimus	early	resolution		
Hsiao [167]	2014	1	Cerebrovascular		early	resolution		
Hubele [168]	2012	1	Infectious	viral	early	resolution		
Ikegawa[169]	2018	1	Infectious	viral	early	death		1
Ikegame [170]	2013	1	Infectious	viral	early	resolution		
Ibrahim [171]	2016	1	Infectious	bacterial	late	resolution		
Im [172]	2012	1	Infectious	fungal	early	resolution		
Imataki [173]	2015	1	Infectious	viral	early	death	1	
Imataki [174]	2014	1	Drug-related	tacrolimus	early	resolution		
Ionita [175]	2004	1	Infectious	protozoan	late	death	1	
Jehn [176]	1984	1	Infectious	protozoan	early	death	1	
Jennane [177]	2013	1	Drug-related	CsA	early	resolution		
Johnson [178]	2011	1	Infectious	bacterial	late	na		
Kamble[179]	2007	2	Immune-mediated	CNS-GVHD	late	resolution(1), death(1)	1	
Kamble[180]	2014	2	Immune-mediated	PNS-GVHD	early	resolution(1), death(1)	0	1
Kanamori[181]	2001	1	Infectious	viral	early	resolution		
Kanter[182]	2011	1	Immune-mediated	PNS-GVHD	late	not resolved		
Kapp [183]	2011	1	Infectious	fungal	na	death	1	
Kaufman[184]	2014	1	Infectious	viral	late	death	1	
Kaushik[185]	2002	1	Immune-mediated	PNS-GVHD	late	resolution		
Kittan [186]	2011	1	PTLD		late	death	1	
Kawano [187]	2000	1	Infectious	viral	early	death	1	
Kawahara[188]	2018	1	Infectious	fungal	late	death	1	
Kawaguchi [189]	2013	1	Infectious	viral	early	resolution		

Kawamata[190]	2014	1	Immune-mediated	HTLV-I associated myelopathy	late	resolution		
Kearney[191]	2010	1	Immune-mediated	CNS-GVHD	late	not resolved		
Kelly[192]	1996	1	Immune-mediated		late	resolution		
Kew[193]	2007	1	Immune-mediated	CNS-GVHD	late	resolution		
Khalaf [194]	2017	1	Infectious	protozoan	late	resolution		
Khan[195]	2016	1	Immune-mediated	PNS-GVHD	late	resolution		
Kharfan-Dabaja[196]	2007	2	Infectious	viral	late	death	2	
Khoury [197]	1999	1	Infectious	protozoan	late	resolution		
Kida [198]	2004	2	Drug-related	calcineurin inhibitor	early	resolution(1), death(1)	0	1
Kim[199]	2002	2	Infectious	viral	early	resolution		
Kleiter[200]	2014	1	Immune-mediated	PNS-GVHD	late	death	0	1
Kishida [201]	2010	1	Infectious	viral	late	resolution		
Koide[202]	2000	1	Drug-related	CsA	early	death	1	
Kondo [203]	2014	1	Relapse	CNS	late	resolution		
Kremer[204]	2010	1	Infectious	viral	early	not resolved		
Kuroshima[205]	2017	4	Infectious	viral	early	na		
Kural[206]	2018	1	Infectious	fungal	early	death		1
Lacerda [207]	2005	1	Infectious	fungal	late	death	1	
Lawson[208]	2018	1	Drug-related	tacrolimus	late	resolution		
LeCalloch [209]	2014	3	Drug-related	CsA	early (2), late (1)	death(2),resolution(1)	1	1
Lieberman [210]	2012	1	PTLD		late	resolution		
Liguori[211]	2000	1	Immune-mediated	PNS-GVHD	early	resolution		
Liu[212]	2007	1	Immune-mediated	PNS-GVHD	late	resolution		
Lee [213]	2005	1	Infectious	protozoan	early	death	1	
Liapis [214]	2009	1	Infectious	fungal	late	death	0	1
Lim[215]	2008	1	Other	central pontine myelinolysis	early	death	1	
Lind [216]	1989	1	Drug-related	CsA	early	death	1	
Liu [217]	2015	1	TA-TMA		early	death	1	
Long[218]	1993	3	Infectious	bacterial	late	resolution		
Lopes da Silva [219]	2011	1	Infectious, TMA	viral	late	death	1	
Lee [220]	2014	2	Infectious	viral	late	death	2	

Lee [221]	2008	2	Drug-related	calcineurin inhibitor	early	resolution		
Lee [222]	2013	1	Infectious	viral	early	death	0	1
Lee [223]	2010	1	Infectious	viral	late	death	1	
Lee [224]	2017	1	Relapse	CNS	late	resolution		
Lee[225]	2018	1	Relapse	CNS	late	resolution		
Lopez-Jimenez[226]	1997	1	Drug-related	CsA	late	resolution		
Leano[227]	2000	1	Immune-mediated	PNS-GVHD	late	death	1	
Loseto [228]	2012	1	Drug-related	CsA	late	resolution		
Lotze [229]	2005	1	Relapse	CNS	early	resolution		
Lowenberg [230]	1983	1	Infectious	protozoan	early	death	1	
Lorenzoni[231]	2007	1	Immune-mediated	PNS-GVHD	late	resolution		
Lux [232]	2007	1	Drug-related	tacrolimus	late	resolution		
Maximova[233]	2012	1	Infectious	fungal	early	death	1	
Madan [234]	1997	1	Drug-related	CsA	early	resolution		
Mackey [235]	1997	1	Immune-mediated	MG	late	not resolved		
Mahajan [236]	2016	1	Relapse	CNS	na	not resolved		
Miklavcic[237]	2017	2	Drug-related	CsA	early	not resolved(1),death(1)	0	1
Miyagi [238]	2015	2	Infectious	protozoan	early (1), late(1)	resolution		
Mohty [239]	2010	1	Infectious	fungal	late	death	1	
Mori [240]	2000	1	Drug-related	CsA	early	death	1	
Machetti [241]	2000	1	Infectious	fungal	early	resolution		
Majeed [242]	2017	1	Infectious	bacterial	late	resolution		
Malkan[243]	2018	1	Drug-related	tacrolimus	early	resolution		
Manabe [244]	2010	1	Drug-related	CsA	early	resolution		
Marosi[245]	1990	1	Immune-mediated	CNS-GVHD	late	death	1	
Martino [246]	2012	1	Infectious	viral	late	death	1	
Matsukawa [247]	2012	1	Infectious	viral	late	death	1	
Matsuo[248]	2009	1	Immune-mediated	CNS-GVHD	na	resolution		
Maurice[249]	2018	1	Immune-mediated	CNS-GVHD	late	resolution		
Meignin [250]	1998	2	Other	secondary benign tumors	late	resolution		
Meng [251]	2012	1	Immune-mediated	PNS-GVHD	late	na		
Meng[252]	2017	2	Immune-mediated	PNS-GVHD	late	resolution		

Messina [253]	2007	2	Metabolic	WE	early	resolution			
Meyer [254]	2002	1	Drug-related	CsA	late	na			
Michelis[255]	2015	1	Immune-mediated	PNS-GVHD	late	resolution			
Mielke [256]	2007	1	Drug-related	CSA, fludarabine or a combination	early	death	1		
Miura[257]	2006	1	Drug-related	fludarabine	early	not resolved			
Moesen[258]	2014	1	Immune-mediated	CNS-GVHD	late	not resolved			
Miller[259]	2006	1	Infectious	viral	late	death	1		
Monteiro De Almeida [260]	2003	1	Infectious	bacterial	early	death	1		
Mori [261]	2007	2	Infectious	viral	early	resolution(1), death(1)	1		
Moskowitz [262]	2007	1	Drug-related	sirolimus	early	resolution			
Motohashi[263]	2010	1	Immune-mediated	CNS-GVHD	late	resolution			
Mueller-Mang[264]	2006	2	Infectious	protozoan	late	death	2		
Muftuoglu[265]	2018	1	Infectious	viral	late	resolution			
Maillard-Lefebvre[266]	2010	1	Immune-mediated	PNS-GVHD	late	resolution			
Nagafuji [267]	1998	1	PTLD		late	death	0	1	
Najera [268]	2013	1	Drug-related	tacrolimus	early	resolution			
Nanno[269]	2016	1	Infectious	fungal	early	death	1		
Nenoff [270]	1998	1	Infectious	fungal	late	death	1		
Noda [271]	2008	1	Drug-related	calcineurin inhibitor	early	resolution			
Nozzoli [272]	2006	1	PTLD		early	resolution			
Neumann [273]	2011	3	Relapse	CNS	late	not resolved (2), resolution (1)			
Nagashima[274]	2002	1	Immune-mediated	PNS-GVHD	late	not resolved			
Neppalli[275]	2016	1	Immune-mediated	CNS-GVHD	late	resolution			
Nakazato[276]	2003	1	Drug-related	tacrolimus	early	resolution			
Ng[277]	2018	1	Immune-mediated	ADEM	early	resolution			
Nakayama[278]	2019	1	Immune-mediated	CNS-GVHD	late	resolution			
O'Shaughnessy [279]	1994	1	Infectious	viral	late	death	1		
Ostronoff [280]	2008	2	Relapse	CNS	late	resolution(1), death(1)	1		
Ostronoff[281]	2007	1	Immune-mediated	Guillain–Barré	early	not resolved			
O'Toole [282]	2015	1	Infectious	viral	late	death	1		
Ozcelik [283]	2013	1	Immune-mediated	Guillain–Barré	late	death	0	1	
Onose[284]	2002	1	Drug-related	Haloperidol and tacrolimus	early	resolution			

Ohashi [285]	2005	2	Relapse	CNS	late	death	0	2
Pace [286]	1995	2	Drug-related	CsA	early	resolution		
Parameswaran[287]	2017	1	Infectious	bacterial	late	death	1	
Peman [288]	2008	1	Infectious	protozoan	early	death	1	
Pelosini[289]	2008	1	Infectious	viral	late	not resolved		
Picardi[290]	1998	1	Infectious	protozoan	late	not resolved		
Philippen[291]	2017	1	Immune-mediated	CNS-GVHD	late	resolution		
Pilo [292]	2009	1	Immune-mediated	GVHD	late	resolution		
Pirotte[293]	2018	1	Immune-mediated	CNS-GVHD	late	resolution		
Pavlović [294]	2011	1	Metabolic		early	resolution		
Petrova-Drus[295]	2015	2	Infectious	protozoan	na	death	2	
Polchlopek Blasiak[296]	2017	1	Immune-mediated	CNS-GVHD	early	resolution		
Powell[297]	2018	1	PTLD		late	resolution		
Qu [298]	2013	1	Drug-related	CsA and voriconazole	early	resolution		
Rabinstein[299]	2003	1	Immune-mediated	Acute neuropathies	early	resolution		
Rodriguez[300]	2002	3	Immune-mediated	Guillain–Barré	early	death	0	3
Raheja[301]	2017	1	Other	Epstein-Barr virus-associated smooth muscle tumors	late	resolution		
Rasool[302]	2018	3	Drug-related	tacrolimus	late	resolution		
Raza [303]	2007	1	Drug-related	CsA	early	resolution		
Reddy[304]	2010	2	Infectious	viral	late	death	2	
Ricci [305]	2009	1	Immune-mediated		late	resolution		
Richard[306]	2000	1	Immune-mediated		late	not resolved		
Resnick [307]	2004	1	Drug-related	steroids with iatrogenic Cushing's syndrome	late	death	0	1
Rieux [308]	1998	1	Infectious	viral	late	resolution		
Roy [309]	2000	1	Infectious	fungal	early	resolution		
Reddy [310]	2004	1	Infectious	viral	early	death	1	
Robuccio[311]	2015	1	Immune-mediated	CNS-GVHD	early	not resolved		
Ramlal[312]	2016	1	Other	Multifocal Necrotizing Leukoencephalopathy	early	death	1	
Renner [313]	2004	1	Cerebrovascular		early	death	1	
Reynolds[314]	2017	1	Infectious	viral	na	not resolved		
Rizvi[315]	2017	1	Drug-related	tacrolimus	late	resolution		

Saad[316]	2009	1	Immune-mediated	CNS-GVHD	late	death	0	1
Sakamoto[317]	2012	1	Immune-mediated	PNS-GVHD	late	resolution		
Sakai[318]	2006	1	Immune-mediated	immune-mediated myelopathy	late	resolution		
Sarva [319]	2012	1	Infectious	viral	late	death	1	
Sawant[320]	2005	1	Other	TTP	early	death	0	1
Sekiguchi[321]	2013	1	Immune-mediated	PNS-GVHD	late	death	0	1
Seok [322]	2011	1	Infectious	viral	early	death	0	1
Shargian-Alon[323]	2019	1	Immune-mediated	CNS-GVHD	late	death	1	
Shbarou [324]	2000	1	Drug-related	CsA	early	not resolved		
Sheikh [325]	2009	1	Infectious	viral	late	death	0	1
Shimizu [326]	2011	1	PTLD		late	death	0	1
Shigemura [327]	2016	1	Infectious	fungal	late	death	1	
Short[328]	2019	1	PTLD		early	death	1	
Shortt[329]	2006	1	Immune-mediated	CNS-GVHD	late	resolution		
Solh [330]	2014	1	Immune-mediated	Guillain-Barré	late	resolution		
Solmaz[331]	2016	1	Metabolic	WE	early	resolution		
Sostak[332]	2010	4	Immune-mediated	CNS-GVHD	late	resolution (2), death(2)	0	2
Steg[333]	1999	1	Drug-related	tacrolimus	early	resolution		
Shintaku[334]	2010	1	Infectious	viral	early	death	0	1
Shimizu[335]	2017	1	Infectious	viral	late	resolution		
Schenk[336]	2011	1	Infectious	viral	early	death	1	
Solaro[337]	2001	1	Immune-mediated	GVHD	early	not resolved		
Stojanoski [338]	2011	2	Other	PRES	early	resolution		
Steurer[339]	2003	1	Infectious	viral	late	death	0	1
Sassi[340]	2014	1	Drug-related	CsA	early	resolution		
Sinaei[341]	2018	2	Immune-mediated	CIDP	early (1), late (1)	resolution		
Seong[342]	1993	1	Infectious	protozoan	early	death	1	
Schrader[343]	2011	1	Immune-mediated	CNS-GVHD	late	resolution		
Steinberg[344]	2015	1	Metabolic	WE	na	resolution		
Sweany[345]	2007	1	Other	PRES	early	death	1	
Saikawa[346]	2008	1	Immune-mediated		late	resolution		
Sanchez-Quintana[347]	2013	1	Infectious	viral	early	resolution		

Suzuki[348]	2007	1	Immune-mediated	PNS-GVHD	late	death	0	1
Sevindik [349]	2015	1	TA-TMA		early	resolution		
Sakai [350]	2005	1	Relapse	CNS	late	resolution		
Schmidt-Hieber[351]	2005	1	Immune-mediated	PNS-GVHD	late	death	0	1
Suzuki [352]	2012	1	Infectious	viral	late	not resolved		
Targhetta[353]	2013	2	Immune-mediated	Miller-fisher	late	resolution(1), death(1)	1	
Tamaki[354]	2004	1	Drug-related	tacrolimus	early	resolution		
Tang[355]	2018	1	Other	TTP-like syndrome with arcanobacterium pyogenes endocarditis	late	death	1	
Takeda[356]	1998	1	Immune-mediated	CNS-GVHD	early	resolution		
Tauro[357]	2000	1	Infectious	viral	late	resolution		
Takahashi [358]	2009	1	Infectious	fungal	na	death	1	
Tanaka[359]	2005	2	Infectious	viral	early	death (2)	2	
Takahashi [360]	2013	1	Infectious	viral	early	death	0	1
Tănase [361]	2011	1	Infectious	fungal	early	resolution		
Tomaszewska[362]	2010	1	Infectious, Immune-mediated	viral	late	resolution		
Thien [363]	2016	1	Infectious	fungal	late	not resolved		
Terada[364]	2017	1	Immune-mediated	CNS-GVHD	late	resolution		
Tseng[365]	2015	1	Infectious	viral	late	death	0	1
Teksam[366]	2001	1	Drug-related	CsA	early	death	0	1
Thomas [367]	2010	1	Relapse	CNS	late	resolution		
Terrettaz[368]	2008	1	Immune-mediated	CNS-GVHD	late	resolution		
Takahashi[369]	2000	1	Immune-mediated	PNS-GVHD	late	resolution		
Takahata [370]	2001	1	Drug-related	CsA	early	resolution		
Takatsuka[371]	2000	1	Immune-mediated	CNS vasculitis	early	resolution		
Tamaki [372]	2016	1	Infectious	fungal	late	death	1	
Tateno[373]	2017	1	Infectious	protozoan	early	death	1	
Terasawa [374]	2002	1	PTLD		late	death	1	
Thomas [375]	1999	1	Infectious	fungal	late	death	1	
Thone[376]	2010	1	Immune-mediated	GVHD	late	resolution		
To [377]	1983	1	Relapse	CNS	late	death	0	1
Tomonari[378]	2003	1	Immune-mediated	ADEM	late	resolution		

Tomoki Iemura[379]	2019	1	Drug-related	metronidazole	late	death		1
Torelli[380]	2011	1	Drug-related	CsA	early	resolution		
Toumeh [381]	2012	1	Relapse	CNS	late	resolution		
Tummala[382]	2018	1	Infectious	viral	late	resolution		
Urbano-Marquez[383]	1986	1	Immune-mediated	PNS-GVHD	late	resolution		
Uoshima[384]	2000	1	Drug-related	CsA	early	resolution		
Vande Broek [385]	2009	1	Infectious	fungal	late	death	1	
Vasko [386]	2011	1	TA-TMA		na	resolution		
Vesole[387]	2018	1	Drug-related	CsA	late	resolution		
Vilter[388]	1986	1	Drug-related	Vidarabine	late	resolution		
Visser [389]	2006	1	Infectious	viral	early	not resolved		
Vogl[390]	2011	1	Immune-mediated	CIDP	late	resolution		
Voss[391]	2010	2	Other	acute myelitis	early (1), late(1)	resolution		
Warwick [392]	1991	1	Infectious	fungal	na	death	1	
Wei[393]	2018	1	Drug-related	tacrolimus	early	resolution		
Wylie [394]	2016	1	Infectious	bacterial	late	resolution		
Wadhwani[395]	2015	1	Immune-mediated	CNS-GVHD	early	death	1	
Ward [396]	2002	1	Infectious	viral	late	not resolved		
Wroblewska[397]	2015	1	PTLD		early	resolution		
Walter[398]	2000	1	Drug-related	CsA	late	resolution		
Yacoub [399]	2014	1	Infectious	viral	late	resolution		
Yadav[400]	2018	1	Infectious	viral	late	resolution		
Yoshida[401]	2014	1	Infectious	viral	na	resolution		
Yu[402]	2016	1	Immune-mediated	CNS-GVHD	late	resolution		
Yoo [403]	2012	1	Immune-mediated	CNS-GVHD	na	death	1	
Yamashita [404]	2016	1	Infectious	viral	late	death	1	
Yoshihara[405]	2004	1	Infectious	viral	early	resolution		
Young [406]	2007	1	Infectious	fungal	late	death	1	
Yafour[407]	2016	1	Drug-related	CsA	early	resolution		
Yamamoto[408]	2009	1	Immune-mediated	CNS-GVHD	early	death	1	
Yeral [409]	2014	1	Immune-mediated	Guillain-Barré	late	resolution		
Yoo [410]	2013	1	Infectious	protozoan	late	death	1	

Yoon[411]	2012	1	Immune-mediated	CNS-GVHD	late	resolution		
Yamashita[412]	2018	1	Infectious	viral	late	death	1	
Yildirim[413]	2017	2	Infectious	fungal	early (1), late (1)	death	2	
Yoshida [414]	2002	1	Infectious	viral	early	resolution		
Yuan[415]	2018	1	Infectious	viral	late	death	1	
Yoshida[416]	2016	1	Immune-mediated	Guillain-Barré	late	resolution		
Zaucha-Prazmo[417]	2017	1	Infectious	protozoan	late	resolution		
Zhao[418]	2018	2	Infectious	viral	early	resolution		
Zhu[419]	2014	1	Relapse	peripheral	late	not resolved		
Zheng [420]	2012	1	Infectious	viral	late	resolution		
Zucchetti [421]	2013	1	PTLD		late	death	1	
Zeiser[422]	2004	1	Infectious	viral	late	death	1	
Zangrandi[423]	2018	1	Higher Cortical Functions		late	not resolved		
Zaja [424]	1997	1	Immune-mediated	MG	late	resolution		
Zaja [425]	2000	1	Immune-mediated	MG	late	resolution		

N= number of patients ≥ 15 years old who underwent allogeneic hematopoietic cell transplant for hematologic diseases, ADEM: Acute disseminated encephalomyelitis, AQP-4-IgG ON: anti-aquaporin-4 Abs positive optic neuritis, CsA: Cyclosporine A, CIDP: chronic inflammatory demyelinating polyneuropathy, HTLV-I: human T-cell leukemia virus type I, PTLD: Post-Transplantation Lymphoproliferative Disorder, PRES: posterior reversible encephalopathy, GVHD: graft versus host disease, CNS: Central Nervous System, na: not applicable, TA-TMA: Transplant-associated thrombotic microangiopathy, PNS: peripheral nervous system, TTP: Thrombotic thrombocytopenic purpura, MG; myasthenia gravis, IRIS: immune reconstitution inflammatory syndrome, WE: Wernicke's encephalopathy , #: number

1. Abdelkefi A, Ben Othman T, Torjman L, et al. Plasmodium falciparum causing hemophagocytic syndrome after allogeneic blood stem cell transplantation. *The hematology journal : the official journal of the European Haematology Association.* 2004; 5(5):449-450.
2. Abdullayev ES, B.; Guvenc, B. Case report: Posterior reversible encephalopathy syndrome in a patient after allogeneic hematopoietic stem cell transplantation. *Leukemia research.* 2015:S33-S34.
3. Abe Y, Choi I, Hara K, et al. Hemophagocytic syndrome: a rare complication of allogeneic nonmyeloablative hematopoietic stem cell transplantation. *Bone marrow transplantation.* 2002; 29(9):799-801.
4. Ackerman Z, Or R, Maayan S. Cerebral toxoplasmosis complicating bone marrow transplantation. *Israel journal of medical sciences.* 1986; 22(7-8):582-586.
5. Airas L, Paivarinta M, Roytta M, et al. Central nervous system immune reconstitution inflammatory syndrome (IRIS) after hematopoietic SCT. *Bone marrow transplantation.* 2010; 45(3):593-596.
6. Aisa Y, Mori T, Nakazato T, et al. Primary central nervous system post-transplant lymphoproliferative disorder presenting as cerebral hemorrhage after unrelated bone marrow transplantation. *Transplant infectious disease : an official journal of the Transplantation Society.* 2009; 11(5):438-441.
7. Akpek G, Uslu A, Huebner T, et al. Granulomatous amebic encephalitis: an under-recognized cause of infectious mortality after hematopoietic stem cell transplantation. *Transplant infectious disease : an official journal of the Transplantation Society.* 2011; 13(4):366-373.
8. Al-Mujaini AS, Al-Dhuhli HH, Dennison DJ. Acute unilateral third nerve palsy as an early manifestation of central nervous system relapse in a patient with acute myeloid leukemia. *Saudi medical journal.* 2009; 30(7):961-963.
9. Al-Shekhlee A, Katirji B. Sensory Mononeuropathy Multiplex in Chronic Graft versus Host Disease. *Journal of clinical neuromuscular disease.* 2001; 2(4):184-186.
10. Albera R, Luda E, Canale G, et al. Cyclosporine a as a possible cause of upbeating nystagmus. *Neuro-Ophthalmology.* 1997; 17(3):163-168.

11. Alimam SK, S.; Tholouli, E.; McKee, D.; Gibbs, S.; Haigh, D.; Rys-Halska, S.; Dignan, F. Successful treatment of acute transverse myelitis occurring post haematopoietic stem cell transplantation. *Bone marrow transplantation.* 2014; 49:S477.
12. Allen JA, Greenberg SA, Amato AA. Dermatomyositis-like muscle pathology in patients with chronic graft-versus-host disease. *Muscle & nerve.* 2009; 40(4):643-647.
13. Ambrosioni J, van Delden C, Krause KH, et al. Invasive microsporidiosis in allogeneic haematopoietic SCT recipients. *Bone marrow transplantation.* 2010; 45(7):1249-1251.
14. Ando T, Mitani N, Yamashita K, et al. Cytomegalovirus ventriculoencephalitis in a reduced- intensity conditioning cord blood transplant recipient. *Transplant infectious disease : an official journal of the Transplantation Society.* 2010; 12(5):441-445.
15. Andriuta D, Tir M, Perin B, et al. Teaching NeuroImages: epilepsia partialis continua revealing PML after allogenic stem cell transplantation. *Neurology.* 2015; 85(5):e53-54.
16. Apsner R, Schulenburg A, Steinhoff N, et al. Cyclosporin A-induced ocular flutter after marrow transplantation. *Bone marrow transplantation.* 1997; 20(3):255-256.
17. Apuri S, Carlin K, Bass E, Nguyen PT, Greene JN. Tacrolimus associated posterior reversible encephalopathy syndrome - a case series and review. *Mediterranean journal of hematology and infectious diseases.* 2014; 6(1):e2014014.
18. Ardissino G, Cresseri D, Giglio F, et al. Haploidentical Hematopoietic Stem Cell Transplant Complicated by Atypical Hemolytic Uremic Syndrome and Kidney Transplant from the Same Donor with No Immunosuppression but C5 Inhibition. *Transplantation.* 2019; 103(2):E48-E51.
19. Arslan F, Tabak F, Avsar E, et al. Ganciclovir-resistant cytomegalovirus encephalitis in a hematopoietic stem cell transplant recipient. *Journal of neurovirology.* 2010; 16(2):174-178.
20. Aruch DB, Renteria A. Simultaneous PRES and TMA secondary to tacrolimus after allogeneic bone marrow transplant. *Blood.* 2015; 125(25):3963-3963.
21. Atassi N, Amato AA. Muscle-specific kinase (MuSK) antibody-associated myasthenia gravis after bone marrow transplantation. *Muscle & nerve.* 2008; 38(2):1074-1075.
22. Au WY, Lie AK, Cheung RT, et al. Acute disseminated encephalomyelitis after para-influenza infection post bone marrow transplantation. *Leukemia & lymphoma.* 2002; 43(2):455-457.
23. Au WY, Lie AK, Liang R, Kwong YL. Isolated extramedullary relapse of acute lymphoblastic leukaemia after allogeneic bone marrow transplantation. *Bone marrow transplantation.* 1999; 24(10):1137-1140.
24. Avivi I, Wittmann T, Henig I, et al. Development of multifocal leukoencephalopathy in patients undergoing allogeneic stem cell transplantation-can preemptive detection of John Cunningham virus be useful? *International journal of infectious diseases : IJID : official publication of the International Society for Infectious Diseases.* 2014; 26:107-109.
25. Awosika OO, Lyons JL, Ciarlini P, et al. Fatal adenovirus encephalomyeloradiculitis in an umbilical cord stem cell transplant recipient. *Neurology.* 2013; 80(18):1715-1717.
26. Brown C. Full court PRES: Altered mental status in the immunecompromised. *Journal of General Internal Medicine.* 2014.
27. Bowen T, Silver S, Sila C. Rapidly Progressive Occlusive Intracranial Vasculopathy in Graft-versus-Host-Disease (P1.299). *Neurology.* 2017; 88(16 Supplement):P1.299.
28. Bolanos-Meade J, Zhou L, Hoke A, Corse A, Vogelsang G, Wagner KR. Hydroxychloroquine causes severe vacuolar myopathy in a patient with chronic graft-versus-host disease. *American journal of hematology.* 2005; 78(4):306-309.
29. Baddley JW, Moser SA, Sutton DA, Pappas PG. Microascus cinereus (Anamorph scopulariopsis) brain abscess in a bone marrow transplant recipient. *Journal of clinical microbiology.* 2000; 38(1):395-397.
30. Baek JH, Sohn SK, Kim DH, et al. Wernicke's encephalopathy after allogeneic stem cell transplantation. *Bone marrow transplantation.* 2005; 35(8):829-830.
31. Baghban A, Malinis M. Ganciclovir and foscarnet dual-therapy for cytomegalovirus encephalitis: A

case report and review of the literature. *Journal of the Neurological Sciences.* 2018; 388:28-36.
32. Baker J. Post-transplant thrombotic thrombocytopenic purpura: A haematological emergency; a nursing challenge. *Bone marrow transplantation.* 2011; 46:S393.
33. Balcom E, Branton W, Edguer B, et al. Delayed onset of CD8+ T cell leukoencephalitis after bone marrow transplantation. *Neurology.* 2018; 90(15).
34. Baldini SN, C.; Gozzini, A.; Bartolozzi, B.; Guidi, S.; Bosi, A. Guillain-Barré sindrome within Epstein-Barr virus reactivation after allogeneic haematopoietic stem cell transplantation. *Bone marrow transplantation.* 2010:S215-S216.
35. Balduzzi A, Lucchini G, Hirsch HH, et al. Polyomavirus JC-targeted T-cell therapy for progressive multiple leukoencephalopathy in a hematopoietic cell transplantation recipient. *Bone marrow transplantation.* 2011; 46(7):987-992.
36. Baron F, Sadzot B, Wang F, Beguin Y. Myasthenia gravis without chronic GVHD after allogeneic bone marrow transplantation. *Bone marrow transplantation.* 1998; 22(2):197-200.
37. Barosso IR, Miszczuk GS, Ciriaci N, et al. Activation of insulin-like growth factor 1 receptor participates downstream of GPR30 in estradiol-17beta-D-glucuronide-induced cholestasis in rats. *Archives of toxicology.* 2018; 92(2):729-744.
38. Başlar Z, Soysal T, Hancı M, et al. Successfully treated invasive central nervous system aspergillosis in an allogeneic stem cell transplant recipient. *Bone marrow transplantation.* 1998; 22(4):404-405.
39. Batra A, Berkowitz A. Clinical Reasoning: A 50-year-old man with headache and cognitive decline. *Neurology.* 2015; 85(24):e182-186.
40. Physicians Poster SessionsPoster Session / Day 1. *Bone marrow transplantation.* 2015; 50 Suppl 1:S117-311.
41. Baumer FM, Kamihara J, Gorman MP. Neuromyelitis optica in an adolescent after bone marrow transplantation. *Pediatric neurology.* 2015; 52(1):119-124.
42. Beata S, Donata UK, Jaroslaw D, Tomasz W, Anna WH. Influence of CYP2C19*2/*17 genotype on adverse drug reactions of voriconazole in patients after allo-HSCT: a four-case report. *Journal of cancer research and clinical oncology.* 2017; 143(6):1103-1106.
43. Behr J, Schaefer M, Littmann E, Klingebiel R, Heinz A. Psychiatric symptoms and cognitive dysfunction caused by Epstein-Barr virus-induced encephalitis. *European psychiatry : the journal of the Association of European Psychiatrists.* 2006; 21(8):521-522.
44. Berger MD, Meisel A, Andres M, Schanz U, Schwarz U, Stussi G. Unusual case of progressive multifocal leukoencephalopathy after allogeneic hematopoietic stem-cell transplantation. *Journal of clinical oncology : official journal of the American Society of Clinical Oncology.* 2014; 32(9):e33-34.
45. Bergin M, Menser MA, Procopis PG, Roy LP, Shaw PJ, Stevens MM. Central nervous system toxoplasmosis and hemolytic uremic syndrome. *The New England journal of medicine.* 1987; 317(24):1540-1541.
46. Bertz H, Laubenberger J, Steinfurth G, Finke J. Sinus venous thrombosis: an unusual cause for neurologic symptoms after bone marrow transplantation under immunosuppression. *Transplantation.* 1998; 66(2):241-244.
47. Bethge W, Beck R, Jahn G, Mundinger P, Kanz L, Einsele H. Successful treatment of human herpesvirus-6 encephalitis after bone marrow transplantation. *Bone marrow transplantation.* 1999; 24(11):1245-1248.
48. Bhagat RK, Zieske AW, Kamble RT. Cerebrospinal leukemoid reaction secondary to VZV meninigoencephalitis in an AML patient post allogeneic bone marrow transplantation. *Blood.* 2013; 122(2):300-301.
49. Bhunia N, Abu-Arja R, Bajwa RPS, Auletta JJ, Rangarajan HG. Eculizumab to Treat Posterior Reversible Encephalopathy Syndrome Due to Underlying Transplant-Associated Thrombotic Microangiopathy in Patients Receiving Allogeneic Hematopoietic Cell Transplant for Sickle Cell Disease. *Biology of Blood and Marrow Transplantation.* 2019; 25(3):S140.
50. Bitan M, Or R, Shapira MY, et al. Early-onset Guillain-Barre syndrome associated with reactivation of Epstein-Barr virus infection after nonmyeloablative stem cell transplantation. *Clinical infectious diseases : an official publication of the Infectious Diseases Society of America.* 2004; 39(7):1076-1078.

51. Blecha C, Angstwurm K, Wolff D, et al. Retinal Involvement in a Patient with Cerebral Manifestation of Chronic Graft-Versus-Host-Disease. *Oncology research and treatment.* 2015; 38(10):532-534.
52. Bloor AJ, Mackinnon S. Cerebral tuberculosis post stem cell transplant. *European journal of haematology.* 2006; 77(5):456.
53. Bocian J, Januszkiewicz-Lewandowska D. Utility of quantitative EBV DNA measurements in cerebrospinal fluid for diagnosis and monitoring of treatment of central nervous system EBV-associated post-transplant lymphoproliferative disorder after allogenic hematopoietic stem cell transplantation. *Annals of transplantation.* 2014; 19:253-256.
54. Jahrestagung der Deutschen, Osterreichischen und Schweizerischen Gesellschaften fur Hamatologie und Medizinische Onkologie, Stuttgart, 29. September-3. Oktober 2017: Abstracts. *Oncology research and treatment.* 2017; 40 Suppl 3:1-308.
55. Boque C, Petit J, Aguilera C, Vicente L, Granena A. Central and extrapontine myelinolysis following allogeneic peripheral haematopoietic progenitor cell transplantation. Favourable outcome in a patient with chronic myeloid leukaemia. *Bone marrow transplantation.* 2003; 31(1):61-64.
56. Borys E, Pambuccian S. Granulomatous myositis as sole manifestation of graft versus host disease. *Journal of neuropathology and experimental neurology.* 2018; 77(6):503-504.
57. Borys E, Alexander Jones G, Pambuccian SE. Toxoplasma in crush preparation of the brain biopsy from a cord blood hematopoietic stem cell transplant recipient. *Diagnostic cytopathology.* 2018; 46(12):1073-1076.
58. Bova D, Shownkeen H, Goldberg K, Horowitz S, Azar-Kia B. Delayed transient neurologic toxicity due to tacrolimus: CT and MRI. *Neuroradiology.* 2000; 42(9):666-668.
59. Boyer E, Rogers T, Solomon A. CNS Recurrence of AML Presenting as Cerebellitis (P5.178). *Neurology.* 2017; 88(16 Supplement):P5.178.
60. Brenner W, Storch G, Buller R, Vij R, Devine S, DiPersio J. West Nile Virus encephalopathy in an allogeneic stem cell transplant recipient: use of quantitative PCR for diagnosis and assessment of viral clearance. *Bone marrow transplantation.* 2005; 36(4):369-370.
61. Brueckner FF, S.; Wittke, C.; Lakner, J.; Große-Thie, C.; Henze, L.; Junghanss, C. Cerebral toxoplasmosis and probable invasive mucormycosis in an immunocompromised patient after hematopoietic stem cell transplantation. *Oncology research and treatment.* 2016; 39:187.
62. Buckanovich RJ, Liu G, Stricker C, et al. Nonmyeloablative allogeneic stem cell transplantation for refractory Hodgkin's lymphoma complicated by interleukin-2 responsive progressive multifocal leukoencephalopathy. *Annals of hematology.* 2002; 81(7):410-413.
63. Bujor LA, M. I. Central nervous system (CNS) complications of multiple myeloma (MM): Myelomatous meningitis after allogenic stem cell transplantation (ASCT). *Neuro-oncology.* 2010.
64. Bunyan R, Gardner B, Baize T, et al. Myasthenia Gravis After Bone Marrow Transplantation for Chronic Myelocytic Leukemia: Relationship to Chronic Graft versus Host Disease. *Journal of clinical neuromuscular disease.* 2002; 3(3):136-137.
65. Campos A, Vaz CP, Campilho F, et al. Central nervous system (CNS) tuberculosis following allogeneic stem cell transplantation. *Bone marrow transplantation.* 2000; 25(5):567-569.
66. Camus V, Bouwyn JP, Chamseddine A, et al. Human herpesvirus-6 acute limbic encephalitis after unrelated umbilical cord blood transplantation successfully treated with ganciclovir. *Bone marrow transplantation.* 2015; 50(10):1385-1387.
67. Candoni A, Simeone E, Buttignol S, et al. Late onset cytomegalovirus encephalitis after reduced-intensity conditioning allogeneic SCT: an emerging neurological complication? *Bone marrow transplantation.* 2011; 46(3):455-456.
68. Cavattoni I, Ayuk F, Zander AR, et al. Diagnosis of Toxoplasma gondii infection after allogeneic stem cell transplant can be difficult and requires intensive scrutiny. *Leukemia & lymphoma.* 2010; 51(8):1530-1535.
69. Capoferri AS, M.; Redd, A.; Cash, A.; Xu, D.; Porcella, S. F.; Quinn, T.; Siliciano, R. F.; Levis, M.; Ambinder, R. F.; Durand, C. M. HIV rebound and meningoencephalitis following ART interruption after allogeneic hematopoietic stem cell transplant: An investigation of the source of HIV rebound. *Journal of the International AIDS Society.* 2015; 18:90-91.
70. Carter CW, A. Central diabetes insipidus: An uncommon complication of invasive rhino-cerebral zygomycosis. *Journal of Investigative Medicine.* 2014; 62:568.
71. Castellano-Sanchez AA, Li S, Qian J, Lagoo A, Weir E, Brat DJ. Primary central nervous system posttransplant lymphoproliferative disorders. *Am J Clin Pathol.* 2004; 121(2):246-253.
72. Cavrini F, Stanzani M, Liguori G, Sambri V. Identification of an invasive infection of R. oryzae in a haematological patient using a molecular technique. *Mycoses.* 2010; 53(3):269-271.
73. Ceccanti M, Sbardella E, Letteri F, et al. Acute Flaccid Paralysis by Enterovirus D68 Infection: First Italian Description in Adult Patient and Role of Electrophysiology. *Frontiers in neurology.* 2017; 8:638.
74. Chapuis A, Chabrot C, Mirand A, Poirier P, Nourrisson C. Encephalitis caused by an unusual human herpes virus type 6 and Toxoplasma gondii co-infection in a cord blood transplant recipient. *International journal of infectious diseases : IJID : official publication of the International Society for Infectious Diseases.* 2016; 46:79-81.
75. Chan T, Shannon PT, Detsky ME. An unexpected but underestimated case of disseminated toxoplasmosis. *Transplant Infectious Disease.* 2018; 20(1).
76. Chittick P, Williamson JC, Ohl CA. BK virus encephalitis: case report, review of the literature, and description of a novel treatment modality. *The Annals of pharmacotherapy.* 2013; 47(9):1229-1233.
77. Couriel DR, Beguelin GZ, Giralt S, et al. Chronic graft-versus-host disease manifesting as polymyositis: an uncommon presentation. *Bone marrow transplantation.* 2002; 30(8):543-546.
78. Cho SF, Liu TC, Chang CS. Isolated central nervous system relapse presenting as myeloid sarcoma of acute myeloid leukemia after allogeneic peripheral blood stem cell transplantation. *Annals of hematology.* 2013; 92(1):133-135.
79. Chordia P, Chandrasekar P. Status Epilepticus due to Severe HHV-6 Encephalitis in an Allogeneic Stem Cell Transplant Recipient. *Mediterranean journal of hematology and infectious diseases.* 2014; 6(1):e2014008.
80. Chowdhary S, Chamberlain M. A progressive neurologic disorder with multiple CNS lesions: a neuroimaging clinicopathologic correlation. Progressive multifocal leukoencephalopathy (PML). *Journal of neuroimaging : official journal of the American Society of Neuroimaging.* 2008; 18(3):340-344.
81. Claveau JS, LeBlanc R, Ahmad I, et al. Cerebral adenovirus endotheliitis presenting as posterior reversible encephalopathy syndrome after allogeneic stem cell transplantation. *Bone marrow transplantation.* 2017; 52(10):1457-1459.
82. Choi YJ, Park SJ, Kim JS, Kang EJ, Choi CW, Kim BS. Wernicke's encephalopathy following allogeneic hematopoietic stem cell transplantation. *The Korean journal of hematology.* 2010; 45(4):279-281.
83. Carobolante FC, C.; Ferreri, A.; Lisa Battista, M.; Fanin, R.; Patriarca, F. Posterior reversible encephalopathy syndrome after allogeneic hematopoietic stem cell transplantation in a patient with chronic myeloid leukemia. *Bone marrow transplantation.* 2014; 49.
84. Carrion R, Serrano D, Buno I, et al. Post-transplant lymphoproliferative disorder mimicking a thrombotic microangiopathy. *Biology of blood and marrow transplantation : journal of the American Society for Blood and Marrow Transplantation.* 2006; 12(11):1203-1205.
85. Carroll CB, Hilton DA, Hamon M, Zajicek JP. Muscle cramps and weakness secondary to graft versus host disease fasciitis. *European journal of neurology.* 2005; 12(4):320-322.
86. Cocito D, Romagnolo A, Rosso M, Peci E, Lopiano L, Merola A. CIDP-like neuropathies in graft versus host disease. *Journal of the peripheral nervous system : JPNS.* 2015; 20(1):1-6.
87. Collins MP, Periquet MI. Vasculitic neuropathy in chronic graft-versus-host disease (GVHD). *J Neurol Sci.* 2000; 175(1):71-73.

88. Colombo AAP, C.; Marchioni, E.; Ripamonti, F.; Di Matteo, A.; Baldanti, F.; Furione, M.; Alessandrino, E. P. T depletion is associated to an higher risks of encephalitis in adults patients receiving allogeneic haemopoietic stem cell transplantation. *Bone marrow transplantation.* 2012; 47.
89. Cordonnier C, Feuilhade F, Vernant JP, Marsault C, Rodet M, Rochant H. Cytomegalovirus encephalitis occurring after bone marrow transplantation. *Scandinavian journal of haematology.* 1983; 31(3):248-252.
90. Curran OEA-S, S.; Pomplun, S. Central nervous system recurrence of acute myeloid leukaemia after allogeneic haematopoietic stem cell transplantation. *Neuropathology and applied neurobiology.* 2013(39):47-48.
91. Curran OEW, R.; Bodi, I. A fatal case of transverse myelitis associated with Cytomegalovirus reactivation in a bone marrow transplant recipient. *Clinical Neuropathology.* 2012; 31(4).
92. Davis D, Henslee PJ, Markesbery WR. Fatal adenovirus meningoencephalitis in a bone marrow transplant patient. *Ann Neurol.* 1988; 23(4):385-389.
93. Davoudi S, Anderlini P, Fuller GN, Kontoyiannis DP. A long-term survivor of disseminated Aspergillus and mucorales infection: an instructive case. *Mycopathologia.* 2014; 178(5-6):465-470.
94. U. DRNGMAGBSGMMGS. Case report of miliar tuberculosis of lung, tongue and brain after allogeneic stem cell transplantation. *Onkologie.* 2011; 34.
95. de Beukelaar JW, van Arkel C, van den Bent MJ, et al. Resolution of EBV(+) CNS lymphoma with appearance of CSF EBV-specific T cells. *Ann Neurol.* 2005; 58(5):788-792.
96. de Souza Franceschi FL, Green J, Cayci Z, et al. Human herpesvirus 6 is associated with status epilepticus and hyponatremia after umbilical cord blood transplantation. *The Canadian journal of infectious diseases & medical microbiology = Journal canadien des maladies infectieuses et de la microbiologie medicale.* 2014; 25(3):170-172.
97. Debaere C, Stadnik T, De Maeseneer M, Osteaux M. Diffusion-weighted MRI in cyclosporin A neurotoxicity for the classification of cerebral edema. *European radiology.* 1999; 9(9):1916-1918.
98. Dehghani M, Davarpanah MA. Epididymo-orchitis and central nervous system nocardiosis in a bone marrow transplant recipient for acute lymphoblastic leukemia. *Experimental and clinical transplantation : official journal of the Middle East Society for Organ Transplantation.* 2009; 7(4):264-266.
99. Devinsky O, Lemann W, Evans AC, Moeller JR, Rottenberg DA. Akinetic mutism in a bone marrow transplant recipient following total-body irradiation and amphotericin B chemoprophylaxis. A positron emission tomographic and neuropathologic study. *Archives of neurology.* 1987; 44(4):414-417.
100. Abstracts of the 22nd European Congress of Pathology. September 4-9, 2009. Florence, Italy. *Virchows Archiv : an international journal of pathology.* 2009; 455 Suppl 1:S1-448.
101. Diamanti L, Franciotta D, Berzero G, et al. Late post-transplant anti-aquaporin-4 Ab-positive optic neuritis in a patient with AML. *Bone marrow transplantation.* 2015; 50(8):1125-1126.
102. Doi Y, Sugahara H, Yamamoto K, Uji-ie H, Kakimoto T, Sakoda H. Immune-mediated peripheral neuropathy occurring simultaneously with recurrent graft-versus-host disease after allogenic hematopoietic stem cell transplantation. *Leuk Res.* 2012; 36(4):e63-65.
103. Transplant-Associated Thrombotic Microangiopathy (TA-TMA) in a patient with acute GVHD after allogeneic hematopoietic stem cell transplantation successfully treated with eculizumab. *Bone marrow transplantation.* 2016; 51 Suppl 1:S564-609.
104. Dowell JE, Moots PL, Stein RS. Myasthenia gravis after allogeneic bone marrow transplantation for lymphoblastic lymphoma. *Bone marrow transplantation.* 1999; 24(12):1359-1361.
105. Edwards LL, Wszolek ZK, Normand MM. Neurophysiologic evaluation of cyclosporine toxicity associated with bone marrow transplantation. *Acta neurologica Scandinavica.* 1996; 94(5):358-364.
106. El-Cheikh J, Furst S, Casalonga F, et al. JC Virus Leuko-Encephalopathy in Reduced Intensity Conditioning Cord Blood Transplant Recipient with a Review of the Literature. *Mediterranean journal of hematology and infectious diseases.* 2012; 4(1):e2012043.
107. El-Jawahri AR, Schaefer PW, El Khoury JB, Martinez-Lage M. Case 5-2018: A 63-year-old man with confusion after stem-cell transplantation. *New England Journal of Medicine.* 2018; 378(7):659-669.
108. Enriquez-Marulanda A, Sierra-Ruiz M, Jaramillo FJ, et al. Hemichoreo-hemibalism as a Manifestation of Central Nervous System Chronic Graft-versus-Host Disease. *Movement disorders clinical practice.* 2017; 4(4):495-498.
109. Escuissato DL, de Aguiar RO, Gasparetto EL, Muller NL. Disseminated toxoplasmosis after bone marrow transplantation: high-resolution CT appearance. *Journal of thoracic imaging.* 2004; 19(3):207-209.
110. A rare case of chronic graft verses host disease with central nervous system involvement. *The American journal of gastroenterology.* 2015; 110 Suppl 1:S40-S550.
111. Farid S, AbuSaleh O, Liesman R, Sohail MR. Isolated cerebral mucormycosis caused by Rhizomucor pusillus. *BMJ case reports.* 2017; 2017.
112. Fonseca M, Fonseca AC. Cerebral microbleeds in graft-versus-host disease: A case report. *Journal of the Neurological Sciences.* 2017; 381:503-504.
113. Foolad F, Kontoyiannis DP. Persistent CNS toxicity in a patient receiving posaconazole tablets after discontinuation of voriconazole due to supratherapeutic serum levels. *Journal of Antimicrobial Chemotherapy.* 2018; 73(1):256-258.
114. Forest F, Duband S, Pillet S, et al. Lethal human herpesvirus-6 encephalitis after cord blood transplant. *Transplant infectious disease : an official journal of the Transplantation Society.* 2011; 13(6):646-649.
115. Fotheringham J, Akhyani N, Vortmeyer A, et al. Detection of active human herpesvirus-6 infection in the brain: correlation with polymerase chain reaction detection in cerebrospinal fluid. *The Journal of infectious diseases.* 2007; 195(3):450-454.
116. Fuchs M, Reinhofer M, Ragoschke-Schumm A, et al. Isolated central nervous system relapse of chronic myeloid leukemia after allogeneic hematopoietic stem cell transplantation. *BMC blood disorders.* 2012; 12:9.
117. Fitzgerald SA. Human herpesvirus 6-associated limbic encephalitis refractory to therapeutic plasma exchange in adult recipient of unrelated umbilical cord blood transplantation. *Journal of clinical apheresis.* 2009; 24(2):89-90.
118. Freitas T. Posterior reversible encephalopathy syndrome (PRES) associated to sirolimus administration: A case report. *Bone marrow transplantation.* 2014; 49:S479.
119. Fujii N, Ikeda K, Koyama M, et al. Calcineurin inhibitor-induced irreversible neuropathic pain after allogeneic hematopoietic stem cell transplantation. *International journal of hematology.* 2006; 83(5):459-461.
120. Fujisaki G, Kami M, Murashige N, et al. Guillain-Barre syndrome associated with rapid immune reconstitution following allogeneic hematopoietic stem cell transplantation. *Bone marrow transplantation.* 2006; 37(6):617-619.
121. Fujiwara S, Muroi K, Tatara R, et al. Intrathecal administration of high-titer cytomegalovirus immunoglobulin for cytomegalovirus meningitis. *Case reports in hematology.* 2014; 2014:272458.
122. Fukatsu M, Murakami T, Ohkawara H, et al. A possible role of low regulatory T cells in anti-acetylcholine receptor antibody positive myasthenia gravis after bone marrow transplantation. *BMC neurology.* 2017; 17(1):93.
123. Fukuno K, Tomonari A, Takahashi S, et al. Varicella-zoster virus encephalitis in a patient undergoing unrelated cord blood transplantation for myelodysplastic syndrome-overt leukemia. *International journal of hematology.* 2006; 84(1):79-82.
124. Golla H, Thier P. Ocular Flutter—A Sign of Brain-Stem Pathology as Rare Consequence of Cyclosporin A Treatment. *Neuro-Ophthalmology*. 2005; 29(2):81-84.
125. Gabriel CM, Goldman JM, Lucas S, Hughes RA. Vasculitic neuropathy in association with chronic graft-versus-host disease. *J Neurol Sci.* 1999; 168(1):68-70.
126. Ganapule AP, Varghese SS, Chacko G, Aparna I, Viswabandya A. Glioblastoma Multiforme in a Post Allogeneic Stem Cell Transplant Patient. A Case Report and Literature Review of Post Transplant Neurological Tumors. *Indian journal of hematology & blood transfusion : an official journal of Indian Society of Hematology and Blood Transfusion.* 2016; 32(Suppl 1):192-195.
127. Gatti-Mays ME, Manion M, Bowen LN, et al. Toxoplasmosis encephalitis with immune-reconstitution inflammatory syndrome in an allogeneic stem cell transplant patient: a case report. *Bone marrow transplantation.* 2016; 51(12):1622-1624.

128. Gauthier J, Castagna L, Garnier F, et al. Reduced-intensity and non-myeloablative allogeneic stem cell transplantation from alternative HLA-mismatched donors for Hodgkin lymphoma: a study by the French Society of Bone Marrow Transplantation and Cellular Therapy. *Bone marrow transplantation.* 2017; 52(5):689-696.
129. Ghabrial R, Ananda A, van Hal SJ, et al. Invasive Fungal Sinusitis Presenting as Acute Posterior Ischemic Optic Neuropathy. *Neuro-ophthalmology (Aeolus Press).* 2018; 42(4):209-214.
130. Gocmen R, Colpak AI, Goker H, Kaya AA, Onder H. Reversible diffusion restriction of optic radiations: A distinct form of cyclosporine induced leukoencephalopathy presenting with visual loss. *J Neurol Sci.* 2016; 366:155-157.
131. Gong J, Li J, Liang H. Extramedullary relapse presenting as trigeminal neuralgia and diplopia after allogeneic hematopoietic stem cell transplantation. *Internal medicine (Tokyo, Japan).* 2011; 50(10):1117-1119.
132. Gonzalez MI, Caballero D, Lopez C, et al. Cerebral toxoplasmosis and Guillain-Barre syndrome after allogeneic peripheral stem cell transplantation. *Transplant infectious disease : an official journal of the Transplantation Society.* 2000; 2(3):145-149.
133. Gonzalez Vicent M, Molina B, Fabregat M, Segura M, Diaz MA. Toxoplasmosis and secondary Guillain-Barré associated with ruxolitinib as graft-versus-host disease treatment. *Pediatric Blood and Cancer.* 2019; 66(1).
134. Gopal AK, Thorning DR, Back AL. Fatal outcome due to cyclosporine neurotoxicity with associated pathological findings. *Bone marrow transplantation.* 1999; 23(2):191-193.
135. Gorniak RJ, Young GS, Wiese DE, Marty FM, Schwartz RB. MR imaging of human herpesvirus-6-associated encephalitis in 4 patients with anterograde amnesia after allogeneic hematopoietic stem-cell transplantation. *AJNR. American journal of neuroradiology.* 2006; 27(4):887-891.
136. Giovannelli F, Basagni B, Potenza L, Foschi V, De Tanti A. Long-term cognitive sequelae in a case of Wernicke's encephalopathy after allogeneic stem cell transplantation. *Neurocase.* 2016; 22(2):187-190.
137. Graetz R, Meyer R, Shehab K, Katsanis E. Successful resolution of hyperammonemia following hematopoietic cell transplantation with directed treatment of Ureaplasma parvum infection. *Transplant Infectious Disease.* 2018; 20(2).
138. Gruhn B, Meerbach A, Egerer R, et al. Successful treatment of Epstein-Barr virus-induced transverse myelitis with ganciclovir and cytomegalovirus hyperimmune globulin following unrelated bone marrow transplantation. *Bone marrow transplantation.* 1999; 24(12):1355-1358.
139. Greenspan A, Deeg HJ, Cottler-Fox M, Sirdofski M, Spitzer TR, Kattah J. Incapacitating peripheral neuropathy as a manifestation of chronic graft-versus-host disease. *Bone marrow transplantation.* 1990; 5(5):349-352.
140. Gupta V, Keller A, Halliday W, et al. Cavernous sinus thrombosis presenting with diplopia in an allogeneic bone marrow transplant recipient. *American journal of hematology.* 2004; 77(1):77-81.
141. Haase R, Sauer H, Dagwadordsch U, Foell J, Lieser U. Successful treatment of Bacillus cereus meningitis following allogenic stem cell transplantation. *Pediatric transplantation.* 2005; 9(3):338-341.
142. Hackanson B, Zeiser R, Bley TA, et al. Fatal varicella zoster virus encephalitis in two patients following allogeneic hematopoietic stem cell transplantation. *Clinical transplantation.* 2005; 19(4):566-570.
143. Hamadani M, Martin LK, Benson DM, Copelan EA, Devine SM, Hofmeister CC. Central nervous system post-transplant lymphoproliferative disorder despite negative serum and spinal fluid Epstein-Barr virus DNA PCR. *Bone marrow transplantation.* 2007; 39(4):249-251.
144. Hamprecht K, Eckle T, Prix L, Faul C, Einsele H, Jahn G. Ganciclovir-resistant cytomegalovirus disease after allogeneic stem cell transplantation: pitfalls of phenotypic diagnosis by in vitro selection of an UL97 mutant strain. *The Journal of infectious diseases.* 2003; 187(1):139-143.
145. Hanel M, Fiedler F, Thorns C. Anti-CD20 monoclonal antibody (Rituximab) and Cidofovir as successful treatment of an EBV-associated lymphoma with CNS involvement. *Onkologie.* 2001; 24(5):491-494.
146. M H. Epileptic state and hippocampal sclerosis following allogeneic stem cell transplantation with reduced intensity conditioning in a high-risk lymphoma Patient-A case report. *Onkologie* 2013; 36(167).
147. Haruki H, Koga M, Ogasawara J, Omoto M, Kawai M, Kanda T. Neuropathy in chronic graft-versus-host disease caused by donor T cells. *Muscle & nerve.* 2012; 46(4):610-611.
148. Harvey CJ, Peniket AJ, Miszkiel K, et al. MR angiographic diagnosis of cerebral venous sinus thrombosis following allogeneic bone marrow transplantation. *Bone marrow transplantation.* 2000; 25(7):791-795.
149. Harvey CM, Gottipati R, Schwarz S, et al. Acute disseminated encephalomyelitis following allo-SCT: central nervous system manifestation of GVHD. *Bone marrow transplantation.* 2014; 49(6):854-856.
150. Hatsuta H. 70-year-old female cases of encephalomyelitis after cord blood transplantation. *Neuropathology : official journal of the Japanese Society of Neuropathology.* 2013; 33(3):387.
151. Hattori N, Yamamoto K, Kawaguchi Y, et al. Early relapse of severe chronic active Epstein-Barr virus infection with posterior reversible encephalopathy syndrome after reduced intensity stem cell transplantation with umbilical cord blood. *Leukemia & lymphoma.* 2016; 57(10):2448-2451.
152. Heidarzadeh Z, Mousavi SA, Ostovan VR, Nafissi S. Muscle-specific kinase antibody associated myasthenia gravis after bone marrow transplantation. *Neuromuscular disorders : NMD.* 2014; 24(2):148-150.
153. A H. Encephalitis caused by a novel adenovirus type of species D in an adult allogenic SCT recipient. *Bone marrow transplantation.* 2016; 51:S191-S192.
154. Helton KJ, Maron G, Mamcarz E, Leventaki V, Patay Z, Sadighi Z. Unusual magnetic resonance imaging presentation of post-BMT cerebral toxoplasmosis masquerading as meningoencephalitis and ventriculitis. *Bone marrow transplantation.* 2016; 51(11):1533-1536.
155. Hernandez-Boluda JC, Lis MJ, Goterris R, et al. Guillain-Barre syndrome associated with cytomegalovirus infection after allogeneic hematopoietic stem cell transplantation. *Transplant infectious disease : an official journal of the Transplantation Society.* 2005; 7(2):93-96.
156. I H. Efficacy of complement modulating therapies in allogeneic stem cell transplantation associated thrombotic microangiopathy. *Haematologica.* 2015(100):798.
157. Heurkens AH, Koelma IA, de Planque MM, Polderman AM, van der Meer JW. Failure to diagnose fatal disseminated toxoplasmosis in a bone marrow transplant recipient: the possible significance of declining antibody titres. *The Journal of infection.* 1989; 18(3):283-288.
158. Hiatt B, DesJardin L, Carter T, Gingrich R, Thompson C, de Magalhaes-Silverman M. A fatal case of West Nile virus infection in a bone marrow transplant recipient. *Clinical infectious diseases : an official publication of the Infectious Diseases Society of America.* 2003; 37(9):e129-131.
159. Hill KS, Hurlock CA, Jarvis L, et al. Muscle-specific kinase antibody associated myasthenia gravis post allogeneic stem cell transplantation-successful treatment with rituximab and plasma exchange alone. *Bone marrow transplantation.* 2017; 52:507.
160. Hino Y, Doki N, Sekiya N, Takaki Y, Ohashi K. Optic neuritis as an initial manifestation of human herpesvirus 6 reactivation after unrelated bone marrow transplantation. *British journal of haematology.* 2016; 172(5):654.
161. Hino Y, Doki N, Senoo Y, et al. Disseminated nocardiosis after unrelated bone marrow transplantation. *Transplant infectious disease : an official journal of the Transplantation Society.* 2016; 18(6):942-945.
162. Hirst LW, Clark AW, Wolinsky JS, et al. Downbeat nystagmus. A case report of herpetic brain stem encephalitis. *Journal of clinical neuro-ophthalmology.* 1983; 3(4):245-249.
163. Hoefnagels WA, Gerritsen EJ, Brouwer OF, Souverijn JH. Cyclosporin encephalopathy associated with fat embolism induced by the drug's solvent. *Lancet.* 1988; 2(8616):901.
164. Hon C, Au WY, Cheng VC. Ophthalmic zoster sine herpete presenting as oculomotor palsy after marrow transplantation for acute myeloid leukemia. *Haematologica.* 2005; 90(12 Suppl):EIM04.
165. Hong DS, Jacobson KL, Raad, II, et al. West Nile encephalitis in 2 hematopoietic stem cell transplant recipients: case series and literature review. *Clinical infectious diseases : an official publication of the Infectious Diseases Society of America.* 2003; 37(8):1044-1049.
166. Hossain MA, Jehangir W, Nai Q, et al. Posterior Reversible Encephalopathy Syndrome in a Bone Marrow Transplant Patient: A Complication of Immunosuppressive Drugs? *World journal of oncology.* 2015; 6(4):426-428.

167. Hsiao HH, Huang HL, Wang HC, et al. Acute cerebral infarct with elevated factor VIII level during the thrombocytopenic stage after hematopoietic stem cell transplant. *Experimental and clinical transplantation : official journal of the Middle East Society for Organ Transplantation.* 2014; 12(2):171-172.
168. Hubele F, Bilger K, Kremer S, Imperiale A, Lioure B, Namer IJ. Sequential FDG PET and MRI findings in a case of human herpes virus 6 limbic encephalitis. *Clin Nucl Med.* 2012; 37(7):716-717.
169. Ikegawa S, Fujii N, Tadokoro K, et al. Progressive multifocal leukoencephalopathy after T-cell replete HLA-haploidentical transplantation with post-transplantation cyclophosphamide graft-versus-host disease prophylaxis. *Transplant infectious disease : an official journal of the Transplantation Society.* 2018; 20(2):e12850.
170. Ikegame K, Kato R, Fujioka T, et al. Detection of donor-derived CMV-specific T cells in cerebrospinal fluid in a case of CMV meningoencephalitis after cord blood stem cell transplantation. *International journal of hematology.* 2013; 97(2):287-290.
171. Ibrahim U, Saqib A, Mohammad F, Terjanian T. An Unusual Presentation of Nocardiosis in an Allogeneic Transplant Recipient. *Cureus.* 2016; 8(10):e834.
172. Im J-H, Park IS, Kim EY, et al. Disseminated Invasive Aspergillosis with Multiple Brain Abscess after Allogeneic Hematopoietic Stem Cell Transplantation Treated Successfully with Voriconazole and Neurosurgical Intervention. *Infect Chemother.* 2012; 44(5):395-398.
173. Imataki O, Uemura M. Ganciclovir-resistant HHV-6 encephalitis that progressed rapidly after bone marrow transplantation. *Journal of clinical virology : the official publication of the Pan American Society for Clinical Virology.* 2015; 69:176-178.
174. Imataki O, Uemura M, Shintani T, Matsumoto K. Reversible cerebral vasoconstriction syndrome resulted in cerebral infarction after allogeneic stem cell transplantation: a case report. *Annals of hematology.* 2014; 93(5):895-896.
175. Ionita C, Wasay M, Balos L, Bakshi R. MR imaging in toxoplasmosis encephalitis after bone marrow transplantation: paucity of enhancement despite fulminant disease. *AJNR. American journal of neuroradiology.* 2004; 25(2):270-273.
176. Jehn U, Fink M, Gundlach P, et al. Lethal cardiac and cerebral toxoplasmosis in a patient with acute myeloid leukemia after successful allogeneic bone marrow transplantation. *Transplantation.* 1984; 38(4):430-433.
177. Jennane S, Mahtat el M, Konopacki J, et al. Cyclosporine-related posterior reversible encephalopathy syndrome after cord blood stem cell transplantation. *Hematology/oncology and stem cell therapy.* 2013; 6(2):71.
178. Johnson H, E MB, Sharp SE. A 53-year-old stem cell transplant recipient with meningitis and bacteremia. *J Clin Microbiol.* 2011; 49(12):4031, 4421.
179. Kamble RT, Chang CC, Sanchez S, Carrum G. Central nervous system graft-versus-host disease: report of two cases and literature review. *Bone marrow transplantation.* 2007; 39(1):49-52.
180. Kamble RT, Scholoff A, Obi AG, Heslop HE, Brenner MK, Carrum G. Neuropathic dermatomes and cutaneous ulceration in patients with chronic GVHD. *Bone marrow transplantation.* 2014; 49(7):986-987.
181. Kanamori H, Fujisawa S, Yamaji S, et al. Posttransplantation Epstein-Barr viral meningitis in a patient with chronic myelogenous leukemia. *International journal of hematology.* 2001; 74(4):473-474.
182. Kanter D. Relapsing remitting polyradiculoneuropathy associated with graft-versus-host disease after peripheral blood stem cell transplant for acute myelogenous leukemia: A case report. *PM and R* 2011; 3(10):S270-S271.
183. Kapp M, Schargus M, Deuchert T, et al. Endophthalmitis as primary clinical manifestation of fatal fusariosis in an allogeneic stem cell recipient. *Transplant infectious disease : an official journal of the Transplantation Society.* 2011; 13(4):374-379.
184. Kaufman GP, Aksamit AJ, Klein CJ, Yi ES, Delone DR, Litzow MR. Progressive multifocal leukoencephalopathy: a rare infectious complication following allogeneic hematopoietic cell transplantation (HCT). *European journal of haematology.* 2014; 92(1):83-87.
185. Kaushik S, Flagg E, Wise CM, Hadfield G, McCarty JM. Granulomatous myositis: a manifestation of chronic graft-versus-host disease. *Skeletal radiology.* 2002; 31(4):226-229.
186. Kittan NA, Beier F, Kurz K, et al. Isolated cerebral manifestation of Epstein-Barr virus-associated post-transplant lymphoproliferative disorder after allogeneic hematopoietic stem cell transplantation: a case of clinical and diagnostic challenges. *Transplant infectious disease : an official journal of the Transplantation Society.* 2011; 13(5):524-530.
187. Kawano Y, Miyazaki T, Watanabe T, et al. HLA-mismatched CD34-selected stem cell transplant complicated by HHV-6 reactivation in the central nervous system. *Bone marrow transplantation.* 2000; 25(7):787-790.
188. Kawahara Y, Wada S, Nijima H, et al. Rhinocerebral Mucormycosis with Temporal Artery Thrombosis in an Adolescent Following HLA-haploidentical Stem Cell Transplantation. *Journal of pediatric hematology/oncology.* 2018; 40(7):e461-e463.
189. Kawaguchi T, Takeuchi M, Kawajiri C, et al. Severe hyponatremia caused by syndrome of inappropriate secretion of antidiuretic hormone developed as initial manifestation of human herpesvirus-6-associated acute limbic encephalitis after unrelated bone marrow transplantation. *Transplant infectious disease : an official journal of the Transplantation Society.* 2013; 15(2):E54-57.
190. Kawamata T, Ohno N, Sato K, et al. A case of post-transplant adult T-cell leukemia/lymphoma presenting myelopathy similar to but distinct from human T-cell leukemia virus type I (HTLV- I)-associated myelopathy. *SpringerPlus.* 2014; 3:581.
191. Kearney S, Fulton AJ, McMullin MF, McKenna E. POC07 Central nervous graft-vs-host disease causing intracranial vasculopathy. *Journal of Neurology, Neurosurgery & Psychiatry.* 2010; 81(11):e36-e36.
192. Kelly P, Staunton H, Lawler M, et al. Multifocal remitting-relapsing cerebral demyelination twenty years following allogeneic bone marrow transplantation. *Journal of neuropathology and experimental neurology.* 1996; 55(9):992-998.
193. Kew AK, Macaulay R, Burrell S, Rubin S, Dow G, Couban S. Central nervous system graft-versus-host disease presenting with granulomatous encephalitis. *Bone marrow transplantation.* 2007; 40(2):183-184.
194. Khalaf AM, Hashim MA, Alsharabati M, et al. Late-Onset Cerebral Toxoplasmosis After Allogeneic Hematopoietic Stem Cell Transplantation. *The American journal of case reports.* 2017; 18:246-250.
195. Khan M, Ubogu E, Alsharabati M, Salzman D, Mineishi S, Saad A. Acute myofascitis as a manifestation of chronic graft-versus-host disease. *Muscle & nerve.* 2016; 53(2):327-329.
196. Kharfan-Dabaja MA, Ayala E, Greene J, Rojiani A, Murtagh FR, Anasetti C. Two cases of progressive multifocal leukoencephalopathy after allogeneic hematopoietic cell transplantation and a review of the literature. *Bone marrow transplantation.* 2007; 39(2):101-107.
197. Khoury H, Adkins D, Brown R, et al. Successful treatment of cerebral toxoplasmosis in a marrow transplant recipient: contribution of a PCR test in diagnosis and early detection. *Bone marrow transplantation.* 1999; 23(4):409-411.
198. Kida A, Ohashi K, Kobayashii T, et al. Incapacitating lower limb pain syndrome in cord blood stem cell transplant recipients with calcineurin inhibitor. *Pathology oncology research : POR.* 2004; 10(4):204-206.
199. Kim YJ, Kim DW, Lee DG, et al. Human herpesvirus-6 as a possible cause of encephalitis and hemorrhagic cystitis after allogeneic hematopoietic stem cell transplantation. *Leukemia.* 2002; 16(5):958-959.
200. Kleiter I, Poeschl P, Kraus PD, et al. Entrapment syndrome of multiple nerves in graft-versus-host disease. *Muscle & nerve.* 2014; 49(1):138-142.
201. Kishida S, Tanaka K. Mefloquine treatment in a patient suffering from progressive multifocal leukoencephalopathy after umbilical cord blood transplant. *Internal medicine (Tokyo, Japan).* 2010; 49(22):2509-2513.
202. Koide T, Yamada M, Takahashi T, et al. Cyclosporine A-associated fatal central nervous system angiopathy in a bone marrow transplant recipient: an autopsy case. *Acta neuropathologica.* 2000; 99(6):680-684.
203. Kondo T, Tasaka T, Matsumoto K, et al. Philadelphia chromosome-positive acute lymphoblastic leukemia with extramedullary and meningeal relapse after allogeneic hematopoietic stem cell transplantation that was successfully treated with dasatinib. *SpringerPlus.* 2014; 3:177.

204. Kremer S, Matern JF, Bilger K, et al. EBV limbic encephalitis after allogenic hematopoietic stem cell transplantation. *Journal of neuroradiology. Journal de neuroradiologie.* 2010; 37(3):189-191.
205. Kuroshima K, Tsuchida T, Sato C, et al. Clinical importance of changes in MRI during early stage of human herpesvirus-6 encephalitis after hematopoietic stem cell transplantation. *Journal of the Neurological Sciences.* 2017; 381:423.
206. Kural C, Ozer MI, Ezgu MC, et al. Intracavitary amphotericin B in the treatment of intracranial aspergillosis. *Journal of Clinical Neuroscience.* 2018; 51:75-79.
207. Lacerda JF, Martins C, Carmo JA, et al. Invasive aspergillosis of the central nervous system after allogeneic stem cell transplantation. *The Journal of infection.* 2005; 51(3):e191-194.
208. Lawson BO, Seth H, Quan D. Phenytoin and Rifampin Do Not Decrease Levels in Acute Tacrolimus Toxicity. *Journal of investigative medicine high impact case reports.* 2018; 6.
209. R. LC. Implication of cyclosporine in the development of reversible posterior encephalopathy syndrome in three patients with allogenic bone marrow transplants: Is MRI apparent diffusion coefficient of prognostic and therapeutic value? *Bone marrow transplantation.* 2014; 49:S471-S472.
210. Lieberman F, Yazbeck V, Raptis A, Felgar R, Boyiadzis M. Primary central nervous system post-transplant lymphoproliferative disorders following allogeneic hematopoietic stem cell transplantation. *Journal of neuro-oncology.* 2012; 107(2):225-232.
211. Liguori R, Vincent A, Avoni P, et al. Acquired neuromyotonia after bone marrow transplantation. *Neurology.* 2000; 54(6):1390-1391.
212. Liu FC, Chen CH, Chao TY. Polymyositis complicating donor lymphocyte infusion after stem cell transplantation for relapsed chronic myeloid leukemia: report of a case and review of literature. *Clinical rheumatology.* 2007; 26(7):1207-1210.
213. Lee B. Encephalopathy after bone marrow transplantation. *The Pediatric infectious disease journal.* 2005; 24(1):83-84, 94-85.
214. Liapis K, Manaka K, Baltadakis I, Karakasis D. Cerebral aspergillosis. *European journal of haematology.* 2009; 82(4):327-328.
215. Lim KH, Kim S, Lee YS, et al. Central pontine myelinolysis in a patient with acute lymphoblastic leukemia after hematopoietic stem cell transplantation: a case report. *Journal of Korean medical science.* 2008; 23(2):324-327.
216. Lind MJ, McWilliam L, Jip J, Scarffe JH, Morgenstern GR, Chang J. Cyclosporin associated demyelination following allogeneic bone marrow transplantation. *Hematological oncology.* 1989; 7(1):49-52.
217. Liu WH, Chen WT, Fang LH, Chen RL. Idiopathic Pneumonia Syndrome and Thrombotic Microangiopathy Following Nonmyeloablative Haploidentical Peripheral Blood Stem Cell Transplantation and Posttransplant Cyclophosphamide: A Case Report. *Medicine.* 2015; 94(29):e1200.
218. Long SG, Leyland MJ, Milligan DW. Listeria meningitis after bone marrow transplantation. *Bone marrow transplantation.* 1993; 12(5):537-539.
219. Lopes da Silva R, Ferreira I, Teixeira G, et al. BK virus encephalitis with thrombotic microangiopathy in an allogeneic hematopoietic stem cell transplant recipient. *Transplant infectious disease : an official journal of the Transplantation Society.* 2011; 13(2):161-167.
220. Lee HC, Mulanovich V, Nieto Y. Progressive multifocal leukoencephalopathy after allogeneic bone marrow transplantation for acute myeloid leukemia. *Journal of the National Comprehensive Cancer Network : JNCCN.* 2014; 12(12):1660-1664; quiz 1664.
221. Lee SH, Kim BC, Yang DH, et al. Calcineurin inhibitor-mediated bilateral hippocampal injury after bone marrow transplantation. *Journal of neurology.* 2008; 255(6):929-931.
222. Lee SY, Lee SR, Kim DS, Choi CW, Kim BS, Park Y. BK virus encephalitis without concurrent hemorrhagic cystitis in an allogeneic hematopoietic stem cell transplant recipient. *Blood Res.* 2013; 48(3):226-228.
223. Lee S, Kim SH, Choi SM, et al. Cytomegalovirus ventriculoencephalitis after unrelated double cord blood stem cell transplantation with an alemtuzumab-containing preparative regimen for Philadelphia-positive acute lymphoblastic leukemia. *Journal of Korean medical science.* 2010; 25(4):630-633.
224. Lee SY, Kim SH, Ha SG. Bilateral Optic Nerve Involvement Combined with Unilateral Facial Palsy in a Patient with Acute Myeloid Leukaemia: A Case Report. *Neuro-ophthalmology (Aeolus Press).* 2018; 42(2):122-125.
225. Lee SY, Kim SH, Ha SG. Bilateral Optic Nerve Involvement Combined with Unilateral Facial Palsy in a Patient with Acute Myeloid Leukaemia: A Case Report. *Neuro-Ophthalmology.* 2018; 42(2):122-125.
226. Lopez-Jimenez J, Sanchez A, Fernandez CS, Gutierrez C, Herrera P, Odriozola J. Cyclosporine-induced retinal toxic blindness. *Bone marrow transplantation.* 1997; 20(3):243-245.
227. Leano AM, Miller K, White AC. Chronic graft-versus-host disease-related polymyositis as a cause of respiratory failure following allogeneic bone marrow transplant. *Bone marrow transplantation.* 2000; 26(10):1117-1120.
228. Loseto G. A case of posterior reversible encephalophathy syndrome triggered by sun and heat exposure in a patient who underwent allogeneic bone marrow transplantation and with a history of cyclosporine neurotoxicity. *Bone marrow transplantation.* 2012; 47:S348-S350.
229. Lotze C, Schuler F, Kruger WH, et al. Combined immunoradiotherapy induces long-term remission of CNS relapse of peripheral, diffuse, large-cell lymphoma after allogeneic stem cell transplantation: case study. *Neuro-oncology.* 2005; 7(4):508-510.
230. Lowenberg B, van Gijn J, Prins E, Polderman AM. Fatal cerebral toxoplasmosis in a bone marrow transplant recipient with leukemia. *Transplantation.* 1983; 35(1):30-34.
231. Lorenzoni PJ, Scola RH, Carsten AL, et al. Chronic inflammatory demyelinating polyradiculoneuropathy in chronic graft-versus-host disease following allogeneic hematopoietic stem cell transplantation: case report. *Arq Neuropsiquiatr.* 2007; 65(3A):700-704.
232. Lux P, Ringelstein, A., Hünerlitürkoglu, A. et al. Tacrolimus-Induced Encephalopathy in Radiology. *Clin Neuroradiol.* 2007; 17.
233. N M. Highly resistant Candida cerebral infection. *Mycoses.* 2012; 55(204).
234. Madan B, Schey SA. Reversible cortical blindness and convulsions with cyclosporin A toxicity in a patient undergoing allogeneic peripheral stem cell transplantation. *Bone marrow transplantation.* 1997; 20(9):793-795.
235. Mackey JR, Desai S, Larratt L, Cwik V, Nabholtz JM. Myasthenia gravis in association with allogeneic bone marrow transplantation: clinical observations, therapeutic implications and review of literature. *Bone marrow transplantation.* 1997; 19(9):939-942.
236. Mahajan S. Rapidly progressive polyneuropathy from leukimic infiltration mascarading as cervical disc herniation. *Neurology.* 2016; 86(16).
237. Miklavčič P, Avčin S, Jazbec J, et al. Cyclosporine A induced dystonia-parkinsonism. *Journal of the Neurological Sciences.* 2017; 375:68-70.
238. Miyagi T, Itonaga H, Aosai F, et al. Successful treatment of toxoplasmic encephalitis diagnosed early by polymerase chain reaction after allogeneic hematopoietic stem cell transplantation: two case reports and review of the literature. *Transplant infectious disease : an official journal of the Transplantation Society.* 2015; 17(4):593-598.
239. Mohty B. A fatal microascus cinereus (anamorph scopulariopsis) brain abscess in an allogeneic bone marrow transplant recipient. *Haematologica.* 2010; 95:649-650.
240. Mori A, Tanaka J, Kobayashi S, et al. Fatal cerebral hemorrhage associated with cyclosporin-A/FK506-related encephalopathy after allogeneic bone marrow transplantation. *Annals of hematology.* 2000; 79(10):588-592.
241. Machetti M, Zotti M, Veroni L, et al. Antigen detection in the diagnosis and management of a patient with probable cerebral aspergillosis treated with voriconazole. *Transplant infectious disease : an official journal of the Transplantation Society.* 2000; 2(3):140-144.
242. Majeed A, Mushtaq A, Zangeneh T, et al. Intractable cerebral Nocardia mexicana in a GvHD patient successfully treated with linezolid. *Bone marrow transplantation.* 2017; 52(10):1476-1478.

243. Malkan UY, Gunes G, Demiroglu H, Goker H. Immunosuppression-associated posterior reversible encephalopathy syndrome in an acute leukemia case. *Hematology reports.* 2018; 10(4):96-97.
244. Manabe S, Kashii S, Miki Y, Honda Y. Analysis of cyclosporin A-induced reversible cortical blindness by diffusion-weighted magnetic resonance imaging techniques. *Japanese journal of ophthalmology.* 2010; 54(3):248-250.
245. Marosi C, Budka H, Grimm G, et al. Fatal encephalitis in a patient with chronic graft-versus-host disease. *Bone marrow transplantation.* 1990; 6(1):53-57.
246. Martino S. Cytomegalovirus ventriculitis after haematopoietic stem cell transplantation: A rare medical emergency. *Bone marrow transplantation.* 2012; 47(S140-S141).
247. Matsukawa T, Goto H, Takahashi K, et al. A fatal case of cytomegalovirus ventriculoencephalitis in a mycosis fungoides patient who received multiple umbilical cord blood cell transplantations. *International journal of hematology.* 2012; 95(2):217-222.
248. Matsuo Y, Kamezaki K, Takeishi S, et al. Encephalomyelitis mimicking multiple sclerosis associated with chronic graft-versus-host disease after allogeneic bone marrow transplantation. *Internal medicine (Tokyo, Japan).* 2009; 48(16):1453-1456.
249. Maurice C. Cerebellar ataxia following allogenic stem cell transplant: A clinical approach to a differential diagnosis. *Neurology.* 2018; 90(15).
250. Meignin V, Gluckman E, Gambaraelli D, et al. Meningioma in long-term survivors after allogeneic bone marrow transplantation. *Bone marrow transplantation.* 1998; 22(7):723-724.
251. Meng LC, Zhang W, Wang ZX, Gao F, Yuan Y. *Clinical and muscular pathological features with chronic graft-versus-host disease-related polymyositis: One case report.* Vol 452012.
252. Meng L, Ji S, Wang Q, Bu B. Polymyositis as a manifestation of chronic graft-versus-host disease after allo-HSCT. *Clinical case reports.* 2018; 6(9):1723-1726.
253. Messina G, Quartarone E, Console G, et al. Wernicke's encephalopathy after allogeneic stem cell transplantation. *Tumori.* 2007; 93(2):207-209.
254. Meyer MA. Elevated basal ganglia glucose metabolism in cyclosporine neurotoxicity: a positron emission tomography imaging study. *Journal of neuroimaging : official journal of the American Society of Neuroimaging.* 2002; 12(1):92-93.
255. Michelis FV, Bril V, Lipton JH. A case report and literature review of chronic graft-versus-host disease manifesting as polymyositis. *International journal of hematology.* 2015; 102(1):144-146.
256. Mielke S, Potthoff K, Feuerhake F, et al. Fatal leukoencephalopathy after reduced-intensity allogeneic stem cell transplantation. *Onkologie.* 2007; 30(1-2):49-52.
257. Miura Y, Kami M, Kusumi E, et al. Exacerbation of chronic epidural abscess following a fludarabine-based preparative regimen. *American journal of hematology.* 2006; 81(3):222-224.
258. Moesen I, Kidd DP. Bilateral Inflammatory Optic Neuropathy Related to Graft versus Host Disease Following Allogeneic Bone Marrow Transplantation for Hodgkin Disease. *Neuro-ophthalmology (Aeolus Press).* 2014; 38(4):224-229.
259. Miller GG, Boivin G, Dummer JS, et al. Cytomegalovirus ventriculoencephalitis in a peripheral blood stem cell transplant recipient. *Clinical infectious diseases : an official publication of the Infectious Diseases Society of America.* 2006; 42(4):e26-29.
260. de Almeida SM, Teive HA, Brandi I, et al. Fatal Bacillus cereus meningitis without inflammatory reaction in cerebral spinal fluid after bone marrow transplantation. *Transplantation.* 2003; 76(10):1533-1534.
261. Mori T, Mihara A, Yamazaki R, et al. Myelitis associated with human herpes virus 6 (HHV-6) after allogeneic cord blood transplantation. *Scandinavian journal of infectious diseases.* 2007; 39(3):276-278.
262. Moskowitz A, Nolan C, Lis E, Castro-Malaspina H, Perales MA. Posterior reversible encephalopathy syndrome due to sirolimus. *Bone marrow transplantation.* 2007; 39(10):653-654.
263. Motohashi K, Hagihara M, Ito S, et al. Cerebral venous sinus thrombosis after allogeneic stem cell transplantation. *International journal of hematology.* 2010; 91(1):154-156.
264. Mueller-Mang C, Mang TG, Kalhs P, Thurnher MM. Imaging characteristics of toxoplasmosis encephalitis after bone marrow transplantation: report of two cases and review of the literature. *Neuroradiology.* 2006; 48(2):84-89.
265. Muftuoglu M, Olson A, Marin D, et al. Allogeneic BK virus-specific t cells for progressive multifocal leukoencephalopathy. *New England Journal of Medicine.* 2018; 379(15):1443-1451.
266. Maillard-Lefebvre H, Morell-Dubois S, Lambert M, et al. Graft-versus-host disease-related polymyositis. *Clinical rheumatology.* 2010; 29(4):431-433.
267. Nagafuji K, Eto T, Hayashi S, et al. Donor lymphocyte transfusion for the treatment of Epstein-Barr virus-associated lymphoproliferative disorder of the brain. *Bone marrow transplantation.* 1998; 21(11):1155-1158.
268. Najera JE, Alousi A, De Lima M, Ciurea SO. Akinetic mutism-a serious complication to tacrolimus-based GVHD prophylaxis. *Bone marrow transplantation.* 2013; 48(1):157-158.
269. Nanno S, Nakane T, Okamura H, et al. Disseminated Hormographiella aspergillata infection with involvement of the lung, brain, and small intestine following allogeneic hematopoietic stem cell transplantation: case report and literature review. *Transplant infectious disease : an official journal of the Transplantation Society.* 2016; 18(4):611-616.
270. Nenoff P, Kellermann S, Schober R, et al. Rhinocerebral zygomycosis following bone marrow transplantation in chronic myelogenous leukaemia. Report of a case and review of the literature. *Mycoses.* 1998; 41(9-10):365-372.
271. Noda Y, Kodama K, Yasuda T, Takahashi S. Calcineurin-inhibitor-induced pain syndrome after bone marrow transplantation. *Journal of anesthesia.* 2008; 22(1):61-63.
272. Nozzoli C, Bartolozzi B, Guidi S, et al. Epstein-Barr virus-associated post-transplant lymphoproliferative disease with central nervous system involvement after unrelated allogeneic hematopoietic stem cell transplantation. *Leukemia & lymphoma.* 2006; 47(1):167-169.
273. Neumann M, Blau IW, Burmeister T, et al. Intrathecal application of donor lymphocytes in leukemic meningeosis after allogeneic stem cell transplantation. *Annals of hematology.* 2011; 90(8):911-916.
274. Nagashima T, Sato F, Chuma T, et al. Chronic demyelinating polyneuropathy in graft-versus-host disease following allogeneic bone marrow transplantation. *Neuropathology : official journal of the Japanese Society of Neuropathology.* 2002; 22(1):1-8.
275. Neppalli AK, Johnston L. Central Nervous System Involvement of Chronic Graft Versus Host Disease. *Biology of Blood and Marrow Transplantation.* 2016; 22(3):S407.
276. Nakazato T, Nagasaki A, Nakamura K, et al. Reversible posterior leukoencephalopathy syndrome associated with tacrolimus therapy. *Internal medicine (Tokyo, Japan).* 2003; 42(7):624-625.
277. Ng CF, Wan Asyraf WZ, Khoo CS, et al. Acute disseminated encephalomyelitis in post-allogeneic peripheral blood stem cell transplantation with subclinical cytomegalovirus infection. *Multiple Sclerosis Journal.* 2018; 24(3):420-421.
278. Nakayama Y, Kamio Y, Kato N, Murayama Y. Extracranial-Intracranial Bypass for Cerebral Vasculitis After Graft-Versus-Host Disease: Case Report and Review of the Literature. *World neurosurgery.* 2019; 123:193-196.
279. O'Shaughnessy D, Goldman JM, Roddie M, Schofield JB. Dizziness and confusion after bone marrow transplantation. *Bmj.* 1994; 309(6949):262-265.
280. Ostronoff F, Ostronoff M, Fernandes HS, et al. Evidence for a graft-versus-leukemia effect in the central nervous system. *Leukemia & lymphoma.* 2008; 49(2):365-369.
281. Ostronoff F, Perales MA, Stubblefield MD, Hsu KC. Rituximab-responsive Guillain-Barre syndrome following allogeneic hematopoietic SCT. *Bone marrow transplantation.* 2008; 42(1):71-72.
282. O'Toole J, Ades S, Waters BL, Agarwal Z, Lamba G. Delayed human herpes virus 6 encephalitis in a patient with allogeneic stem cell transplant. *Leukemia & lymphoma.* 2015; 56(9):2709-2710.
283. T O. Guillain-Barre syndrome after allogeneic haematopoietic stem cell transplantation. *Bone marrow transplantation.* 2013; 48:S534-S535.
284. Onose M, Kawanishi C, Onishi H, et al. Neuroleptic malignant syndrome following BMT. *Bone marrow transplantation.* 2002; 29(9):803-804.
285. Ohashi H, Kato C, Fukami S, Saito H, Hamaguchi M. Leukemic relapse in the central nervous system after allogeneic stem cell transplantation with complete remission in the bone marrow and donor-type chimerism: report of two cases. *American journal of hematology.* 2005; 79(2):142-146.

286. Pace MT, Slovis TL, Kelly JK, Abella SD. Cyclosporin A toxicity: MRI appearance of the brain. *Pediatric radiology.* 1995; 25(3):180-183.
287. Parameswaran L, Rao A, Chastain K, et al. A Case of Adult Intestinal Toxemia Botulism During Prolonged Hospitalization in an Allogeneic Hematopoietic Cell Transplant Recipient. *Clinical Infectious Diseases.* 2017; 66:S99-S102.
288. Peman J, Jarque I, Frasquet J, et al. Unexpected postmortem diagnosis of Acanthamoeba meningoencephalitis following allogeneic peripheral blood stem cell transplantation. *American journal of transplantation : official journal of the American Society of Transplantation and the American Society of Transplant Surgeons.* 2008; 8(7):1562-1566.
289. Pelosini M, Focosi D, Rita F, et al. Progressive multifocal leukoencephalopathy: report of three cases in HIV-negative hematological patients and review of literature. *Annals of hematology.* 2008; 87(5):405-412.
290. Picardi M, De Rosa G, Di Salle F, Pezzullo L, Raiola A, Rotoli B. Post-transplant cerebral toxoplasmosis diagnosed by magnetic resonance imaging. *Haematologica.* 1998; 83(6):570-572.
291. Philippen LS, T.; Binder, A.; Both, M.; Schub, N.; Gramatzki, M.; Günther, A. Unusual GvHD of the CNS mimicking presenting with transverse myelitis and endophthalmitis. *Oncology research and treatment.* 2017; 40(63).
292. F P. Myasthenia gravis like syndrome as atypical manifestation of chronic graft versus host disease. *Haematologica.* 2009:219.
293. Pirotte M, Forte F, Lutteri L, et al. Neuronal surface antibody-mediated encephalopathy as manifestation of chronic graft-versus-host disease after allogeneic hematopoietic stem cell transplantation. *Journal of neuroimmunology.* 2018; 323:115-118.
294. Pavlović M, Rajić L, Barišić N, et al. P17.12 Wernicke's encephalopathy in an adolescent after allogeneic stem cell transplantation: a case report. *European Journal of Paediatric Neurology.* 2011; 15:S102.
295. Petrova-Drus K. Respiratory failure and diffuse toxoplasmosis in the post allogeneic stem cell transplant setting-a difficult diagnosis. *Laboratory Investigation.* 2015; 9A-10A.
296. Polchlopek Blasiak K, Simonetta F, Vargas MI, Chalandon Y. Central nervous system graft-versus-host disease (CNS-GvHD) after allogeneic haematopoietic stem cell transplantation. *BMJ case reports.* 2018; 2018.
297. Powell A, Sy J, Barnett Y, et al. Post-transplant lymphoproliferative disorder in skeletal muscle arising seven years after allogeneic stem cell transplant. *Journal of Neurology, Neurosurgery and Psychiatry.* 2018; 89(6):e15-e16.
298. Caihong Q, Weimin L, Jieming Z. Elevation of blood ciclosporin levels by voriconazole leading to leukoencephalopathy. *Journal of pharmacology & pharmacotherapeutics.* 2013; 4(4):294-297.
299. Rabinstein AA, Dispenzieri A, Micallef IN, Inwards DJ, Litzow MR, Wijdicks EF. Acute neuropathies after peripheral blood stem cell and bone marrow transplantation. *Muscle & nerve.* 2003; 28(6):733-736.
300. Rodriguez V, Kuehnle I, Heslop HE, Khan S, Krance RA. Guillain-Barre syndrome after allogeneic hematopoietic stem cell transplantation. *Bone marrow transplantation.* 2002; 29(6):515-517.
301. Raheja A, Sowder A, Palmer C, Rodriguez FJ, Couldwell WT. Epstein-Barr virus-associated smooth muscle tumor of the cavernous sinus: a delayed complication of allogenic peripheral blood stem cell transplantation: case report. *Journal of neurosurgery.* 2017; 126(5):1479-1483.
302. Rasool N, Boudreault K, Lessell S, Prasad S, Cestari DM. Tacrolimus Optic Neuropathy. *Journal of neuro-ophthalmology : the official journal of the North American Neuro-Ophthalmology Society.* 2018; 38(2):160-166.
303. Raza S, Ullah K, Ahmed P, Satti TM, Kamal MK, Chaudhry QU. Cyclosporine induced neurotoxicity in a stem cell transplant recipient. *JPMA. The Journal of the Pakistan Medical Association.* 2007; 57(12):611-613.
304. Reddy SM, Winston DJ, Territo MC, Schiller GJ. CMV central nervous system disease in stem-cell transplant recipients: an increasing complication of drug-resistant CMV infection and protracted immunodeficiency. *Bone marrow transplantation.* 2010; 45(6):979-984.
305. Abstracts of the Nineteenth Meeting of the European Neurological Society. June 20-24, 2009. Milan, Italy. *Journal of neurology.* 2009; 256 Suppl 2:S3-250.
306. Richard S, Fruchtman S, Scigliano E, Skerrett D, Najfeld V, Isola L. An immunological syndrome featuring transverse myelitis, Evans syndrome and pulmonary infiltrates after unrelated bone marrow transplant in a patient with severe aplastic anemia. *Bone marrow transplantation.* 2000; 26(11):1225-1228.
307. Resnick IB, Gomori JM, Kiselgoff D, et al. Spinal epidural lipomatosis following haploidentical allogeneic bone marrow transplantation for non-Hodgkin lymphoma. *Clinical transplantation.* 2004; 18(6):762-765.
308. Rieux C, Gautheret-Dejean A, Challine-Lehmann D, Kirch C, Agut H, Vernant JP. Human herpesvirus-6 meningoencephalitis in a recipient of an unrelated allogeneic bone marrow transplantation. *Transplantation.* 1998; 65(10):1408-1411.
309. Roy V, Ali LI, Carter TH, Selby GB. Successful non-surgical treatment of disseminated polymicrobial fungal infection in a patient with pancytopenia and graft-versus-host disease. *The Journal of infection.* 2000; 41(3):273-275.
310. Reddy P, Davenport R, Ratanatharathorn V, et al. West Nile virus encephalitis causing fatal CNS toxicity after hematopoietic stem cell transplantation. *Bone marrow transplantation.* 2004; 33(1):109-112.
311. Robuccio A, Ssentongo P, Sather MD, Claxton DF, Gilliam FG. Intractable myoclonic seizures in an allogeneic stem cell transplant recipient: A rare case of myoclonic epilepsy. *Epilepsy & behavior case reports.* 2015; 4:48-51.
312. Ramlal R, Ravulapati S, Ely E, et al. Multifocal Necrotizing Leukoencephalopathy – a Previously Undescribed Entity Following Allogeneic Stem Cell Transplantation. *Biology of Blood and Marrow Transplantation.* 2016; 22(3):S349.
313. Renner C, Hegenbarth U, Schneider JP, Meixensberger J. Fatal bihemispheric intracerebral hemorrhage after burrhole evacuation of chronic subdural hematoma in a bone marrow-transplanted patient: case report and review of the literature. *Surgical neurology.* 2004; 62(3):260-263; discussion 263.
314. Reynolds J. Rare case of HHV6 encephalitis in post allogeneic transplant recipient. *Biology of Blood and Marrow Transplantation.* 2017; 23(3):S107.
315. M. R. Hemorrhage in posterior reversible encephalopathy syndrome as a complication of tacrolimus therapy. *American Journal of Respiratory and Critical Care Medicine.* 2017; 195.
316. Saad AG, Alyea EP, 3rd, Wen PY, Degirolami U, Kesari S. Graft-versus-host disease of the CNS after allogeneic bone marrow transplantation. *Journal of clinical oncology : official journal of the American Society of Clinical Oncology.* 2009; 27(30):e147-149.
317. Sakamoto K, Imamura T, Niwa F, et al. Dermatomyositis developed in a recipient of allogeneic BMT; the differentiation of chronic GVHD and autoimmune disease. *Bone marrow transplantation.* 2012; 47(4):603-604.
318. Sakai M, Ohashi K, Ohta K, et al. Immune-mediated myelopathy following allogeneic stem cell transplantation. *International journal of hematology.* 2006; 84(3):272-275.
319. Sarva H, Graber J, Remanan R, Rosenblum M, Omuro A. CMV encephalitis in BMT recipients. *Bone marrow transplantation.* 2012; 47(2):318-320.
320. Sawant RB, Rajadhyaksha SB. Plasma exchange for thrombotic thrombocytopenic purpura following hematopoietic stem cell transplantation. *The Journal of the Association of Physicians of India.* 2005; 53:981-983.
321. Sekiguchi K, Kanzawa M, Nishino I, Kanda F, Toda T, Kohara N. WS2-1. A case of slowly progressive muscular atrophy in post hematopoietic stem cell transplantation: Electrophysiological and pathological findings in musculoskeletal chronic graft-versus-host disease (cGVHD). *Clinical Neurophysiology.* 2013; 124(8):e29.
322. Seok JH, Ahn K, Park HJ. Diffusion MRI findings of cytomegalovirus-associated ventriculitis: a case report. *The British journal of radiology.* 2011; 84(1005):e179-181.
323. Shargian-Alon L, Raanani P, Rozovski U, Siegal T, Yust-Katz S, Yeshurun M. Immune mediated cerebellar ataxia: An unknown manifestation of graft-versus-host disease. *Acta haematologica.* 2019; 141(1):19-22.
324. Shbarou RM, Chao NJ, Morgenlander JC. Cyclosporin A-related cerebral vasculopathy. *Bone marrow transplantation.* 2000; 26(7):801-804.
325. Sheikh SI, Stemmer-Rachamimov A, Attar EC. Autopsy diagnosis of progressive multifocal leukoencephalopathy with JC virus-negative CSF after cord blood stem-cell transplantation. *Journal of clinical oncology : official journal of the American Society of Clinical Oncology.* 2009; 27(23):e46-47.
326. Shimizu H, Saitoh T, Koya H, et al. Discrepancy in EBV-DNA load between peripheral blood and cerebrospinal fluid in a patient with isolated CNS post-transplant lymphoproliferative disorder. *International journal of hematology.* 2011; 94(5):495-498.

327. Shigemura T, Nishina S, Nakazawa H, Matsuda K, Yaguchi T, Nakazawa Y. Early detection of Rhizopus DNA in the serum of a patient with rhino-orbital-cerebral mucormycosis following allogeneic hematopoietic stem cell transplantation. *International journal of hematology.* 2016; 103(3):354-355.

328. Short JH, Sen A. 62-Year-Old Man With Encephalopathy and Fatigue After Allogeneic Bone Marrow Transplant. *Mayo Clinic proceedings.* 2018; 93(8):1139-1143.

329. Shortt J, Hutton E, Faragher M, Spencer A. Central nervous system graft-versus-host disease post allogeneic stem cell transplant. *British journal of haematology.* 2006; 132(2):245-247.

330. Solh M, Balls J, Smith T, Khaled Y. Guilain-Barre' Syndrome (GBS) Post Adult Cord Blood Transplantation in a Patient with Chronic Lymphocytic Leukemia (CLL). *Biology of Blood and Marrow Transplantation*. 2014; 20(2):S281.

331. Solmaz S, Gereklioglu C, Tan M, et al. A Rare Complication Developing After Hematopoietic Stem Cell Transplantation: Wernicke's Encephalopathy. *Turkish journal of haematology : official journal of Turkish Society of Haematology.* 2015; 32(4):367-370.

332. Sostak P, Padovan CS, Eigenbrod S, et al. Cerebral angiitis in four patients with chronic GVHD. *Bone marrow transplantation.* 2010; 45(7):1181-1188.

333. Steg RE, Kessinger A, Wszolek ZK. Cortical blindness and seizures in a patient receiving FK506 after bone marrow transplantation. *Bone marrow transplantation.* 1999; 23(9):959-962.

334. Shintaku M, Kaneda D, Tada K, Katano H, Sata T. Human herpes virus 6 encephalomyelitis after bone marrow transplantation: report of an autopsy case. *Neuropathology : official journal of the Japanese Society of Neuropathology.* 2010; 30(1):50-55.

335. Shimizu R, Ohwada C, Nagao Y, et al. The Successful Treatment of a Cord Blood Transplant Recipient with Varicella Zoster Virus Meningitis, Radiculitis and Myelitis with Foscarnet. *Internal medicine (Tokyo, Japan).* 2017; 56(3):353-356.

336. Schenk T. Hemophagocytic syndrome after matched unrelated allogeneic stem cell transplantation. *Onkologie.* 2011; 34.

337. Solaro C, Murialdo A, Giunti D, Mancardi G, Uccelli A. Central and peripheral nervous system complications following allogeneic bone marrow transplantation. *European journal of neurology.* 2001; 8(1):77-80.

338. Stojanoski Z. Posterior reversible encephalopathy syndrome. *Bone marrow transplantation.* 2011; 46(S331).

339. Steurer M, Clausen J, Gotwald T, et al. Progressive multifocal leukoencephalopathy after allogeneic stem cell transplantation and posttransplantation rituximab. *Transplantation.* 2003; 76(2):435-436.

340. Sassi M. PRES syndrome induced by cyclosporin with normal blood concentration. *Drug Safety.* 2014; 37(10):877.

341. Sinaei F, Khodabakhsh A, Alimoghaddam K, Nafissi S. Delayed-onset inflammatory polyneuropathy without graft versus host disease after bone marrow transplantation. *Muscle & nerve.* 2018; 58(2):E11-E13.

342. Seong DC, Przepiorka D, Bruner JM, Van Tassel P, Lo WK, Champlin RE. Leptomeningeal toxoplasmosis after allogeneic marrow transplantation. Case report and review of the literature. *American journal of clinical oncology.* 1993; 16(2):105-108.

343. Schrader C, Stingele R, Brück W, et al. 9229 POSTER Severe Central Nervous System (CNS) Graft Versus Host Disease (GVHD) in a Patient Without Any Other GvHD Symptoms After Allogeneic Stem Cell Transplantation. *European Journal of Cancer.* 2011; 47:S647-S648.

344. Steinberg A, Gorman E, Tannenbaum J. Thiamine deficiency in stem cell transplant patients: a case series with an accompanying review of the literature. *Clinical lymphoma, myeloma & leukemia.* 2014; 14 Suppl:S111-113.

345. Sweany JM, Bartynski WS, Boardman JF. "Recurrent" posterior reversible encephalopathy syndrome: report of 3 cases--PRES can strike twice! *Journal of computer assisted tomography.* 2007; 31(1):148-156.

346. Saikawa Y, Horisawa T, Mase S, et al. Right homonymous hemianopsia following allogenic haematopoietic stem cell transplantation. *European journal of haematology.* 2008; 80(6):551.

347. Sanchez-Quintana A, Brena-Atienza J, Marrero-Santos C, Alvarez-Acosta L. Late relapse of progressive multifocal leucoencephalopathy postallogenic transplant in a young patient with CLL. *BMJ case reports.* 2013; 2013.

348. Suzuki S, Mori T, Mihara A, et al. Immune-mediated motor polyneuropathy after hematopoietic stem cell transplantation. *Bone marrow transplantation.* 2007; 40(3):289-291.

349. Sevindik OG, Alacacioglu I, Katgi A, et al. Renal and neurological response with eculizumab in a patient with transplant associated thrombotic microangiopathy after allogeneic hematopoietic progenitor cell transplantation. *Case reports in hematology.* 2015; 2015:425410.

350. Sakai M, Ohashi K, Kobayashi T, et al. Meningeal hematopoiesis following radiation myelitis in a hematopoietic stem-cell transplant recipient. *American journal of hematology*. 2005; 79(4):291-293.

351. Schmidt-Hieber M, Okuducu AF, Stoltenburg G, et al. Hemosiderin deposits in chronic graft-vs.-host disease related myopathy. *European journal of haematology.* 2005; 75(6):522-526.

352. Suzuki J, Ashizawa M, Okuda S, et al. Varicella zoster virus meningoencephalitis after allogeneic hematopoietic stem cell transplantation. *Transplant infectious disease : an official journal of the Transplantation Society.* 2012; 14(4):E7-12.

353. Targhetta C. High dose cyclophosphamide for the treatment of severe miller-fisher syndrome, following allogeneic stem cell transplantation. *Haematologica.* 2013; 98(226).

354. Tamaki H, Kawakami M, Ikegame K, et al. Successful treatment of tacrolimus (FK506)-related leukoencephalopathy with cerebral hemorrhage in a patient who underwent nonmyeloablative stem cell transplantation. *International journal of hematology.* 2004; 80(3):291-294.

355. Andy Tang SO, Leong TS, Ruixin T, Chua HH, Chew LP. Thrombotic thrombocytopenic purpura-like syndrome associated with arcanobacterium pyogenes endocarditis in a post-transplant patient: A case report. *The Medical journal of Malaysia.* 2018; 73(5):344-346.

356. Takeda C, Mashima Y, Suzuki S, Okamoto S. *A case of acute lymphocytic leukemia with bilateral optic symptoms to those of ischemic optic neuropathy after bone marrow transplantation.* Vol 491998.

357. Tauro S, Toh V, Osman H, Mahendra P. Varicella zoster meningoencephalitis following treatment for dermatomal zoster in an alloBMT patient. *Bone marrow transplantation.* 2000; 26(7):795-796.

358. Takahashi S, Horiguchi T, Mikami S, Kitamura Y, Kawase T. Subcortical intracerebral hemorrhage caused by mucormycosis in a patient with a history of bone-marrow transplantation. *Journal of stroke and cerebrovascular diseases : the official journal of National Stroke Association.* 2009; 18(5):405-406.

359. Tanaka M, Taguchi J, Hyo R, et al. Human herpesvirus-6 encephalitis after unrelated cord blood transplantation. *Leukemia & lymphoma.* 2005; 46(4):561-566.

360. Takahashi M. HHV6 assosiated limbic encephalitis with sever skin rash after cord blood transplantation: Report of an autopsy case. *Neuropathology : official journal of the Japanese Society of Neuropathology.* 2013; 33(3):388.

361. Tanase A, Varady Z, Coriu D, et al. Rhino-cerebral zygomycosis after allogeneic transplant: case report and literature review. *Romanian journal of morphology and embryology = Revue roumaine de morphologie et embryologie.* 2011; 52(2):715-718.

362. Tomaszewska A, Nasilowska-Adamska B, Dzieciatkowski T, Marianska B. Simultaneous human herpesvirus 6-associated encephalitis and Guillain-Barre syndrome in a patient after matched unrelated donor haematopoietic stem cell transplantation. *Archives of medical science : AMS.* 2010; 6(2):288-290.

363. Thien SY, Chung SJ, Tan AL, Hwang WY, Tan BH, Tan TT. Recurrent trichosporonosis with central nervous system involvement in an allogeneic hematopoietic stem cell transplant recipient. *Transplant infectious disease : an official journal of the Transplantation Society.* 2016; 18(5):768-772.

364. Terada M, Nakamagoe K, Obara N, et al. Chronic Graft-versus-host Disease Presenting with Multiple Punctate Intracranial Lesions on Contrast-enhanced Magnetic Resonance Imaging. *Internal medicine (Tokyo, Japan).* 2017; 56(3):363-368.

365. Tseng WE, Wu T, Cheng MY, et al. Preemptive therapy with ganciclovir and cytomegalovirus hyperimmune globulin delayed the onset of Epstein-Barr virus-associated post-transplant acute limbic encephalitis. *J Neurol Sci.* 2015; 359(1-2):124-126.
366. Teksam M, Casey SO, Michel E, Truwit CL. Subarachnoid hemorrhage associated with cyclosporine A neurotoxicity in a bone-marrow transplant recipient. *Neuroradiology.* 2001; 43(3):242-245.
367. Thomas A, Stein CK, Gentile TC, Shah CM. Isolated CNS relapse of CML after bone marrow transplantation. *Leuk Res.* 2010; 34(4):e113-114.
368. Terrettaz M, Verholen F, Passweg J, Knipp S, Burkhard PR, Chalandon Y. Steroid-responsive cauda equina syndrome associated with GVHD after allogeneic hematopoietic stem cell transplantation. *Bone marrow transplantation.* 2008; 41(3):315-316.
369. Takahashi K, Kashihara K, Shinagawa K, Yoshino T, Abe K, Harada M. Myositis as a manifestation of chronic graft-versus-host disease. *Internal medicine (Tokyo, Japan).* 2000; 39(6):482-485.
370. Takahata M, Hashino S, Izumiyama K, Chiba K, Suzuki S, Asaka M. Cyclosporin A-induced encephalopathy after allogeneic bone marrow transplantation with prevention of graft-versus-host disease by tacrolimus. *Bone marrow transplantation.* 2001; 28(7):713-715.
371. Takatsuka H, Okamoto T, Yamada S, et al. New imaging findings in a patient with central nervous system dysfunction after bone marrow transplantation. *Acta haematologica.* 2000; 103(4):203-205.
372. Tamaki M, Nozaki K, Onishi M, Yamamoto K, Ujiie H, Sugahara H. Fungal meningitis caused by Lomentospora prolificans after allogeneic hematopoietic stem cell transplantation. *Transplant infectious disease : an official journal of the Transplantation Society.* 2016; 18(4):601-605.
373. Tateno T, Onozawa M, Hashiguchi J, et al. Disseminated toxoplasmosis after hematopoietic stem cell transplantation showing unusual magnetic resonance images. *Transplant infectious disease : an official journal of the Transplantation Society.* 2017; 19(4).
374. Terasawa T, Ohashi H, Tsushita K, et al. Failure to detect Epstein-Barr virus (EBV) DNA in plasma by real-time PCR in a case of EBV-associated posttransplantation lymphoproliferative disorder confined to the central nervous system. *International journal of hematology.* 2002; 75(4):416-420.
375. Thomas C, Mileusnic D, Carey RB, Kampert M, Anderson D. Fatal Chaetomium cerebritis in a bone marrow transplant patient. *Human pathology.* 1999; 30(7):874-879.
376. Thone J, Lamprecht S, Hohaus A, Erbguth F, Bickel A. Guillain-Barre syndrome as leading manifestation of graft-versus-host disease in an allogeneic bone marrow transplanted patient. *J Neurol Sci.* 2010; 292(1-2):114-116.
377. To LB, Chin DK, Blumbergs PC, Burrow DD, Juttner CA. Central nervous system relapse after bone marrow transplantation for acute myeloid leukemia. *Cancer.* 1983; 52(12):2236-2239.
378. Tomonari A, Tojo A, Adachi D, et al. Acute disseminated encephalomyelitis (ADEM) after allogeneic bone marrow transplantation for acute myeloid leukemia. *Annals of hematology.* 2003; 82(1):37-40.
379. Iemura T, Kitano T, Ishii A, et al. Metronidazole-induced encephalopathy during treatment for refractory diarrhea after cord blood transplantation. *Clin J Gastroenterol.* 2019.
380. Torelli GF, Natalino F, Barberi W, et al. Early onset of posterior reversible encephalopathy syndrome (PRES) during Cyclosporine-A infusion. *Leuk Res.* 2011; 35(10):1423-1424.
381. Toumeh A, Phinney R, Kobalka P, Mohamed I. Bilateral myeloid sarcoma of the breast and cerebrospinal fluid as a relapse of acute myeloid leukemia after stem-cell transplantation: a case report. *Journal of clinical oncology : official journal of the American Society of Clinical Oncology.* 2012; 30(22):e199-201.
382. Tummala S, Rezvani K. Treating progressive multifocal leukoencephalopathy with expanded third party BK virus specific cytoxic T cells in acute myeloid leukemia patient following cord blood transplantation. *Neurology.* 2018; 90(15).
383. Urbano-Marquez A, Estruch R, Grau JM, et al. Inflammatory myopathy associated with chronic graft-versus-host disease. *Neurology.* 1986; 36(8):1091-1093.
384. Uoshima N, Karasuno T, Yagi T, et al. Late onset cyclosporine-induced cerebral blindness with abnormal SPECT imagings in a patient undergoing unrelated bone marrow transplantation. *Bone marrow transplantation.* 2000; 26(1):105-108.
385. Vande Broek I, Schots R. Fatal cerebral zygomycosis breakthrough in a patient with acute lymphoblastic leukemia on voriconazole prophylaxis after cord blood SCT. *Bone marrow transplantation.* 2009; 44(11):765-766.
386. Vasko R, Groenewold F, Korsten P, Muller GA, Koziolek M. Plasmapheresis-refractory thrombotic microangiopathy in a hematopoietic stem cell transplant recipient. *Therapeutic apheresis and dialysis : official peer-reviewed journal of the International Society for Apheresis, the Japanese Society for Apheresis, the Japanese Society for Dialysis Therapy.* 2011; 15(5):507-509.
387. Vesole AS, Nagahama Y, Granner MA, Howard MA, Kawasaki H, Dlouhy BJ. Drug-resistant epilepsy development following stem cell transplant and cyclosporine neurotoxicity induced seizures: Case report in an adult and analysis of reported cases in the literature. *Epilepsy and Behavior Case Reports.* 2018; 10:8-13.
388. Vilter RW. Vidarabine-associated encephalopathy and myoclonus. *Antimicrob Agents Chemother.* 1986; 29(5):933-935.
389. Visser AM, van Doornum GJ, Cornelissen JJ, van den Bent MJ. Severe amnesia due to HHV-6 encephalitis after allogenic stem cell transplantation. *European neurology.* 2005; 54(4):233-234.
390. Vogl U, Leitner G, Dal-Bianco A, et al. Complete neurologic and cognitive recovery after plasmapheresis in a patient with chronic inflammatory demyelinating polyneuropathy after allogeneic hematopoietic stem cell transplantation. *Wiener klinische Wochenschrift.* 2016; 128(9-10):384-386.
391. Voss M, Bischof F. Recurrent myelitis after allogeneic stem cell transplantation. Report of two cases. *BMC neurology.* 2010; 10:76.
392. Warwick A, Ferrieri P, Burke B, Blazar BR. Presumptive invasive Chrysosporium infection in a bone marrow transplant recipient. *Bone marrow transplantation.* 1991; 8(4):319-322.
393. Wei X, Zhao M, Li Q, Xiao X, Zhu L. Tacrolimus-Induced Pain Syndrome After Bone Marrow Transplantation: A Case Report and Literature Review. *Transplantation proceedings.* 2018; 50(10):4090-4095.
394. Wylie KM, Blanco-Guzman M, Wylie TN, et al. High-throughput sequencing of cerebrospinal fluid for diagnosis of chronic Propionibacterium acnes meningitis in an allogeneic stem cell transplant recipient. *Transplant infectious disease : an official journal of the Transplantation Society.* 2016; 18(2):227-233.
395. Wadhwani NR, Armstrong AE, Schneiderman J, Smith C, Ryan M, Kurup S. Chronic Graft Vs. Host Disease of the CNS - a Rare Autopsied Case. *Biology of Blood and Marrow Transplantation.* 2015; 21(2):S326.
396. Ward KN, White RP, Mackinnon S, Hanna M. Human herpesvirus-7 infection of the CNS with acute myelitis in an adult bone marrow recipient. *Bone marrow transplantation.* 2002; 30(12):983-985.
397. Wroblewska M, Gil LA, Komarnicki MA. Successful treatment of Epstein-Barr virus-related post-transplant lymphoproliferative disease with central nervous system involvement following allogeneic haematopoietic stem cell transplantation - a case study. *Central-European journal of immunology.* 2015; 40(1):122-125.
398. Walter SH, Bertz H, Gerling J. Bilateral optic neuropathy after bone marrow transplantation and cyclosporin A therapy. *Graefe's archive for clinical and experimental ophthalmology = Albrecht von Graefes Archiv fur klinische und experimentelle Ophthalmologie.* 2000; 238(6):472-476.
399. Yacoub AB, L.; Pasikhova, Y.; Kharfan-Dabaja, M. A.; Sandin, R. L.; Greene, J. Cytomegalovirus encephalitis in an allogeneic hematopoietic cell transplant recipient: A case report and review of literature. *Infectious Diseases in Clinical Practice.* 2014; 22(4):e95-e99.
400. Yadav SP, Kohli S, Nivargi S, Thakkar D, Rastogi N. Successful management of progressive multifocal leukoencephalopathy post TCR alphabeta/CD19 depleted haploidentical stem cell transplant. *Blood.* 2018; 132.

401. Yoshida H, Ohshima K, Toda J, et al. Significant improvement following combination treatment with mefloquine and mirtazapine in a patient with progressive multifocal leukoencephalopathy after allogeneic peripheral blood stem cell transplantation. *International journal of hematology.* 2014; 99(1):95-99.
402. Yu J, Sun L, Lin W. Reversible posterior leukoencephalopathy associated with chronic graft-versus-host disease: A case report. *Exp Ther Med.* 2016; 11(6):2509-2512.
403. Yoo JS, Lindsay; Wang, PhD, Zi-Xuan; and Kenyon, Lawrence C. Graft Versus Host Disease of the Brain Following Allogeneic Stem Cell Transplant for Myelodysplastic Syndrome. *Department of Pathology, Anatomy, and Cell Biology Faculty Papers.* 2012; 91.
404. Yamashita Y, Kusakabe S, Toda J, et al. Sequential Onset of Varicella-Zoster Virus Encephalomeningitis and Progressive Multifocal Leukoencephalopathy in an Allogeneic Hematopoietic Stem Cell Transplant Recipient. *Experimental and clinical transplantation : official journal of the Middle East Society for Organ Transplantation.* 2018; 16(5):628-630.
405. Yoshihara S, Kato R, Inoue T, et al. Successful treatment of life-threatening human herpesvirus-6 encephalitis with donor lymphocyte infusion in a patient who had undergone human leukocyte antigen-haploidentical nonmyeloablative stem cell transplantation. *Transplantation.* 2004; 77(6):835-838.
406. Young NP, Dyck PJ, Wijdicks EF. Locked-in syndrome due to invasive fungal rhinosinusitis in an immunosuppressed patient. *The neurologist.* 2007; 13(3):158-160.
407. Yafour N, Krim A, Bouhass R, Bekadja MA. Cyclosporine-related brainstem atypical posterior reversible leukoencephalopathy syndrome following hematopoietic stem cell transplant. *Hematology/oncology and stem cell therapy.* 2016; 9(1):36-38.
408. Yamamoto H, Uchida N, Ishiwata K, et al. Possible graft-versus-host disease involving the central nervous system soon after cord blood transplantation. *American journal of hematology.* 2009; 84(11):764-766.
409. Yeral M. Guillain-barré syndrome associated with parvovirus infection after allogeneic hematopoietic stem cell transplantation. *Bone marrow transplantation.* 2014; 49.
410. Yoo C. Disseminated toxoplasmosis status post bone marrow transplantation. *Journal of neuropathology and experimental neurology.* 2013; 72(6).
411. Yoon BN, Ha CK, Lee KW, Park SH, Sung JJ. A confusing case of multiple sclerosis and central nervous system graft versus host disease. *The Korean journal of internal medicine.* 2016; 31(5):995-998.
412. Yamashita Y, Kusakabe S, Toda J, et al. Sequential Onset of Varicella-Zoster Virus Encephalomeningitis and Progressive Multifocal Leukoencephalopathy in an Allogeneic Hematopoietic Stem Cell Transplant Recipient. *Experimental and clinical transplantation : official journal of the Middle East Society for Organ Transplantation.* 2018; 16(5):628-630.
413. Yildirim M, Sayin S, Ozgur G, Ugur B, Ayli M. Case reports of central nerve system aspergillosis with rare skin lesions and rare treatment modality (intrathecal/ intralesional liposomal amphotericin-b treatment). *Leukemia Research.* 2017; 61:S17.
414. Yoshida H, Matsunaga K, Ueda T, et al. Human herpesvirus 6 meningoencephalitis successfully treated with ganciclovir in a patient who underwent allogeneic bone marrow transplantation from an HLA-identical sibling. *International journal of hematology*. 2002; 75(4):421-425.
415. Yuan C, Deberardinis C, Patel R, et al. Progressive multifocal leukoencephalopathy after allogeneic stem cell transplantation: Case report and review of the literature. *Transplant Infectious Disease.* 2018; 20(3).
416. Yoshida T, Ueki Y, Suzuki T, et al. Guillain-Barre syndrome after allogeneic bone marrow transplantation: Case report and literature review. *eNeurologicalSci.* 2016; 4:52-55.
417. Zaucha-Prazmo A, Samardakiewicz M, Dubelt J, Kowalczyk JR. Cerebral toxoplasmosis after haematopoietic stem cell transplantation. *Annals of agricultural and environmental medicine : AAEM.* 2017; 24(2):237-239.
418. Zhao J, Zu Y, Han L, et al. Treatment of Epstein-Barr virus associated central nervous system diseases after allogeneic hematopoietic stem cell transplantation with intrathecal donor lymphocyte infusion. *Bone marrow transplantation*. 2018.
419. Zhu X-Y, Kuo S-H, Wan L-P, Liu Y, Wu Y-C. *Isolated peripheral neuropathy as an unusual presentation for an extramedullary relapse of acute leukemia.* Vol 20142014.
420. Zheng X, Huang Y, Wang Z, Yan H, Pan S, Wang H. Presumed cytomegalovirus-associated retrobulbar optic neuritis in a patient after allogeneic stem cell transplantation. *Transplant infectious disease : an official journal of the Transplantation Society.* 2012; 14(2):177-179.
421. Zucchetti E. Intrathecal rituximab administration in isolated EBV-related CNS PTLD post haploidentical transplant. *Bone marrow transplantation.* 2013; 48(S530).
422. Zeiser R, Grullich C, Bertz H, et al. Late cytomegalovirus polyradiculopathy following haploidentical CD34+-selected hematopoietic stem cell transplantation. *Bone marrow transplantation.* 2004; 33(2):243-245.
423. Zangrandi A, Gasparini F, Imovilli A, et al. Atypical non-progressive semantic impairment following allogeneic bone marrow transplantation in a patient with Waldenstrom's macroglobulinemia: a case report. *The Clinical neuropsychologist.* 2018:1-13.
424. Zaja F, Barillari G, Russo D, Silvestri F, Fanin R, Baccarani M. Myasthenia gravis after allogeneic bone marrow transplantation. A case report and a review of the literature. *Acta neurologica Scandinavica.* 1997; 96(4):256-259.
425. Zaja F, Russo D, Fuga G, Perella G, Baccarani M. Rituximab for myasthenia gravis developing after bone marrow transplant. *Neurology*. 2000; 55(7):1062-1063.

Suppl. Table 4. Prospective cohorts, randomised control trial (RCT) and case-control studies characteristics.

First author	Year	N	Type	Subtype	n	Onset day	Neuro-deaths	Follow-up	ROB (NOS)
Prospective cohorts									
Ishiyama[1]	2010	21	Drug-related	Foscarnet sodium	1	early	0	1	high risk 5/9
Ishiyama[2]	2011	20	Drug-related	Foscarnet sodium	4	early	na	1	fair 6/9
Christopoulos[3]	2012	30	Drug-related	fludarabine, carmustine and thiotepa	8	na	2	32	high risk 5/9
Kroger[4]	2013	33	Drug-related	lenalidomide	4	na	na	19	high risk 5/9
DeLima[5]	2004	96	Drug-related	cytarabine (4) , tacrolimus (1)	19	na	na	12	high risk 5/9
Beglinger[6]	2011	22	Higher Cortical Functions		8	early	na	1	high risk 5/9
Scherwath *[7]	2013	239	Higher Cortical Functions		5 to 45	late	na	12	high risk 5/9
Koeppen[8]	2014	429	Immune-mediated	peripheral GVHD	20	late	na	3	fair 6/9
Van der Wagen [9]	2016	29	Drug-related	rituximab-nilotinib	2	na	na	45	high risk 5/9
Goto[10]	2018	51	Other	CNS	1	na	1	20	high risk 5/9
Greco[11]	2016	213	Infectious	viral(5)	5	early	na	3	fair 6/9

Yamane[12]	2007	46	Infectious	viral (3), bacterial (1)	4	early	1	2	fair 6/9
vanVeen[13]	2016	2623	Infectious	bacterial(11)	11	late	na	na	high risk 5/9
Martino[14]	2000	106	Infectious	protozoan (5)	5	early	2	6	fair 6/9
Ogata[15]	2013	230	Infectious, cerebrovascular, metabolic, drug-related, relapse, other	Viral (7); opioid or major tranquilizer (3)	33	early	na	2	fair 6/9
DeFilipp[16]	2018	17	Drug-related	Brentuximab Vedotin	5	late	na	6	high risk 5/9
Sostak[17]	2003	121	Infectious, cerebrovascular, metabolic, peripheral	fungal (2), viral (4), protozoan (1), bacterial (1)	46	na	6	16	high risk 5/9
Case-controls									
Conrad[18]	2016	588	Infectious	protozoan(10)	10	na	6	na	low risk (8/9)
Daly[19]	2002	603	TA-TMA		10	na	na	na	fair (6/9)
RCT									
Irle[20]	1985	56	Drug-related	methotrexate, cyclosporine	16	na	2	na	high risk

TA-TMA: transplant-associated thrombotic microangiopathy; GVHD: graft-versus-host disease; CNS: central nervous system; ROB: Risk Of Bias; NOS: Newcastle-Ottawa Scale; na: not available; * Indicates studies excluded from meta-analysis; Follow-up is presented as median in months; n: adult patients with neurologic manifestation

1. Ishiyama K, Katagiri T, Hoshino T, Yoshida T, Yamaguchi M, Nakao S. Preemptive therapy of human herpesvirus-6 encephalitis with foscarnet sodium for high-risk patients after hematopoietic SCT. *Bone marrow transplantation.* 2011; 46(6):863-869.
2. Ishiyama K, Katagiri T, Ohata K, et al. Safety of pre-engraftment prophylactic foscarnet administration after allogeneic stem cell transplantation. *Transplant infectious disease : an official journal of the Transplantation Society.* 2012; 14(1):33-39.
3. Christopoulos P, Bertz H, Ihorst G, Marks R, Wasch R, Finke J. Radiation-free allogeneic conditioning with fludarabine, carmustine, and thiotepa for acute lymphoblastic leukemia and other hematologic malignancies necessitating enhanced central nervous system activity. *Biology of blood and marrow transplantation : journal of the American Society for Blood and Marrow Transplantation.* 2012; 18(9):1430-1437.
4. Kroger N, Zabelina T, Klyuchnikov E, et al. Toxicity-reduced, myeloablative allograft followed by lenalidomide maintenance as salvage therapy for refractory/relapsed myeloma patients. *Bone marrow transplantation.* 2013; 48(3):403-407.
5. de Lima M, Couriel D, Thall PF, et al. Once-daily intravenous busulfan and fludarabine: clinical and pharmacokinetic results of a myeloablative, reduced-toxicity conditioning regimen for allogeneic stem cell transplantation in AML and MDS. *Blood.* 2004; 104(3):857-864.
6. Beglinger LJ, Mills JA, Vik SM, et al. The neuropsychological course of acute delirium in adult hematopoietic stem cell transplantation patients. *Archives of clinical neuropsychology : the official journal of the National Academy of Neuropsychologists.* 2011; 26(2):98-109.
7. Scherwath A, Schirmer L, Kruse M, et al. Cognitive functioning in allogeneic hematopoietic stem cell transplantation recipients and its medical correlates: a prospective multicenter study. *Psycho-oncology.* 2013; 22(7):1509-1516.
8. Koeppen S, Thirugnanasambanthan A, Koldehoff M. Neuromuscular complications after hematopoietic stem cell transplantation. *Supportive care in cancer : official journal of the Multinational Association of Supportive Care in Cancer.* 2014; 22(9):2337-2341.
9. van der Wagen LE, Boome Lt, Nijhof I, Schoordijk M, Meijer E, Kuball J. Effective Treatment of Severe Chronic Graft Versus Host Disease with a Combination of B-Cell Depletion and Tyrosine Kinase Inhibition. *Blood.* 2016; 128(22):4565-4565.
10. Goto T, Tanaka T, Sawa M, et al. Prospective observational study on the first 51 cases of peripheral blood stem cell transplantation from unrelated donors in Japan. *International journal of hematology.* 2018; 107(2):211-221.
11. Greco R, Noviello M, Crucitti L, et al. HHV6 Specific T-Cells Are Predictive Biomarker of Active HHV6 Infection after Allogeneic Hematopoietic Stem Cell Transplantation: Results of a Prospective Study in 213 Patients. *Blood.* 2016; 128(22):3399-3399.
12. Yamane A, Mori T, Suzuki S, et al. Risk factors for developing human herpesvirus 6 (HHV-6) reactivation after allogeneic hematopoietic stem cell transplantation and its association with central nervous system disorders. *Biology of blood and marrow transplantation : journal of the American Society for Blood and Marrow Transplantation.* 2007; 13(1):100-106.
13. van Veen KE, Brouwer MC, van der Ende A, van de Beek D. Bacterial meningitis in hematopoietic stem cell transplant recipients: a population-based prospective study. *Bone marrow transplantation.* 2016; 51(11):1490-1495.
14. Martino R, Bretagne S, Einsele H, et al. Early detection of Toxoplasma infection by molecular monitoring of Toxoplasma gondii in peripheral blood samples after allogeneic stem cell transplantation. *Clinical infectious diseases : an official publication of the Infectious Diseases Society of America.* 2005; 40(1):67-78.
15. Ogata M, Satou T, Kadota J, et al. Human herpesvirus 6 (HHV-6) reactivation and HHV-6 encephalitis after allogeneic hematopoietic cell transplantation: a multicenter, prospective study. *Clinical infectious diseases : an official publication of the Infectious Diseases Society of America.* 2013; 57(5):671-681.
16. DeFilipp Z, Li S, Kempner ME, et al. Phase I Trial of Brentuximab Vedotin for Steroid-Refractory Chronic Graft-versus-Host Disease after Allogeneic Hematopoietic Cell Transplantation. *Biology of Blood and Marrow Transplantation.* 2018; 24(9):1836-1840.
17. Sostak P, Padovan CS, Yousry TA, Ledderose G, Kolb HJ, Straube A. Prospective evaluation of neurological complications after allogeneic bone marrow transplantation. *Neurology.* 2003; 60(5):842-848.
18. Conrad A, Le Marechal M, Dupont D, et al. A matched case-control study of toxoplasmosis after allogeneic haematopoietic stem cell transplantation: still a devastating complication. *Clinical microbiology and infection : the official publication of the European Society of Clinical Microbiology and Infectious Diseases.* 2016; 22(7):636-641.
19. Daly AS, Hasegawa WS, Lipton JH, Messner HA, Kiss TL. Transplantation-associated thrombotic microangiopathy is associated with transplantation from unrelated donors, acute graft-versus-host disease and venoocclusive disease of the liver. *Transfusion and apheresis science : official journal of the World Apheresis Association : official journal of the European Society for Haemapheresis.* 2002; 27(1):3-12.
20. Irle C, Deeg HJ, Buckner CD, et al. Marrow transplantation for leukemia following fractionated total body irradiation. A comparative trial of methotrexate and cyclosporine. *Leukemia research.* 1985; 9(10):1255-1261.

Suppl. Table 5. Retrospective cohort studies characteristics

First author	Year	N	Type	Subtype	n	Onset day	Neuro-deaths	Follow-up	ROB (NOS)
Retrospective cohorts									
Labrador[1]	2015	491	Cerebrovascular	intracranial hemorrhage	12	na	na	33	fair 5/9
Eiten[2]	2015	200	Cerebrovascular	intracranial hemorrhage	4	na	4	14	fair 5/9
Barba[3]	2009	191	Cerebrovascular, peripheral, drug-related, infectious	cyclosporine (8), busulfan (1), foscarnet (1), fungal (1), viral (5)	27	late	15	48	fair 6/9
Scordo[4]	2018	131	Cerebrovascular, higher cortical functions, other	cognitive disturbances (3), leukoencephalopathy (1), seizure (1), stroke (1), other nervous system disorder (5)	11	late	na	36	fair 6/9
Ting-An[5]	2019	459	Cerebrovascular	intracranial hemorrhage/ cerebrovascular infarction	20	late	9	22.1	fair 6/9
Openshaw ***[6]**	2008	1838	Cerebrovascular	iatrogeneic	13	early	na	25	fair 5/9
Hentschke ***[7]**	1999	272	Cerebrovascular	iatrogeneic	3	early	1	na	high risk 4/9
Ke[8]	2018	810	Cerebrovascular, Immune-mediated, Drug-related, Infectious, Metabolic, Other, TA-TMA	cerebrovascular (20), TA-TMA (10), antibiotics (3), calcineurin inhibitors (23), methotrexate (2), metabolic (19), viral (12), fungal (4), bacterial (2), unexplained encephalopathy (3), cGVHD (1), demyelinating diseases (6), cytokine release syndrome (3)	108	early	na	20.5	fair 6/9
Simon ***[9]**	2016	1204	CNS: cerebrovascular, infectious, relapse, other		101	late	28	na	high risk 4/9
Zhang[10]	2013	1213	CNS: drug-related, infectious, cerebrovascular, metabolic, CNS relapse, other	cyclosporine, tacrolimus, imipenem, cilastatin sodium foscarnet, infectious (14), metabolic (11), cerebrovascular diseases (5), leukemic infiltration of CNS (3), recurrence of epilepsy, unknown causes	51	na	na	na	fair 5/9
Narimatsu[11]	2009	77	CNS: drug-related, TA-TMA	cyclosporine (5), tacrolimus (2)	10	early	4	na	fair 5/9

Kageyama[12]	2014	723	CNS: infectious, cerebrovascular, TA-TMA, other	viral (56), bacterial (10)	137	early	na	26	fair 5/9
Siegal[13]	2007	302	CNS: metabolic, cerebrovascular, infectious, other	bacterial (1), viral (2), fungal (2) , brain abscess of unknown origin (1)	74	early	na	na	fair 5/9
Colombo[14]	2017	452	CNS: Cerebrovascular, infectious, immune-mediated, other	cerebral TA-TMA (6), intraparenchymal hemorrhages (1), middle-small vessels sclerosis and atrophy (1), bacterial (2), viral (2), protozoan (2), fungal (3), PRES (2), vasculitis (2), toxicity of radiotherapy (2), uncertain (7)	30	late	18	15	fair 6/9
Avivi[15]	2004	85	Drug-related	alemtuzumab	6	late	4	12	high risk 4/9
Sienkiewicz[16]	2017	30	Drug-related	Voriconazole	11	na	na	na	high risk 4/9
Chamoun[17]	2019	54	Drug-related	tacrolimus	1	early	na	1	fair 5/9
Nevill[18]	1991	70	Drug-related	busulfan-cyclophosphamide conditioning regimen	4	na	na	12	fair 5/9
Schmidt[19]	2015	146	Drug-related	calcineurin inhibitors	7	early	0	42	high risk 4/9
Openshaw *[20]	1997	582	Drug-related	cyclosporine and ganciclovir	4	early	0	27.4	high risk 4/9
Litzow[21]	2002	581	Drug-related		BuCy: 11/373, CyTBI: 0/194	na	na	55 BuCy, 45 CyTBI	fair 5/9
Bartynski[22]	2005	290	Drug-related	cyclosporine and FK-506	21	early	na	na	high risk 4/9
Kalhs[23]	1995	84	Drug-related	cyclocporine-methylprednizolone	CsA-MP: 3/14, CsA-MTX: 0/70	early	3	na	fair 5/9
Posada[24]	2017	14	Drug-related	tacrolimus and CsA	2	na	0	19	fair 6/9
Sala*[25]	2018	1256	Drug-related	calcineurin inhibitors	60	late	na	48	fair 5/9
Porrazzo[26]	2018	11	Drug-related	micafungin	1	early	0	na	high risk 4/9

Takatsuka[27]	1998	7	Drug-related		2	early	2	na	high risk 4/9
Akiyama[28]	2018	70	Drug-related	busulvan, phenyntoin	1	early	na	na	fair 5/9
Wong *[29]	2003	642	Drug-related	tacrolimus	10	early	6	na	high risk 4/9
Benz[30]	2014	250	Drug-related	cerebrovascular	6	na	na	na	fair 5/9
Vu *[31]	2007	43	Drug-related, Infectious	viral, alemtuzumab	5	early	1	na	high risk 4/9
Horowitz[32]	2012	161	Drug-related, infectious, cerebrovascular, metabolic, others		49	early	na	12	fair 5/9
Gifford[33]	2014	99	Higher cortical functions, drug-related, peripheral, cerebrovascular	tacrolimus (1)	11	late	1	74	fair 6/9
Hirano[34]	2017	46	Immune-mediated	CIDP	3	early(1), late(2)	0	na	high risk 4/9
Oda[35]	2009	1967	Immune-mediated	PNS-GVHD	9	late	0	32	fair 5/9
Ebihara *[36]	2018	459	Immune-mediated	non-infectious myelopathy,peripheral neuropathy	8	early	0	17	fair 5/9
Delios[37]	2012	1484	Immune-mediated	ADEM, acute inflammatory demyelinating polyradiculopathy	7	late	na	na	fair 5/9
Wen *[38]	1997	793	Immune-mediated	Guillain-Barré	4	late	0	na	high risk 4/9
Zerr[39]	2005	110	Infectious	viral	4	early	na	3	fair 6/9
Hanajiri[40]	2017	353	Infectious	viral (10), bacterial (6), protozoan (1)	17	early	6	39	fair 6/9
Inui[41]	2018	73	Infectious	viral	7	early	0	na	fair 5/9
Ogata[42]	2017	6593	Infectious	viral	145	early	19	55	fair 6/9
Hill[43]	2012	1344	Infectious	viral (19)	19	early	5	na	fair 6/9
Mata[44]	2008	15	Infectious	viral (2)	2	early	2	na	high risk 4/9
Sakai *[45]	2011	197	Infectious	viral (8)	8	early	0	na	fair 5/9
Avivi[46]	2012	171	Infectious	viral (2)	2	early	1	na	fair 5/9

Busemann *[47]	2011	155	Infectious	protozoan(3)	3	early	3	na	high risk 4/9
Candoni[48]	2009	286	Infectious	fungal	6	early	na	na	fair 5/9
Mori[49]	2010	228	Infectious	viral	13	early	1	na	fair 5/9
Muta *[50]	2009	5484	Infectious	viral	23	early	2	na	high risk 4/9
Seeley *[51]	2007	584	Infectious	viral	9	early	na	na	high risk 4/9
Ueki[52]	2017	320	Infectious	viral	5	early	0	na	fair 5/9
Wolf *[53]	2003	138	Infectious	viral	2	early	2	na	high risk 4/9
Bautista *[54]	2012	70	Infectious	protozoan	3	early	0	na	high risk 4/9
Kouba *[55]	2009	344	Infectious	viral	3	early	0	na	high risk 4/9
Matsuo *[56]	2007	925	Infectious	protozoan	2	early (1), late (1)	1	na	high risk 4/9
Maschke[57]	1999	571	Infectious	protozoan(20), viral (1), fungal(6)	27	early	7	na	fair 5/9
Aljurf[58]	1999	641	Infectious	bacterial (1)	1	late	na	na	high risk 4/9
Jantunen[59]	2003	455	Infectious	fungal (14)	14	late	14	na	high risk 4/9
Roemer *[60]	2001	301	Infectious	protozoan	6	late	na	na	high risk 4/9
Linder[61]	2019	57	Infectious	fungal	2	early	2	24	high risk 4/9
Hakko[62]	2013	170	Infectious, immune-mediated, metabolic, relapse	protozoan (5)	17	early	2	na	fair 5/9
Dowling[63]	2018	263	Infectious, cerebrovascular, drug-related, relapse, metabolic, peripheral, other	sirolimus (4), bacterial (1), viral (1), protozoan (1)	77	early	2	60	fair 5/9
Kishi[64]	2004	232	Infectious, drug-related, cerebrovascular	fungal (1), cyclosporine (4)	18	early	2	17.5	fair 6/9

Lozano *[65]	2015	89	Infectious, drug-related, cerebrovascular, immune-mediated, other	viral (2), protozoan (1), bacterial (1), fungal (1); voriconazole (1), CSA associated papilledema (1), drug-related polyneuropathy (1)	19	early	5	37	high risk 4/9
Haen *[66]	2016	1204	Infectious, higher cortical functions, cerebrovascular, immune-mediated, relapse, other	viral (11), bacterial (2), protozoan (6)	102	late	28	na	fair 5/9
Wittmann[67]	2015	164	Infectious, PTLD, metabolic, drug-related, other	viral (2), fungal (1)	12	early	3	7.2	high risk 4/10
Chohan *[68]	2003	542	Infectious, relapse, cerebrovascular, drug-related	calcineurin inhibitor (30), fungal (3)	38	early	24	na	high risk 4/9
Nakase[69]	2006	8	Other		2	early	2	na	high risk 4/9
Kurosawa*[70]	2017	435	Peripheral	Peripheral nerve injury	17	late	0	na	high risk 4/9
de Philippis[71]	2018	27	Other		1	early	0	18	high risk 4/9
Seto[72]	2018	230	Other		4	late	na	na	fair 6/9
Omer[73]	2011	250	Other		48	early	na	na	fair 5/9
Sanz[74]	2014	288	PTLD		4	late	4	71	fair 6/9
Famoso[75]	2019	58	Relapse	26 patients undergoing cranial boost and TBI and 32 patients receiving TBI alone	0/6, 6/32	na	na	64	fair 6/9
Hamdi[76]	2014	457	Relapse		18	late	na	36	fair 6/9
Davies [77]	2006	25	Relapse		2	late	na	42	fair 6/9
Ataca Atilla *[78]	2016	137	Relapse		12	late	na	na	high risk 4/9
Kata *[79]	2015	595	Relapse		3	late	na	na	high risk 4/9
Bhamidipati[80]	2017	940	Relapse		15	late	0	7	fair 5/9
Oshima[81]	2008	1226	Relapse		29	late	na	na	fair 5/9
Aoki[82]	2014	5068	Relapse		10	na	na	na	fair 6/9
Goker[83]	2015	131	Relapse		15	na	na	na	fair 5/9

Gavriilaki[84]	2018	758	TA-TMA	26	na	na	23	fair 6/9
Oran[85]	2007	1219	TA-TMA	28	na	na	27	fair 6/9
Cho[86]	2008	148	TA-TMA	7	early	1	30	fair 6/9

ADEM: acute disseminated encephalomyelitis; TA-TMA: transplant-associated thrombotic microangiopathy; PRES: posterior reversible encephalopathy; PTLD: Posttransplant lymphoproliferative disease; CsA: cyclosporine; GVHD: graft-versus-host disease; cGVHD: chronic graft-versus-host disease ; CIDP: chronic inflammatory demyelinating polyneuropathy; CNS: central nervous system; PNS: peripheral nervous system; ROB: Risk Of Bias; TBI: total body irradiation; NOS: Newcastle-Ottawa Scale; na: not available; Follow-up is presented as median in months

1. Labrador J, Lopez-Corral L, Vazquez L, et al. Incidence and risk factors for life-threatening bleeding after allogeneic stem cell transplant. *British journal of haematology.* 2015; 169(5):719-725.

2. Eiten EC, Hashmi S, Litzow MR, Hogan W, Gastineau DA, Patnaik M. Etiology and Spectrum of Non-Relapse Mortality after Reduced Intensity Allogeneic Hematopoietic Stem Cell Transplantation in Adults with Myeloid Neoplasms. *Biology of Blood and Marrow Transplantation.* 2015; 21(2):S355.

3. Barba P, Pinana JL, Valcarcel D, et al. Early and late neurological complications after reduced-intensity conditioning allogeneic stem cell transplantation. *Biology of blood and marrow transplantation : journal of the American Society for Blood and Marrow Transplantation.* 2009; 15(11):1439-1446.

4. Scordo M, Shah GL, Kosuri S, et al. Effects of Late Toxicities on Outcomes in Long-Term Survivors of Ex-Vivo CD34+-Selected Allogeneic Hematopoietic Cell Transplantation. *Biology of Blood and Marrow Transplantation.* 2018; 24(1):133-141.

5. Lin TA, Gau JP, Liu YC, et al. Cerebrovascular disease after allogeneic hematopoietic stem cell transplantation: incidence, risk, and clinical outcome. *International journal of hematology.* 2019; 109(5):584-592.

6. Openshaw H, Ressler JA, Snyder DS. Lumbar puncture and subdural hygroma and hematomas in hematopoietic cell transplant patients. *Bone marrow transplantation.* 2008; 41(9):791-795.

7. Hentschke P, Hagglund H, Mattsson J, et al. Bilateral subdural haematomas following lumbar puncture in three haematopoietic stem cell transplant recipients. *Bone marrow transplantation.* 1999; 24(9):1033-1035.

8. Ke P, Bao X, Qiu H, et al. Central nervous system complications caused by 3-4 grade aGVHD in adult patients occurred in HLA-mismatched recipients majorly after allogeneic hematopoietic stem cell transplantation. *Bone marrow transplantation.* 2019.

9. S.P.) SCMNFCVWKLBWAH. Central nervous system complications after allogeneic hematopoietic cell transplantation - A significant cause of morbidity and mortality. *Oncology research and treatment.* 2016:78-79.

10. Zhang XH, Xu LP, Liu DH, et al. Epileptic seizures in patients following allogeneic hematopoietic stem cell transplantation: a retrospective analysis of incidence, risk factors, and survival rates. *Clinical transplantation.* 2013; 27(1):80-89.

11. Narimatsu H, Miyamura K, Iida H, et al. Early central nervous complications after umbilical cord blood transplantation for adults. *Biology of blood and marrow*

transplantation : journal of the American Society for Blood and Marrow Transplantation. 2009; 15(1):92-100.

12. Kageyama K, Yamamoto H, Yuasa M, et al. Early Central Nervous System Complications after Allogeneic Stem Transplantation: A Single-Center Analysis of 723 Patients Including 456 Cord Blood Recipients. *Blood.* 2014; 124(21):2468-2468.

13. Siegal D, Keller A, Xu W, et al. Central nervous system complications after allogeneic hematopoietic stem cell transplantation: incidence, manifestations, and clinical significance. *Biology of blood and marrow transplantation : journal of the American Society for Blood and Marrow Transplantation.* 2007; 13(11):1369-1379.

14. Colombo AA, Marchioni E, Diamanti L, et al. Neurological Complications Involving the Central Nervous System After Allogeneic Hematopoietic Stem Cell Transplantation During a Period of Evolution in Transplant Modalities: A Cohort Analysis. *Transplantation.* 2017; 101(3):616-623.

15. Avivi I, Chakrabarti S, Kottaridis P, et al. Neurological complications following alemtuzumab-based reduced-intensity allogeneic transplantation. *Bone marrow transplantation.* 2004; 34(2):137-142.

16. Sienkiewicz B, Urbaniak-Kujda D, Dybko J, et al. Adverse drug reactions of voriconazole in relation to CYP2C19 mutations among patients after allogenic hematopoietic stem cell transplantation. *Wspolczesna Onkologia.* 2017; 21:10.

17. Chamoun K, Getz T, Kolk M, et al. Oral Vs Intravenous Tacrolimus Post Allogeneic Stem Cell Transplant: A Retrospective Analysis. *Biology of Blood and Marrow Transplantation.* 2019; 25(3):S117-S118.

18. Nevill TJ, Barnett MJ, Klingemann HG, Reece DE, Shepherd JD, Phillips GL. Regimen-related toxicity of a busulfan-cyclophosphamide conditioning regimen in 70 patients undergoing allogeneic bone marrow transplantation. *Journal of clinical oncology : official journal of the American Society of Clinical Oncology.* 1991; 9(7):1224-1232.

19. Schmidt V, Prell T, Treschl A, Klink A, Hochhaus A, Sayer HG. Clinical Management of Posterior Reversible Encephalopathy Syndrome after Allogeneic Hematopoietic Stem Cell Transplantation: A Case Series and Review of the Literature. *Acta haematologica.* 2016; 135(1):1-10.

20. Openshaw H, Slatkin NE, Smith E. Eye movement disorders in bone marrow transplant patients on cyclosporin and ganciclovir. *Bone marrow transplantation.* 1997; 19(5):503-505.

21. Litzow MR, Perez WS, Klein JP, et al. Comparison of outcome following allogeneic bone marrow transplantation with cyclophosphamide-total body irradiation versus busulphan-cyclophosphamide conditioning regimens for acute myelogenous leukaemia in first remission. *British journal of haematology.* 2002; 119(4):1115-1124.

22. Bartynski WS, Zeigler ZR, Shadduck RK, Lister J. Variable incidence of cyclosporine and FK-506 neurotoxicity in hematopoeitic malignancies and marrow conditions after allogeneic bone marrow transplantation. *Neurocritical care.* 2005; 3(1):33-45.

23. Kalhs P, Brugger S, Schwarzinger I, et al. Microangiopathy following allogeneic marrow transplantation. Association with cyclosporine and methylprednisolone for graft-versus-host disease prophylaxis. *Transplantation.* 1995; 60(9):949-957.

24. Posada Alcon L, Calbacho Robles M, Hernández Pérez P, et al. Calcineurin inhibitor as graft versus host disease prophylaxis in patients with relapsed Multiple myeloma undergoing allogeneic stem cell transplantation. *Bone marrow transplantation.* 2017; 52:379-380.

25. Sala E, Harsdorf SV, Wais V, et al. Central nervous system complications after allogeneic hematopoietic stem cell transplantation: The role of calcineurin inhibitors. *Blood.* 2018; 132.

26. Porrazzo M, Piedimonte M, Campagna A, et al. Primary antifungal prophylaxis with micafungin in hematological malignancies: A single center experience. *HemaSphere.* 2018; 2:929-930.
27. Takatsuka H, Takemoto Y, Okamoto T, et al. The levels of soluble P-selectin, von Willebrand factor and thrombomodulin in patients with neurological complications after allogeneic bone marrow transplantation. *Bone marrow transplantation.* 1998; 21(8):809-813.
28. Akiyama K, Kume T, Fukaya M, et al. Comparison of levetiracetam with phenytoin for the prevention of intravenous busulfan-induced seizures in hematopoietic cell transplantation recipients. *Cancer Chemotherapy and Pharmacology.* 2018; 82(4):717-721.
29. Wong R, Beguelin GZ, de Lima M, et al. Tacrolimus-associated posterior reversible encephalopathy syndrome after allogeneic haematopoietic stem cell transplantation. *British journal of haematology.* 2003; 122(1):128-134.
30. Benz R, Schanz U, Maggiorini M, Seebach JD, Stussi G. Risk factors for ICU admission and ICU survival after allogeneic hematopoietic SCT. *Bone marrow transplantation.* 2014; 49(1):62-65.
31. Vu T, Carrum G, Hutton G, Heslop HE, Brenner MK, Kamble R. Human herpesvirus-6 encephalitis following allogeneic hematopoietic stem cell transplantation. *Bone marrow transplantation.* 2007; 39(11):705-709.
32. Horowitz N, Wittmann T, Rowe JM, Zuckerman T, Avivi I. Similar Frequency and Types of Neurological Complications in Patients Undergoing Matched Related and Matched Unrelated Donor Stem Cell Transplantation for Hematological Malignancies. *Blood.* 2012; 120(21):4515-4515.
33. Gifford G, Sim J, Horne A, Ma D. Health status, late effects and long-term survivorship of allogeneic bone marrow transplantation: a retrospective study. *Internal medicine journal.* 2014; 44(2):139-147.
34. Hirano M, Jimbo K, Ogawa M, et al. Chronic inflammatory demyelinating polyneuropathy in adult T-cell leukemia-lymphoma patients following allogeneic stem cell transplantation. *Bone marrow transplantation.* 2018; 53(11):1470-1473.
35. Oda K, Nakaseko C, Ozawa S, et al. Fasciitis and myositis: an analysis of muscle-related complications caused by chronic GVHD after allo-SCT. *Bone marrow transplantation.* 2009; 43(2):159-167.
36. Ebihara M, Takagi S, Uesaka Y, et al. High-dose intravenous immunoglobulin and immunosuppressive therapy have therapeutic potential for non-infectious myelopathy and peripheral neuropathy following cord blood transplantation. *Blood.* 2018; 132.
37. Delios AM, Rosenblum M, Jakubowski AA, DeAngelis LM. Central and peripheral nervous system immune mediated demyelinating disease after allogeneic hemopoietic stem cell transplantation for hematologic disease. *Journal of neuro-oncology.* 2012; 110(2):251-256.
38. Wen PY, Alyea EP, Simon D, Herbst RS, Soiffer RJ, Antin JH. Guillain-Barre syndrome following allogeneic bone marrow transplantation. *Neurology.* 1997; 49(6):1711-1714.
39. Zerr DM, Corey L, Kim HW, Huang ML, Nguy L, Boeckh M. Clinical outcomes of human herpesvirus 6 reactivation after hematopoietic stem cell transplantation. *Clinical infectious diseases : an official publication of the Infectious Diseases Society of America.* 2005; 40(7):932-940.
40. Hanajiri R, Kobayashi T, Yoshioka K, et al. Central nervous system infection following allogeneic hematopoietic stem cell transplantation. *Hematology/oncology and stem cell therapy.* 2017; 10(1):22-28.

41. Inui Y, Yakushijin K, Okamura A, et al. Human herpesvirus 6 encephalitis in patients administered mycophenolate mofetil as prophylaxis for graft-versus-host disease after allogeneic hematopoietic stem cell transplantation. *Transplant Infectious Disease.* 2019; 21(1).
42. Ogata M, Oshima K, Ikebe T, et al. Clinical characteristics and outcome of human herpesvirus-6 encephalitis after allogeneic hematopoietic stem cell transplantation. *Bone marrow transplantation.* 2017; 52(11):1563-1570.
43. Hill JA, Koo S, Guzman Suarez BB, et al. Cord-blood hematopoietic stem cell transplant confers an increased risk for human herpesvirus-6-associated acute limbic encephalitis: a cohort analysis. *Biology of blood and marrow transplantation : journal of the American Society for Blood and Marrow Transplantation.* 2012; 18(11):1638-1648.
44. Mata S, Guidi S, Nozzoli C, et al. Human herpesvirus 6-associated limbic encephalitis in adult recipients of unrelated umbilical cord blood transplantation. *Bone marrow transplantation.* 2008; 42(10):693-695.
45. Sakai R, Kanamori H, Motohashi K, et al. Long-term outcome of human herpesvirus-6 encephalitis after allogeneic stem cell transplantation. *Biology of blood and marrow transplantation : journal of the American Society for Blood and Marrow Transplantation.* 2011; 17(9):1389-1394.
46. Avivi I, Wittman T, Benyamini N, et al. Reactivation of JC Virus in 171 Allografted Patients: Incidence and Clinical Significance. *Blood.* 2012; 120(21):4148-4148.
47. Busemann C, Ribback S, Zimmermann K, et al. Toxoplasmosis after allogeneic stem cell transplantation--a single centre experience. *Annals of hematology.* 2012; 91(7):1081-1089.
48. Candoni AS, E.; Pagano, L.; Caira, M.; Fanin, R. Incidence and risk factors of proven or probable invasive fungal infections (IFI) in 286 patients undergoing related or unrelated allogeneic bone marrow transplantation. *Haematologica.* 2009; 94:106-107.
49. Mori Y, Miyamoto T, Nagafuji K, et al. High incidence of human herpes virus 6-associated encephalitis/myelitis following a second unrelated cord blood transplantation. *Biology of blood and marrow transplantation : journal of the American Society for Blood and Marrow Transplantation.* 2010; 16(11):1596-1602.
50. Muta T, Fukuda T, Harada M. Human herpesvirus-6 encephalitis in hematopoietic SCT recipients in Japan: a retrospective multicenter study. *Bone marrow transplantation.* 2009; 43(7):583-585.
51. Seeley WW, Marty FM, Holmes TM, et al. Post-transplant acute limbic encephalitis: clinical features and relationship to HHV6. *Neurology.* 2007; 69(2):156-165.
52. Ueki T, Hoshi K, Hiroshima Y, et al. Analysis of five cases of human herpesvirus-6 myelitis among 121 cord blood transplantations. *International journal of hematology.* 2018; 107(3):363-372.
53. Wolf DG, Lurain NS, Zuckerman T, et al. Emergence of late cytomegalovirus central nervous system disease in hematopoietic stem cell transplant recipients. *Blood.* 2003; 101(2):463-465.
54. Bautista G, Ramos A, Fores R, et al. Toxoplasmosis in cord blood transplantation recipients. *Transplant infectious disease : an official journal of the Transplantation Society.* 2012; 14(5):496-501.
55. Kouba MM, M.; Valkova, V.; Hricinova, M.; Soukup, P.; Salek, C.; Mertova, J.; Maaloufova, J.; Hubacek, P.; Cermak, J.; Vitek, A.; Cetkovsky, P. Serious EBV encephalitis in patients after allogeneic stem cell transplantation (allo-SCT) - Series of 3 cases. *Haematologica.* 2009; 94:413.

56. Matsuo Y, Takeishi S, Miyamoto T, et al. Toxoplasmosis encephalitis following severe graft-vs.-host disease after allogeneic hematopoietic stem cell transplantation: 17 yr experience in Fukuoka BMT group. *European journal of haematology.* 2007; 79(4):317-321.
57. Maschke M, Dietrich U, Prumbaum M, et al. Opportunistic CNS infection after bone marrow transplantation. *Bone marrow transplantation.* 1999; 23(11):1167-1176.
58. Aljurf M, Gyger M, Alrajhi A, et al. Mycobacterium tuberculosis infection in allogeneic bone marrow transplantation patients. *Bone marrow transplantation.* 1999; 24(5):551-554.
59. Jantunen E, Volin L, Salonen O, et al. Central nervous system aspergillosis in allogeneic stem cell transplant recipients. *Bone marrow transplantation.* 2003; 31(3):191-196.
60. Roemer E, Blau IW, Basara N, et al. Toxoplasmosis, a severe complication in allogeneic hematopoietic stem cell transplantation: successful treatment strategies during a 5-year single-center experience. *Clinical infectious diseases : an official publication of the Infectious Diseases Society of America.* 2001; 32(1):E1-8.
61. Linder KA, McDonald PJ, Kauffman CA, Revankar SG, Chandrasekar PH, Miceli MH. Invasive aspergillosis in patients following umbilical cord blood transplant. *Bone marrow transplantation.* 2019; 54(2):308-311.
62. Hakko E, Ozkan HA, Karaman K, Gulbas Z. Analysis of cerebral toxoplasmosis in a series of 170 allogeneic hematopoietic stem cell transplant patients. *Transplant infectious disease : an official journal of the Transplantation Society.* 2013; 15(6):575-580.
63. Dowling MR, Li S, Dey BR, et al. Neurologic complications after allogeneic hematopoietic stem cell transplantation: Risk factors and impact. *Bone marrow transplantation.* 2018; 53(2):199-206.
64. Kishi Y, Miyakoshi S, Kami M, et al. Early central nervous system complications after reduced-intensity stem cell transplantation. *Biology of blood and marrow transplantation : journal of the American Society for Blood and Marrow Transplantation.* 2004; 10(8):561-568.
65. Lozano SI, M.; Aoiz, I.; Arregui, P.; Zabalza, A.; Hamdi, M.; Quispe, I.; Alvarellos, M.;, Montoya MCG, P.; Gorosquieta, A.; Perez Equiza, K.; Erro, E.; Olavarria, E. High incidence of neurological complications after allogeneic stem cell transplantation: Association with transplant related mortality. *Bone marrow transplantation.* 2015; 50:S430-S431.
66. Haen SPZ, M.; Mirza, N.; Simon, C. M.; Korn, A.; Faul, C.; Vogel, W.; Rammensee,H. G.; Kanz, L.; Bethge, W. Disease and therapy associated complications of the central nervous systemafter allogeneic hematopoietic celltransplantation-a significant proportion ofpatients will suffer from permanent damage. *Blood.* 2016; 128(22):2016.
67. Wittmann T, Horowitz N, Benyamini N, et al. JC polyomavirus reactivation is common following allogeneic stem cell transplantation and its preemptive detection may prevent lethal complications. *Bone marrow transplantation.* 2015; 50(7):984-991.
68. Chohan R, Vij R, Adkins D, et al. Long-term outcomes of allogeneic stem cell transplant recipients after calcineurin inhibitor-induced neurotoxicity. *British journal of haematology.* 2003; 123(1):110-113.
69. Nakase K, Hara M, Kozuka T, Tanimoto K, Nawa Y. Bone marrow transplantation from unrelated donors for patients with adult T-cell leukaemia/lymphoma. *Bone marrow transplantation.* 2006; 37(1):41-44.

70. Kurosawa S, Toya T, Igarashi A, et al. Peripheral nerve injury following allogeneic hematopoietic stem cell transplantation. *Journal of the Neurological Sciences.* 2017; 381:615.
71. De Philippis C, Mariotti J, Bramanti S, et al. Early transplant related complications in Hodgkin lymphoma patients receiving PD-1 inhibitors before allogeneic stem cell transplantation. *HemaSphere.* 2018; 2:46-47.
72. Seto A, Atsuta Y, Kawashima N, Ozawa Y, Miyamura K, Kiyoi H. Impact of hospital length of stay on the risk of readmission and overall survival after allogeneic stem cell transplantation. *International journal of hematology.* 2018; 108(3):290-297.
73. Omer TA, Doherty C. Posterior reversible encephalopathy syndrome (PRES) complicating the 'legal high' mephedrone. *BMJ case reports.* 2011; 2011.
74. Sanz J, Arango M, Senent L, et al. EBV-associated post-transplant lymphoproliferative disorder after umbilical cord blood transplantation in adults with hematological diseases. *Bone marrow transplantation.* 2014; 49(3):397-402.
75. Famoso JM, Grow JL, Laughlin B, Katsanis E, Stea B. The Impact of Low-Dose Cranial Boost on the Long-Term Outcomes of Adult Patients with High-Risk Acute Lymphoblastic Leukemia Undergoing Total Body Irradiation and Allogeneic Hematopoietic Stem Cell Transplantation. *Practical radiation oncology.* 2018.
76. Hamdi A, Mawad R, Bassett R, et al. Central nervous system relapse in adults with acute lymphoblastic leukemia after allogeneic hematopoietic stem cell transplantation. *Biology of blood and marrow transplantation : journal of the American Society for Blood and Marrow Transplantation.* 2014; 20(11):1767-1771.
77. Davies JK, Taussig DC, Oakervee H, et al. Long-term follow-up after reduced-intensity conditioning allogeneic transplantation for acute myeloid leukemia/myelodysplastic syndrome: late CNS relapses despite graft-versus-host disease. *Journal of clinical oncology : official journal of the American Society of Clinical Oncology.* 2006; 24(14):e23-25.
78. G.; Ozcan M. AAPAECBSKTSTPAOAHBMIOG. Management and outcome of central nervous system (CNS) involvement in acute lymphoblastic leukemia (ALL): Pre and post transplant. *Haematologica.* 2016:356.
79. Physicians abstracts. *Bone marrow transplantation.* 2015; 50 Suppl 1:S1-S96.
80. Bhamidipati PK, Ghobadi A, Se-Young H, et al. Clinical characteristics of isolated CNS relapse after allogeneic transplantation for AML - Poor outcomes despite longer time to relapse. *Blood.* 2017; 130.
81. Oshima K, Kanda Y, Yamashita T, et al. Central nervous system relapse of leukemia after allogeneic hematopoietic stem cell transplantation. *Biology of blood and marrow transplantation : journal of the American Society for Blood and Marrow Transplantation.* 2008; 14(10):1100-1107.
82. Aoki J, Ishiyama K, Taniguchi S, et al. Outcome of allogeneic hematopoietic stem cell transplantation for acute myeloid leukemia patients with central nervous system involvement. *Biology of blood and marrow transplantation : journal of the American Society for Blood and Marrow Transplantation.* 2014; 20(12):2029-2033.
83. Goker HG, G.; Demiroglu, H.; Malkan, U. Y.; Haznedaroglu, I.; Sayinalp, N.; Etgul, S.; Eliacik, E.; Aslan, T.; Isik, A.; Aydin, S.; Yalcin, S.; Aksu, S.; Ozcebe, O. I.; Buyukasik, Y. Extramedullary relapses after allogeneic stem cell transplantation for leukemia: Clinical characteristics, cumulative incidence and risk factors. *Journal of Clinical Oncology.* 2015; 33(15).
84. Gavriilaki E, Sakellari I, Mallouri D, et al. Transplant-associated thrombotic microangiopathy: Incidence, prognostic factors, morbidity and mortality in allogeneic hematopoietic cell transplantation. *HemaSphere.* 2018; 2:313.

85. Oran B, Donato M, Aleman A, et al. Transplant-associated microangiopathy in patients receiving tacrolimus following allogeneic stem cell transplantation: risk factors and response to treatment. *Biology of blood and marrow transplantation : journal of the American Society for Blood and Marrow Transplantation.* 2007; 13(4):469-477.

86. Cho BS, Min CK, Eom KS, et al. Clinical impact of thrombotic microangiopathy on the outcome of patients with acute graft-versus-host disease after allogeneic hematopoietic stem cell transplantation. *Bone marrow transplantation.* 2008; 41(9):813-820.

Suppl. Table 6. Case series studies characteristics

First author	Year	N	Type	Subtype	n	Onset day	Neuro-deaths	Follow-up	ROB (NOS)
Case series									
Kawakami[1]	2013	na	Drug-related	nelarabine	6	early	0	na	low risk
Teive[2]	2001	na	Drug-related	CsA	7	na	0	na	low risk
Hammerstrom[3]	2013	na	Drug-related	tacrolimus (19)	19	early	na	na	fair
Hilgendorf[4]	2008	na	Drug-related		5	early	na	na	fair
Benakli[5]	2012	na	Immune-mediated	GVHD	6	late	0	na	fair
Padovan[6]	1999	na	Immune-mediated	GVHD	5	late	1	na	low risk
Ruggiu[7]	2017	na	Immune-mediated	GVHD	7	late	4	na	low risk
Kraus[8]	2012	na	Immune-mediated	GVHD	32	late	na	12	low risk
Stefanou[9]	2017	na	Immune-mediated		61	late	0	na	low risk
Bilic[10]	2016	na	Immune-mediated	GVHD	27	late	na	na	fair
Mannina[11]	2016	na	Infectious	viral	14	early	na	na	fair
Miaux[12]	1995	na	Infectious	fungal	5	early	5	na	low risk
Epker[13]	2009	na	Infectious	viral	1	na	1	na	low risk
Umezawa[14]	2014	na	Infectious	viral	5	late	1	na	low risk
Kleinschmidt-DeMasters[15]	2004	na	Infectious	viral	2	Early (1), late(1)	1	na	low risk
Saleh Omar[16]	2017	na	Infectious	viral	9	early	0	40	low risk
Shannon[17]	2016	na	Infectious	bacterial	6	late	2	na	low risk
Greco[18]	2016	na	Infectious	viral	10	early	na	na	low risk
Kuo[19]	2009	na	Metabolic		2	late	2	na	fair

Castellano-Sanchez[20]	2004	na	PTLD	2	late	2	na	low risk
Wu[21]	2016	na	PTLD	14	early	4	22	low risk

PTLD: Posttransplant lymphoproliferative disease; CsA: cyclosporine; GVHD: graft-versus-host disease; ROB: Risk Of Bias; NOS: Newcastle-Ottawa Scale; na: not available; Follow-up is presented as median in months

Supplementary table 7. GRADE tool

Certainty assessment							Number of patients	Effect	Certainty
№ of studies	Study design	Risk of bias	Inconsistency	Indirectness	Imprecision	Other considerations			
**Incidence of neurologic complications**									
78	observational studies	very serious [a]	serious [b]	not serious	serious [c]	publication bias strongly suspected [d]	37450	1415 (6.2%, 95%CI 4.8-7.7)	⨁◯◯◯ Very low

Explanations

a. most studies of high risk of bias

b. high heterogeneity in results

c. wide confidence intervals CI in results, small studies

d. small, low quality, one arm studies, funnel plot asymmetry

1. Kawakami M, Taniguchi K, Yoshihara S, et al. Irreversible neurological defects in the lower extremities after haploidentical stem cell transplantation: possible association with nelarabine. *American journal of hematology.* 2013; 88(10):853-857.
2. Teive HA, Brandi IV, Camargo CH, et al. Reversible posterior leucoencephalopathy syndrome associated with bone marrow transplantation. *Arquivos de neuro-psiquiatria.* 2001; 59(3-B):784-789.
3. Hammerstrom AE, Howell J, Gulbis A, Rondon G, Champlin RE, Popat U. Tacrolimus-associated posterior reversible encephalopathy syndrome in hematopoietic allogeneic stem cell transplantation. *American journal of hematology.* 2013; 88(4):301-305.
4. Hilgendorf I, Wolff D, Junghanss C, et al. Neurological complications after intrathecal liposomal cytarabine application in patients after allogeneic haematopoietic stem cell transplantation. *Annals of hematology.* 2008; 87(12):1009-1012.
5. Benakli MAN, R.; Baazizi, M.; Mehdid, F.; Rahmoune, N.; Belhadj, R.; Talbi, A.; Ait ouali, D.; Hamladji, R. M. Intravenous gammaglobulin therapy for neurologic manifestations of chronic GvHD after allogeneic haematopoietic stem cell transplantation. *Bone marrow transplantation.* 2012(47).
6. Padovan CS, Bise K, Hahn J, et al. Angiitis of the central nervous system after allogeneic bone marrow transplantation? *Stroke.* 1999; 30(8):1651-1656.
7. Ruggiu M, Cuccuini W, Mokhtari K, et al. Case report: Central nervous system involvement of human graft versus host disease: Report of 7 cases and a review of literature. *Medicine.* 2017; 96(42):e8303.
8. Kraus PD, Wolff D, Grauer O, et al. Muscle cramps and neuropathies in patients with allogeneic hematopoietic stem cell transplantation and graft-versus-host disease. *PloS one.* 2012; 7(9):e44922.
9. Stefanou MI, Bischof F. Central and peripheral nervous system immune-mediated demyelinating disease after allogeneic hematopoietic stem cell transplantation. *Journal of neuroimmunology.* 2017; 307:74-81.
10. Bilic E, Delimar V, Desnica L, et al. High prevalence of small- and large-fiber neuropathy in a prospective cohort of patients with moderate to severe chronic GvHD. *Bone marrow transplantation.* 2016; 51(11):1513-1517.
11. Physicians Poster SessionsPoster Session / Day 1. *Bone marrow transplantation.* 2016; 51 Suppl 1:S110-313.
12. Miaux Y, Ribaud P, Williams M, et al. MR of cerebral aspergillosis in patients who have had bone marrow transplantation. *AJNR. American journal of neuroradiology.* 1995; 16(3):555-562.
13. Epker JL, van Biezen P, van Daele PL, van Gelder T, Vossen A, van Saase JL. Progressive multifocal leukoencephalopathy, a review and an extended report of five patients with different immune compromised states. *European journal of internal medicine.* 2009; 20(3):261-267.
14. Umezawa Y, Kakihana K, Oshikawa G, et al. Clinical features and risk factors for developing varicella zoster virus dissemination following hematopoietic stem cell transplantation. *Transplant infectious disease : an official journal of the Transplantation Society.* 2014; 16(2):195-202.
15. Kleinschmidt-DeMasters BK, Marder BA, Levi ME, et al. Naturally acquired West Nile virus encephalomyelitis in transplant recipients: clinical, laboratory, diagnostic, and neuropathological features. *Archives of neurology.* 2004; 61(8):1210-1220.
16. Saleh OA, Bryson A, Alkharabsheh O, Razonable R. Title: Poor long-term outcomes of patients with human herpesvirus 6 encephalitis. *Blood.* 2017; 130.
17. Shannon K, Pasikhova Y, Ibekweh Q, Ludlow S, Baluch A. Nocardiosis following hematopoietic stem cell transplantation. *Transplant infectious disease : an official journal of the Transplantation Society.* 2016; 18(2):169-175.
18. Greco R, Crucitti L, Noviello M, et al. Human Herpesvirus 6 Infection Following Haploidentical Transplantation: Immune Recovery and Outcome. *Biology of blood and marrow transplantation : journal of the American Society for Blood and Marrow Transplantation.* 2016; 22(12):2250-2255.
19. Kuo SH, Debnam JM, Fuller GN, de Groot J. Wernicke's encephalopathy: an underrecognized and reversible cause of confusional state in cancer patients. *Oncology.* 2009; 76(1):10-18.
20. Castellano-Sanchez AA, Li S, Qian J, Lagoo A, Weir E, Brat DJ. Primary central nervous system posttransplant lymphoproliferative disorders. *Am J Clin Pathol.* 2004; 121(2):246-253.
21. Wu M, Sun J, Zhang Y, et al. Intrathecal rituximab for EBV-associated post-transplant lymphoproliferative disorder with central nervous system involvement unresponsive to intravenous rituximab-based treatments: a prospective study. *Bone marrow transplantation.* 2016; 51(3):456-458.
